# Efficacy of different surgical treatments for management of anal fistula: a network meta-analysis

**DOI:** 10.1007/s10151-023-02845-8

**Published:** 2023-07-17

**Authors:** S. Bhat, W. Xu, C. Varghese, N. Dubey, C. I. Wells, C. Harmston, G. O’Grady, I. P. Bissett, A. Y. Lin

**Affiliations:** 1 Surgical and Translational Research Centre, Department of Surgery, The University of Auckland, Auckland, New Zealand; 2 Department of Surgery, Te Whatu Ora MidCentral, Palmerston North, New Zealand; 3 Department of Surgery, Te Whatu Ora Te Toka Tumai, Whangārei, New Zealand; 4 Department of General Medicine, Tauranga Hospital, Te Whatu Ora, Tauranga, New Zealand; 5 Department of Surgery, Te Whatu Ora Te Toka Tumai, Auckland, New Zealand; 6 Auckland Bioengineering Institute, The University of Auckland, Auckland, New Zealand; 7 Department of Surgery and Anaesthesia, University of Otago, Wellington, New Zealand; 8 Department of Surgery, Wellington Regional Hospital, Te Whatu Ora, Wellington, New Zealand

**Keywords:** Fistula-in-ano, Complex, Sphincter preserving, Healing, Incontinence

## Abstract

**Purpose:**

Currently, the anal fistula treatment which optimises healing and preserves bowel continence remains unclear. The aim of our study was to compare the relative efficacy of different surgical treatments for AF through a network meta-analysis.

**Methods:**

Systematic searches of MEDLINE, EMBASE and CENTRAL databases up to October 2022 identified randomised controlled trials (RCTs) comparing surgical treatments for anal fistulae. Fistulae were classified as simple (inter-sphincteric or low trans-sphincteric fistulae crossing less than 30% of the external anal sphincter (EAS)) and complex (high trans-sphincteric fistulae involving more than 30% of the EAS). Treatments evaluated in only one trial were excluded from the primary analyses to minimise bias. The primary outcomes were rates of success in achieving AF healing and bowel incontinence.

**Results:**

Fifty-two RCTs were included. Of the 14 treatments considered, there were no significant differences regarding short-term (6 months or less postoperatively) and long-term (more than 6 months postoperatively) success rates between any of the treatments in patients with both simple and complex anal fistula. Ligation of the inter-sphincteric fistula tract (LIFT) ranked best for minimising bowel incontinence in simple (99.1% of comparisons; 3 trials, *n* = 70 patients) and complex anal fistula (86.2% of comparisons; 3 trials, *n* = 102 patients).

**Conclusions:**

There is insufficient evidence in existing RCTs to recommend one treatment over another regarding their short and long-term efficacy in successfully facilitating healing of both simple and complex anal fistulae. However, LIFT appears to be associated with the least impairment of bowel continence, irrespective of AF classification.

**Supplementary Information:**

The online version contains supplementary material available at 10.1007/s10151-023-02845-8.

## Introduction

Anal fistula is a pathological connection between the anal canal and perianal skin, which can cause severe pain, perianal swelling, bleeding, and purulent discharge [1–3]. Although new strategies for classifying anal fistula have been proposed [4], fistulae are commonly categorised as “simple” and “complex” on the basis of their anatomical course relative to the external anal sphincter (EAS) [5]. Simple anal fistula include inter-sphincteric or low trans-sphincteric fistulae, which cross less than 30% of the EAS [2, 6]. Complex anal fistula include high trans-sphincteric fistulae, which have greater than 30% involvement of the EAS, supra-sphincteric, extra-sphincteric, or horseshoe fistulae, fistulae with secondary tracts, anterior fistulae (in women), or those associated with inflammatory bowel disease, radiation, malignancy, pre-existing faecal incontinence and chronic diarrhoea [5, 7].

Surgical management of anal fistula is decided on the basis of patient factors as well as anatomical complexity relative to the EAS [8]. Anal fistulotomy is effective for managing simple anal fistula, although it places patients at risk of bowel incontinence due to partial or complete division of the anal sphincter complex [9, 10]. For this reason, several sphincter-preserving treatments have been developed, particularly for complex anal fistula. These include loose setons [11], fibrin glue [12], collagen plug [13, 14], anorectal advancement flap [15–17], ligation of the inter-sphincteric fistula tract [18–20], fistula laser closure [21] and, more recently, mesenchymal adipose-derived stem cell injections [22–24]. These treatments are associated with less impairment of bowel function, although their healing rates vary considerably. Consensus regarding which treatment reliably provides the highest rate of healing whilst also preserving bowel continence is lacking.

Network meta-analysis (NMA) allows for a coherent ranking of multiple treatments through direct comparisons, using evidence presented in several randomised controlled trials (RCTs), and statistically derived indirect comparisons [25, 26]. The aim of this study was therefore to compare the relative efficacy between different surgical treatments for simple and complex anal fistula through a NMA, which could assist surgeons in counselling patients about the risks and benefits of each treatment and in deciding on the most suitable option for managing anal fistula.

## Methods

The protocol for this review was prospectively recorded on PROSPERO (ID CRD42021288310) [27]. The study was performed in accordance with the Preferred Reporting Items for Systematic reviews and Meta-Analyses (PRISMA) guidelines, with extension for NMA (the PRISMA-NMA checklist is shown in Supplementary Appendix 1) [28].

### Search strategy

The MEDLINE, EMBASE, and Cochrane Controlled Register of Trials (CENTRAL) databases were systematically searched in December 2021, with results updated to October 2022. Boolean operators (“AND”/“OR”) were used to combine keywords and Medical Subject Headings (MeSH) for different anal fistula treatments (Supplementary Appendix S2).

### Study selection

All RCTs comparing at least two surgical treatments in patients undergoing elective surgery for managing anal fistula were eligible for inclusion. Simple anal fistula included inter-sphincteric and low trans-sphincteric fistula crossing less than 30% of the EAS, and complex anal fistula included high trans-sphincteric fistulae (involving more than 30% of the EAS), supra-sphincteric, extra-sphincteric, or horseshoe fistulae, fistulae with secondary tracts, anterior fistulae (in women), and fistulae secondary to pre-existing faecal incontinence [5]. For the purposes of our analyses, recurrent anal fistula were classified as either simple or complex according to their anatomical course and/or characteristics. Studies were restricted to those conducted in human, adult patients (at least 18 years old), although there were no restrictions on publication date or language. One investigator (WX) was able to translate relevant non-English studies to facilitate their inclusion in the final review [29, 30]. Titles/abstracts and potentially relevant full-texts were independently reviewed after removal of duplicate records [31, 32], with any discrepancies settled by discussion and with input from senior authors as required.

Studies with non-randomised designs (e.g. prospective/retrospective cohort studies, case–control studies, case series and case reports), where anal fistulae were managed nonoperatively, and those conducted in paediatric patients (less than 18 years old) or in patients with anal fistula secondary to inflammatory bowel disease (IBD), radiation, malignancy, and chronic diarrhoea, were excluded. Editorial letters, book chapters, conference abstracts, and trial protocols were also excluded, as were records in which the full text could not be sourced. Reference lists of relevant reviews were screened to identify additional studies, although the studies themselves were excluded.

### Data extraction

Extracted data included information on study characteristics (first author, publication year, trial location, treatment comparisons, follow-up period), patient demographic characteristics (number of patients randomised and subsequently treated, age, sex), and anal fistula characteristics (simple versus complex classification, location, and length of the tract). Accuracy of these data were validated by two reviewers independently (WX, CV). Any inconsistencies in the data were resolved via discussion and with mediation by a senior author if necessary.

Corresponding authors were contacted to resolve instances of ambiguous data [33]. Estimates of the mean and standard deviation (SD) were derived for continuous data reported as the median and range (or interquartile range) using validated methods [34–36]. WebPlotDigitizer (Version 4.5; Pacifica, California, USA) was used to extract data that were reported in the form of graphs and/or figures [37].

### Quality assessment

The Cochrane Collaboration’s Risk of Bias 2.0 (ROB2) tool was used by three reviewers to independently evaluate the methodological quality of included RCTs (SB, WX, ND) [38]. Discrepancies in study quality were discussed between the reviewers until consensus was reached.

### Outcome measures

The main outcomes were rates of success and bowel incontinence. Success was defined as complete healing of the anal fistula without recurrence or persistence of symptoms on follow-up, and was measured in the short term (6 months or less after surgery) and long term (more than 6 months after surgery). Healing was defined on the basis of clinical examination, and/or endoanal ultrasound scan (USS) or pelvic magnetic resonance imaging (MRI) findings, or was self-reported by patients on the basis of the resolution of symptoms at follow-up. Bowel incontinence was defined as incontinence to either gas, liquid and/or solid stool. Secondary outcomes included hospital length of stay (LOS) and overall postoperative complication rates. These outcomes were analysed separately for patients with simple and complex anal fistula.

### Statistical analysis

An intention-to-treat Bayesian NMA with a non-informative prior distribution was performed in RStudio (Version 4.2.2; Posit PBC, Boston, Massachusetts, USA).

All direct treatment comparisons were visually represented through network plots for each outcome. The size of each node correlated with the number of patients randomised to each treatment, and the thickness of each line connecting two nodes was proportional to the number of RCTs comparing those two treatments. Fistulectomy was used as the reference treatment for comparisons in simple anal fistulae, while advancement flap was used in cases of complex anal fistulae. Continuity corrections of one were applied to both the numerator and denominator of each treatment arm to facilitate inclusion of categorical outcomes with zero observed events [39]. Effect sizes were reported as the log OR for categorical outcomes [40, 41], and mean difference (MD) for continuous outcomes, with their respective 95% credible interval (CrI). Differences were considered statistically significant if the 95% CrI did not cross zero (the no-effect line). Treatments assessed in only one trial, which were not connected to at least two treatments through the network, were excluded from the primary analysis to minimise bias resulting from single-trial effects. For each outcome, results were illustrated through a rankogram plot, surface under the cumulative ranking (SUCRA) curve, heat plot, and forest plot. Rankograms were used to visualise the relative effectiveness of each treatment as stacked bar plots representing the probability of each intervention to achieve each rank. SUCRA curves illustrate the relative ranking probability (i.e. SUCRA values) of each intervention; the horizontal axis is the probability a treatment would fall within that rank [42]. SUCRA values ranged from 0 to 100%, with higher values indicating a greater probability of being the best performing treatment for a particular outcome [43]. Heat plots were used to illustrate the effect size (OR or MD with their corresponding 95% CrI) for each treatment compared with one another, while forest plots were used to display the effect size of each treatment relative to a treatment of reference [42]. The *I*^2^ statistic was used to quantify the percentage of total variability in effect size across trials that is attributable to true heterogeneity rather than chance, and was calculated for each direct comparison of treatments [44].

For NMA results to be valid, the conditions of consistency and transitivity must be maintained. Consistency refers to the assumption that effect sizes derived from indirect and direct comparisons are similar [45, 46]. Transitivity refers to the assumption that potential modifiers of treatment effect sizes are equally distributed across all RCTs [45]. This was assessed by comparing demographic data (e.g. age and sex) of patients randomised to each treatment, and geographic study location(s) between the different treatments analysed.

### Sensitivity analysis

Sensitivity analyses were performed by assessing all treatments, to examine the impact of bias introduced when treatments were analysed in only a single study.

## Results

### Search results

**Fig. 1:**
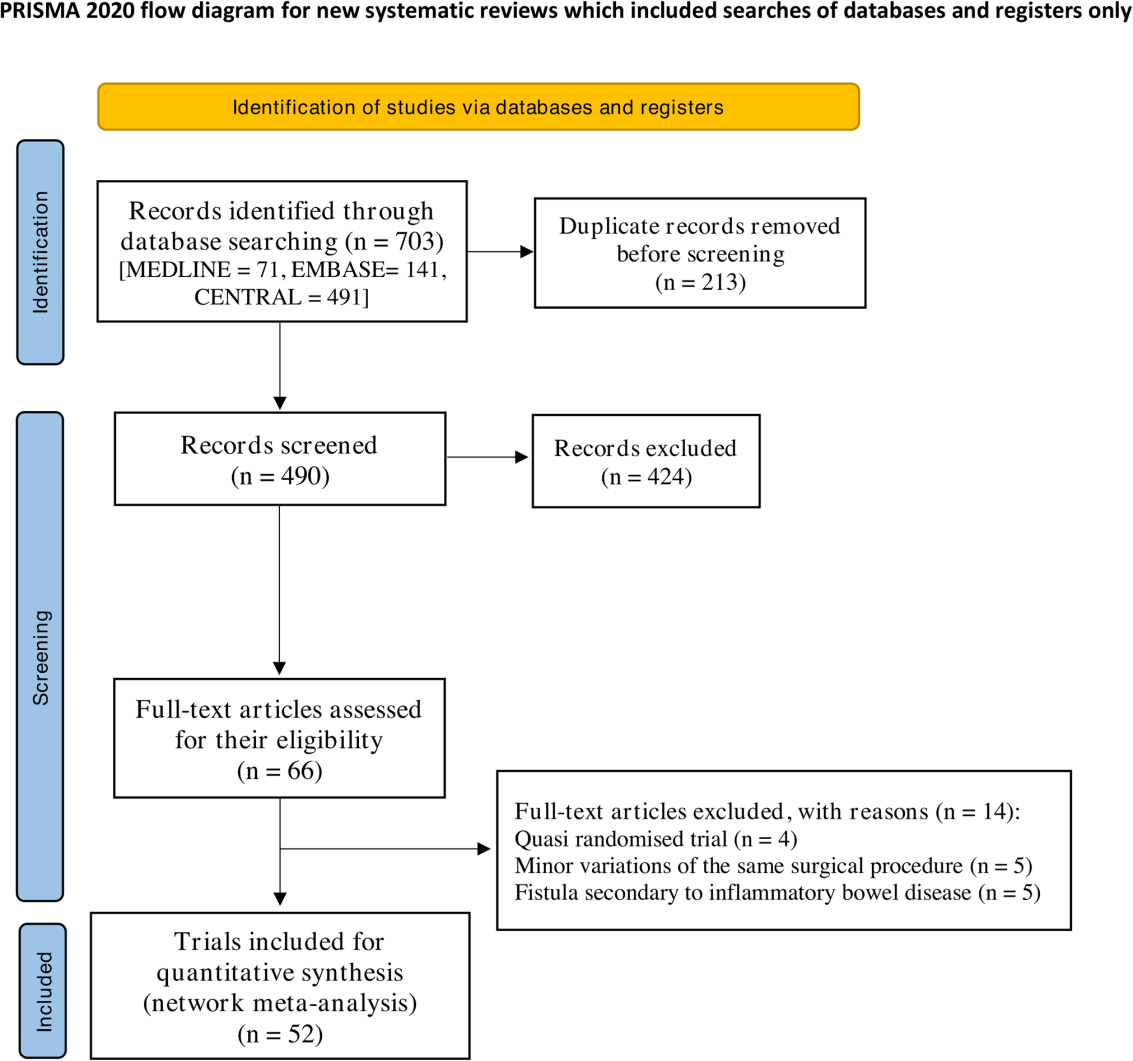
Preferred Reporting Items for Systematic Reviews and Meta-Analyses (PRISMA) flow diagram highlighting the selection process for eligible randomised controlled trials

Database searches identified 703 records, from which 52 RCTs were included (Fig. 1 and Supplementary Appendix S3). Five RCTs were excluded on the basis of their inclusion of patients with anal fistula secondary to IBD [47–51].

### Study characteristics

Characteristics of the included studies are detailed in Table 1. The highest proportion of trials were conducted in China (19%, 10/52), followed by Egypt (13%, 7/52) and Spain (12%, 6/52). There were eight multicentre RCTs, including a median of 6 (IQR 5–16) hospitals; it was unclear how many hospitals were included in one study [52].

**Table 1:** Summary characteristics of included randomised controlled trials

First author (year)	Geographic location(s)	No. of centres	Anal fistula classification	Treatment	No. of patients^a^
A ba-bai-ke-re (2010) [83]	China	1	Complex	Collagen plug	45 (45)
				Advancement flap	45 (45)
A ba-bai-ke-re (2012) [53]	China	1	Complex	Collagen plug with seton	20 (20)
				Seton	20 (20)
Al Sebai (2021) [92]	Egypt	1	Simple	LIFT	15 (15)
				Fistulotomy	15 (15)
Altomare (2011) [54]	Italy	7	Complex	Collagen plug	39 (38)
				Seton	25 (24)
Anan (2019) [93]	Egypt	1	Simple	Fistulotomy with marsupialisation	31 (30)
				Fistulotomy	31 (30)
Bondi (2017) [55]	Norway, Sweden	3	Complex	Collagen plug	48 (41)
				Advancement flap	46 (40)
Chalya (2013) [94]	Tanzania	1	Simple	Fistulotomy with marsupialisation	80 (80)
				Fistulotomy	82 (82)
Chen (2005) [29]	China	1	Complex	Seton	96 (96)
				Fistulectomy	96 (96)
Cwalinski (2021) [56]	Poland	1	Complex	Topical platelet-rich plasma following fistula drainage	10 (10)
				Topical platelet-rich plasma	8 (8)
de la Portilla (2019) [57]	Spain	1	Complex	Topical platelet-rich plasma	32 (29)
				Fibrin glue	24 (21)
Dong (2020) [81]	China	1	Simple	LIFT	45 (45)
				Fistulectomy	45 (45)
Ellis (2006) [58]	USA	1	Complex	Advancement flap with collagen plug	28 (28)
				Advancement flap	30 (30)
Elshamy (2022) [101]	Egypt	1	Complex	LIFT	25 (22)
				Modified fistulotomy	25 (21)
				Seton	25 (23)
Filingeri (2004) [59]	Italy	1	Simple	Radiofrequency fistulectomy	11 (10)
				Fistulotomy	11 (10)
Garcia-Arranz (2020) [84]	Spain	5	Complex	Adipose-derived stem cells with collagen plug	23 (20)
				Fibrin glue	21 (19)
Garcia-Olmo (2009) [85]	Spain	1	Complex	Adipose-derived stem cells with collagen plug	24 (24)
				Fibrin glue	25 (25)
Goudar (2020) [60]	India	1	Simple	LIFT	30 (30)
				Fistulectomy	30 (30)
Gupta (2003) [61]	India	1	Simple	Radiofrequency fistulotomy	50 (50)
				Fistulotomy	50 (50)
Hammond (2009) [86]	UK	1	Complex	Collagen plug	13 (13)
				Fibrin glue	16 (15)
Han (2016) [62]	China	5	Complex	LIFT with collagen plug	119 (117)
				LIFT	120 (118)
Hermann (2022) [63]	Poland	1	Complex	Topical platelet-rich plasma	49 (49)
				Advancement flap	47 (47)
Herreros (2012) [64]	Spain	19	Complex	Adipose-derived stem cells with fibrin glue	66 (60)
				Adipose-derived stem cells	68 (64)
				Fibrin glue	66 (59)
Ho (1998) [95]	Singapore	1	Simple	Fistulotomy with marsupialisation	51 (51)
				Fistulotomy	52 (52)
Ho (2001) [65]	Singapore	1	Simple	Seton	46 (46)
				Fistulotomy	54 (54)
Ho (2005) [52]	Singapore	NS	Simple	Advancement flap	10 (10)
				Fistulotomy	10 (10)
Jain (2012) [96]	India	1	Simple	Fistulotomy with marsupialisation	20 (20)
				Fistulectomy	20 (20)
Kalim (2017) [66]	Pakistan	1	Simple	Fistulectomy	152 (152)
				Fistulotomy	152 (152)
Khoshnevis (2022) [79]	Iran	1	Complex	“JUMP” technique^b^	65 (65)
				Seton	65 (65)
Kronborg (1985) [97]	Denmark	1	Simple	Fistulotomy	26 (26)
				Fistulectomy	21 (21)
Kumar (2022) [87]	India	1	Complex	Advancement flap	42 (42)
				LIFT	42 (42)
Madbouly (2014) [91]	Egypt	1	Complex	LIFT	35 (35)
				Advancement flap	35 (35)
Madbouly (2021) [67]	Egypt	1	Complex	LIFT with topical platelet-rich plasma	49 (49)
				LIFT	49 (49)
Mascagni (2018) [68]	Italy	1	Simple	Incision-thread drawing	15 (15)
				Fistulectomy with primary sphincter reconstruction	15 (15)
Mushaya (2012) [88]	Australia	1	Complex	LIFT	25 (25)
				Advancement flap	14 (13)
Nazeer (2012) [98]	Pakistan	1	Simple	Fistulectomy	75 (75)
				Fistulotomy	75 (75)
Nour (2020) [100]	Egypt	1	Simple	Fistulotomy with marsupialisation	35 (35)
				Fistulotomy	35 (35)
Ortiz (2009) [80]	Spain	1	Complex	Collagen plug	16 (15)
				Advancement flap	16 (16)
Perez (2006) [69]	Spain	1	Complex	Advancement flap	27 (27)
				Fistulotomy with primary sphincter reconstruction	28 (28)
Pescatori (2006) [70]	Italy	1	Complex	Fistulotomy with marsupialisation	22 (22)
				Fistulectomy	24 (24)
Rezk (2022) [71]	Egypt	1	Simple	LIFT with adipose-derived stem cells	35 (35)
				LIFT	35 (35)
Sahakitrungruang (2011) [72]	Thailand	1	Simple	Fistulotomy with marsupialisation	25 (25)
				Fistulotomy	25 (25)
Schwandner (2018) [89]	Germany	6	Complex	Collagen plug	43 (33)
				Advancement flap	39 (33)
Singer (2005) [73]	USA	16	Complex	Fibrin glue with antibiotics	24 (24)
				Fibrin glue with fistula closure surgery	25 (25)
				Fibrin glue with antibiotics followed by fistula closure surgery	26 (26)
Sørensen (2021) [74]	Denmark	1	Complex	Fistulectomy with primary sphincter reconstruction	22 (22)
				Video-assisted anal fistula treatment	23 (23)
van der Hagen (2011) [90]	Netherlands	1	Complex	Fibrin glue	15 (15)
				Advancement flap	15 (15)
van Koperen (2011) [75]	Netherlands	6	Simple	Collagen plug	31 (31)
				Advancement flap	29 (29)
Vinay (2017) [99]	India	1	Simple	LIFT	25 (25)
				Fistulotomy	25 (25)
Wang (2012) [30]	China	1	Complex	Suture dragging with pad compression	30 (30)
				Fistulotomy	30 (30)
Wang (2021) [76]	China	1	Simple	Modified fistulotomy	63 (63)
				Fistulotomy	62 (62)
Wu (2021) [33]	China	1	Simple	Video-assisted modified LIFT	37 (37)
				Incision-thread drawing	30 (30)
Yan (2020) [77]	China	1	Complex	Fistulectomy with seton	40 (40)
				Seton	40 (40)
Zhang (2020) [78]	China	1	Complex	Video-assisted anal fistula treatment	37 (37)
				Fistulotomy with seton	38 (38)

*LIFT* ligation of the inter-sphincteric fistula tract, *NS* not stated

^a^Parenthesis indicate patients who were randomised and received each treatment (i.e. those who were included in the final analyses)

^b^Inversion of the fistula tract

### Patient characteristics and treatments comparisons

A total of 4157 patients were randomised, of which 4069 participants were included in the final analyses (1799 with simple and 2270 with complex anal fistula; Table 1). There was substantial heterogeneity in anal fistula definitions across the included trials (Supplementary Appendix S4).

Overall, 33 different surgical treatments were analysed (Table 1). Thirteen different treatments were trialled in the 22 studies including patients with simple anal fistula, while 28 different treatments were analysed in the 30 studies of patients with complex anal fistula.

### Quality assessment

The majority of RCTs were assessed as being at high ROB (69.2%, 36/52 studies; Supplementary Fig. S1). This predominantly resulted from biases in how outcomes were measured (63.5%, 33/52) or due to the lack of blinding among patients and/or surgeons (53.8%, 28/52); although in two of these studies, ‘moderate’ rather than high ROB was assigned as outcome assessors remained blinded [79, 80]. Detection bias was low as there were no missing outcome data in all but one study (98.1%, 51/52) [30]. Quality assessments for individual trials based on each of the five ROB domains are reported in Supplementary Fig. S2.

### Transitivity analysis

Demographic characteristics (age and sex) of patients randomised to each treatment varied considerably (Supplementary Appendix S5, A & B), with the mean age of patients ranging from 30.4 to 53.1 years old, and proportion of female patients ranging from 0 to 60%. Substantial geographical diversity among the different surgical treatments assessed was also observed (Supplementary Appendix S5, C), with the novel treatments (i.e. which were employed in only a single trial) mostly originating from China, India, Poland, Spain, and Egypt.

### Primary outcomes

#### Short-term success (≤ 6 months after surgery)

**Fig. 2:**
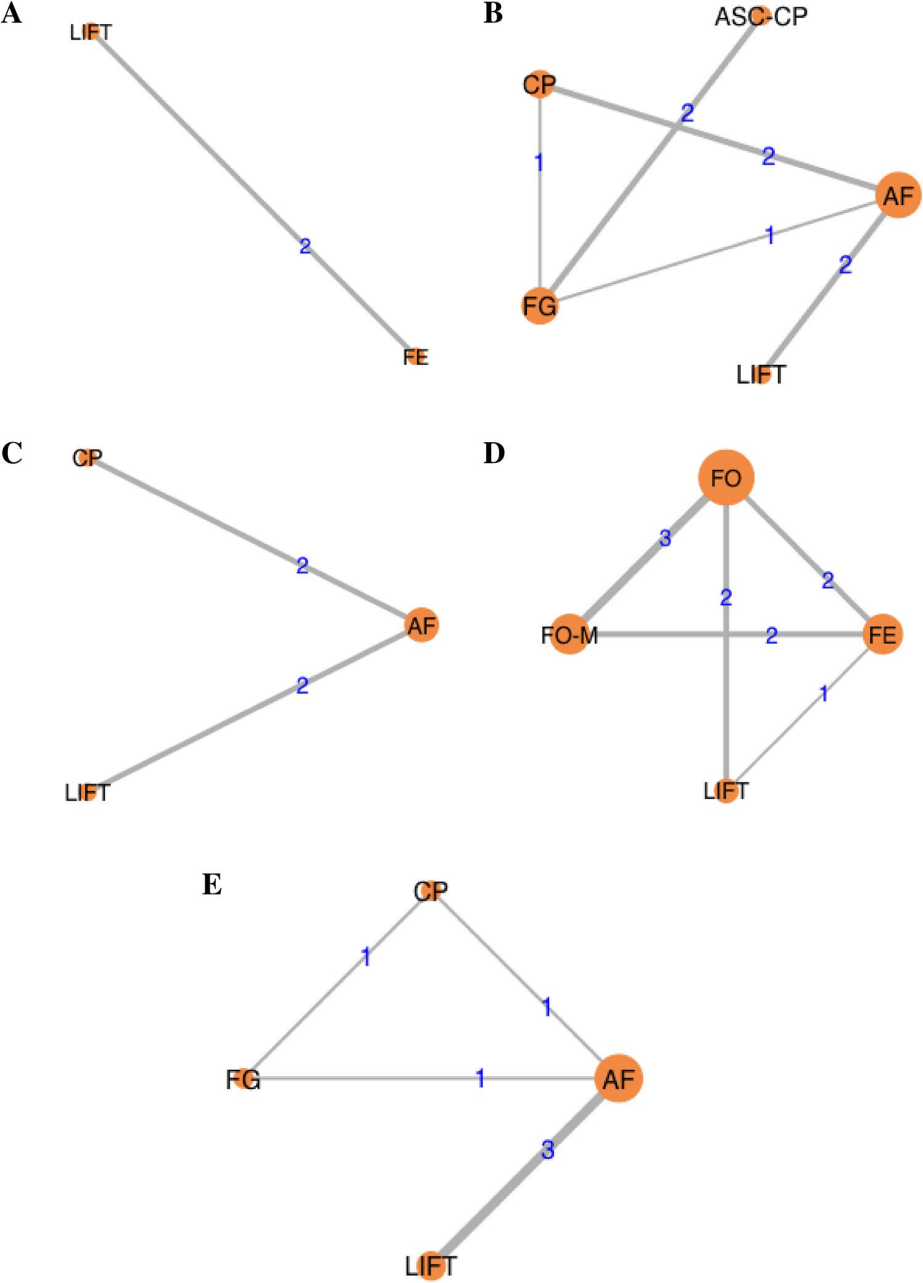
Network plots depicting all direct comparisons between different treatments for the following outcomes: short-term success (≤ 6 months after surgery) in patients with **a** simple and **b** complex anal fistula, **c** long-term success (> 6 months after surgery) in patients with complex anal fistula, and bowel incontinence among patients with **d** simple and **e** complex anal fistula (nodes correlated with the number of patients receiving each treatment, while the thickness of each line connecting two nodes was proportional to the number of trials in which each treatment was assessed. (AF, advancement flap; ASC-CP, adipose-derived stem cells combined with a collagen plug; CP, collagen plug; FE, fistulectomy; FG, fibrin glue; FO, fistulotomy; FO-M, fistulotomy with marsupialisation; LIFT, ligation of the inter-sphincteric fistula tract)

**Fig. 3:**
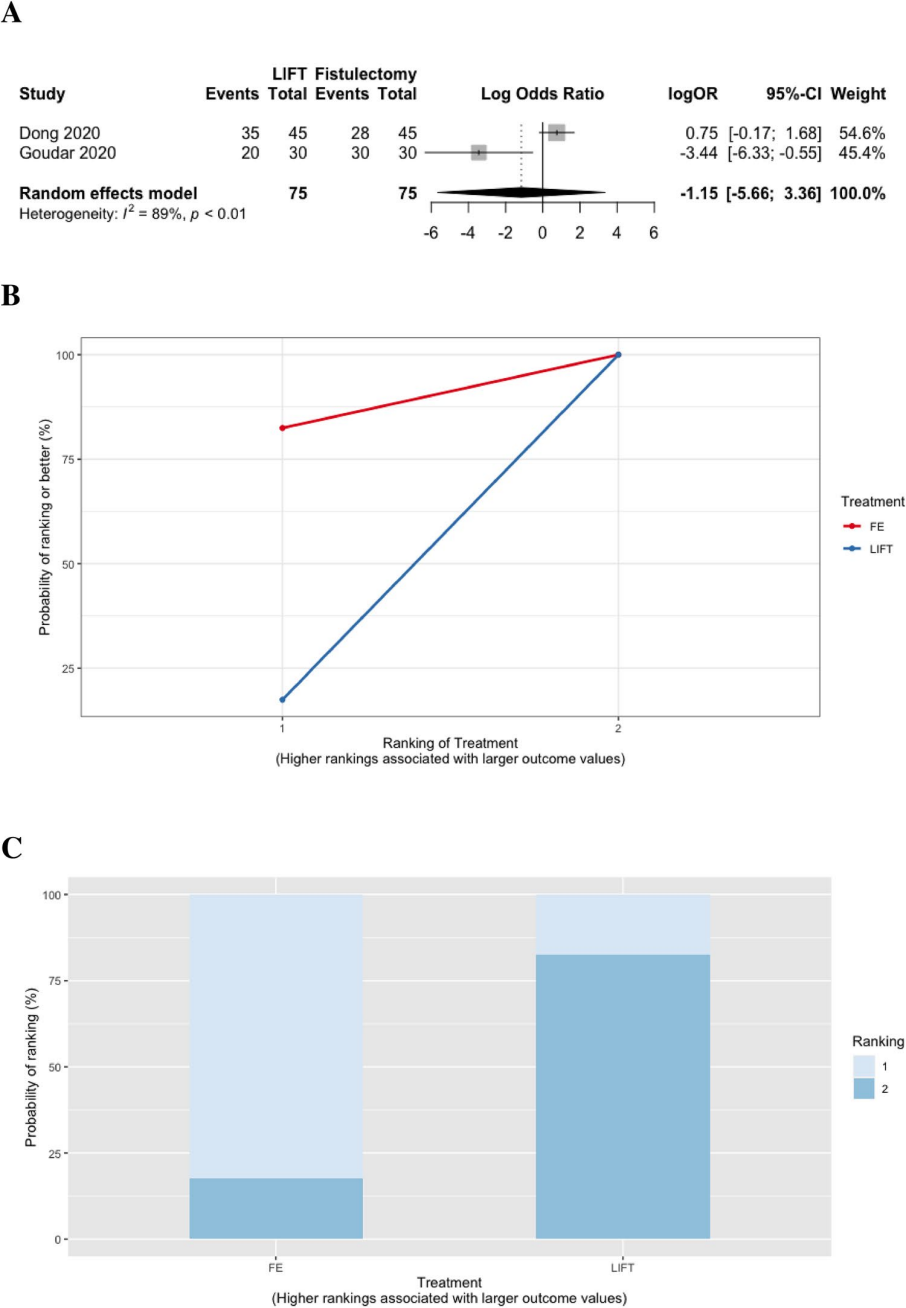
Short-term success rates (≤ 6 months after surgery) comparing treatments in patients with simple anal fistula demonstrated by **a** forest plot (LIFT vs. fistulectomy), **b** SUCRA curve of relative ranking probabilities, and **c** rankogram plot. (FE, fistulectomy; LIFT, ligation of the inter-sphincteric fistula tract)

Success rates in the short term were reported in two studies of 150 participants with simple anal fistula, in which two treatments were compared: ligation of the inter-sphincteric fistula tract (LIFT) versus fistulectomy (Fig. 2a) [81, 82]. There were no significant differences in rates of success between LIFT and fistulectomy (log OR − 1.2, 95% CrI − 5.7 to 3.4; Fig. 3a–c).

**Fig. 4:**
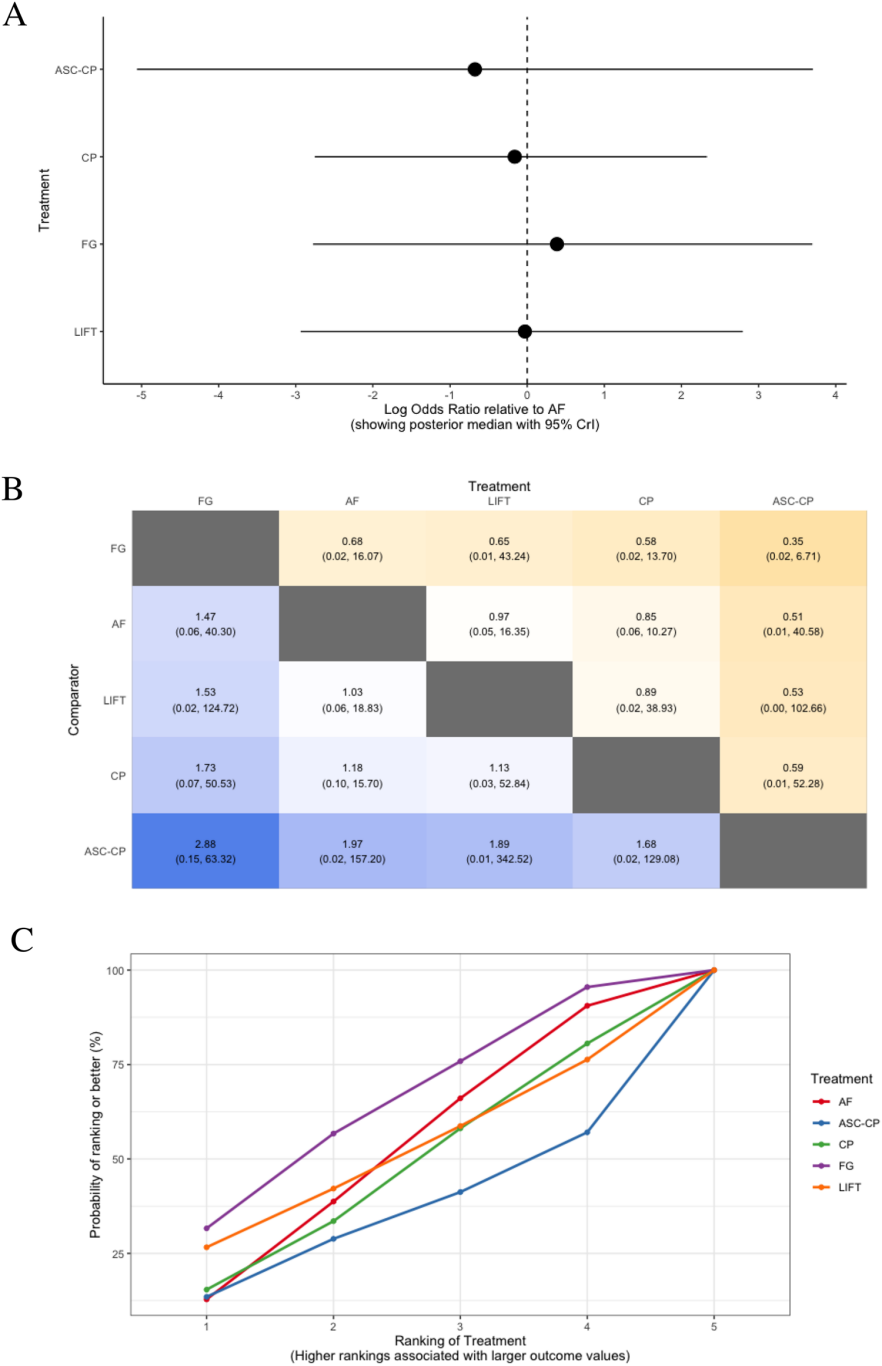
Different treatment comparisons for short-term healing rates (≤ 6 months after surgery) in patients with complex anal fistula, shown via **a** forest plot (relative to advancement flap), **b** heat plot, **c** SUCRA curve, and **d** rankogram plot (AF, advancement flap; ASC-CP, adipose-derived stem cells combined with a collagen plug; CP, collagen plug; FG, fibrin glue; LIFT, ligation of the inter-sphincteric fistula tract)

Short-term success of complex anal fistulae was evaluated in eight trials, consisting of 424 participants, in which five different treatments were assessed: collagen plug, advancement flap, LIFT, fibrin glue, and adipose-derived stem cells combined with a collagen plug (Fig. 2b) [84–91]. Rates of success of anal fistula healing were not significantly different between any of the treatments (Fig. 4a–d).

#### Long-term success (> 6 months after surgery)

No trials described long-term success rates for different treatments among participants with simple anal fistulae.

**Fig. 5:**
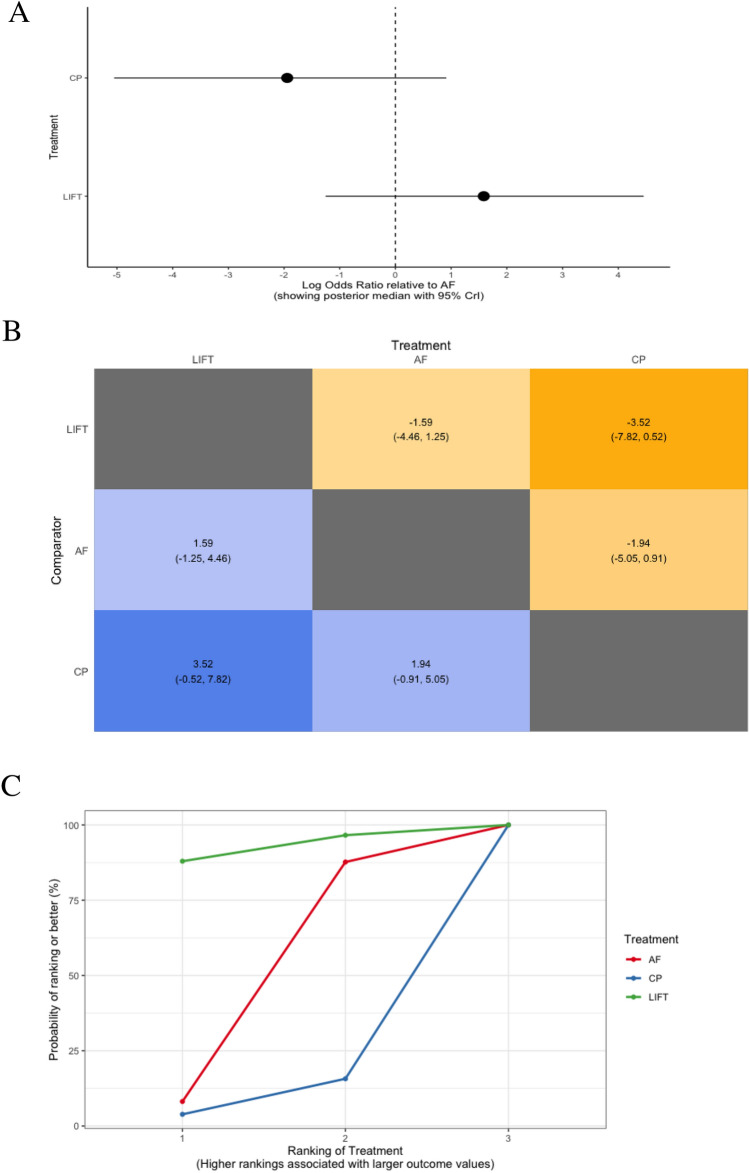
Long-term success rates (> 6 months after surgery) between different treatments in patients with complex anal fistula illustrated using **a** forest plot (relative to advancement flap), **b** heat plot, **c** SUCRA curve, and **d** rankogram plot (AF, advancement flap; CP, collagen plug; LIFT, ligation of the inter-sphincteric fistula tract)

Four trials assessed rates of success in the long-term among 251 participants with complex anal fistula, in which three different treatments were compared: LIFT, advancement flap, and collagen plug (Fig. 2c) [80, 87, 89, 91]. On the available evidence, these three treatments did not differ from one another in terms of their efficacy for achieving long-term success regarding anal fistula healing (Fig. 5a, b). LIFT ranked as the best performing treatment in 92.3% of comparisons (*n* = 2 trials with 77 participants) (Table 3, Fig. 5c–d).

#### Bowel incontinence

**Fig. 6:**
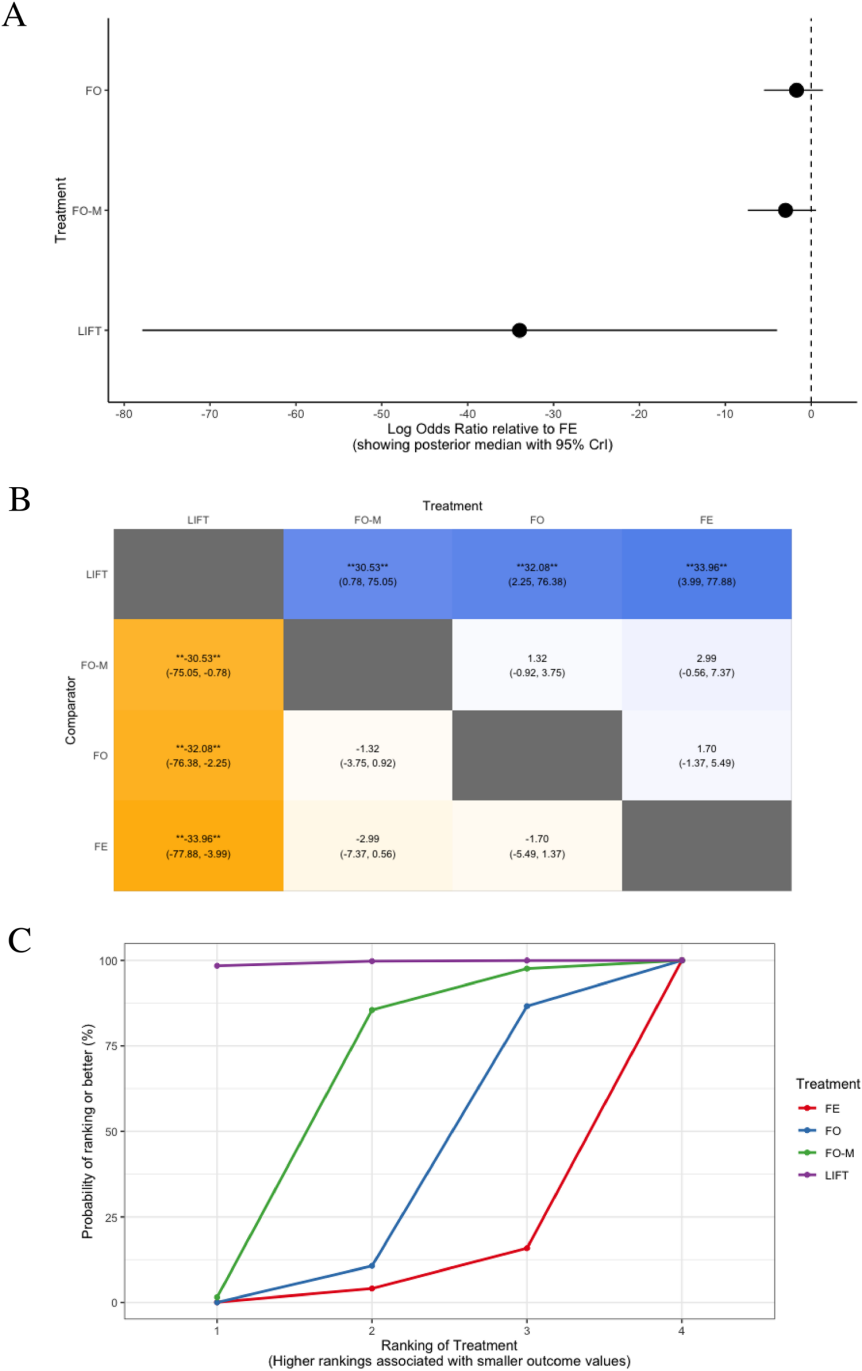
Comparisons between different treatments for minimising bowel incontinence in patients with simple anal fistula demonstrated using **a** forest plot (relative to fistulectomy), **b** heat plot, **c** SUCRA curve, and **d** rankogram plot (FE, fistulectomy; FO, fistulotomy; FO-M fistulotomy with marsupialisation; LIFT, ligation of the intersphincteric fistula tract)

Rates of bowel incontinence were evaluated in 10 trials, including 772 participants with simple anal fistula, and comparing four different treatments: LIFT, fistulotomy with marsupialisation, fistulotomy, and fistulectomy (Fig. 2d) [82, 92–100]. LIFT resulted in significantly lower rates of bowel incontinence compared to fistulotomy with marsupialisation (log OR − 30.5, 95% CrI − 75.1 to − 0.8), fistulotomy (log OR − 32.1, 95% CrI − 76.4 to − 2.3), and fistulectomy (log OR − 34.0, 95% CrI − 77.9 to − 4.0) (Fig. 6a, b). Of these treatments, LIFT ranked best for minimising rates of bowel incontinence (in 99.1% of comparisons; *n* = 3 trials with 70 participants), while fistulectomy was the worst performing treatment and ranked best in only 6.8% of comparisons (*n* = 5 trials with 228 participants; Table 2, Fig. 6c, d).

**Table 2:** SUCRA ranking probabilities among treatment comparisons for short-term success rates and bowel incontinence in patients with simple anal fistula

Treatment	No. of trials	Total participants	SUCRA value (rank)
Short-term success (≤ 6 months after surgery)^a^			
Fistulectomy	2	75	82.5 (1)
LIFT	2	75	17.5 (2)
Bowel incontinence^b^			
LIFT	3	70	99.1 (1)
Fistulotomy with marsupialisation	5	216	61.5 (2)
Fistulotomy	7	258	32.6 (3)
Fistulectomy	5	228	6.8 (4)

*LIFT* ligation of the inter-sphincteric fistula tract, *SUCRA* surface under the cumulative ranking

^a^Complete healing of the anal fistula without recurrence or persistence of symptoms on follow-up. Healing was defined on the basis of clinical examination, and/or endoanal ultrasound scan (USS) or pelvic magnetic resonance imaging (MRI) findings, or was self-reported by patients on the basis of the resolution of symptoms at follow-up

^b^Defined as incontinence to either gas, liquid, and/or solid stool

**Fig. 7:**
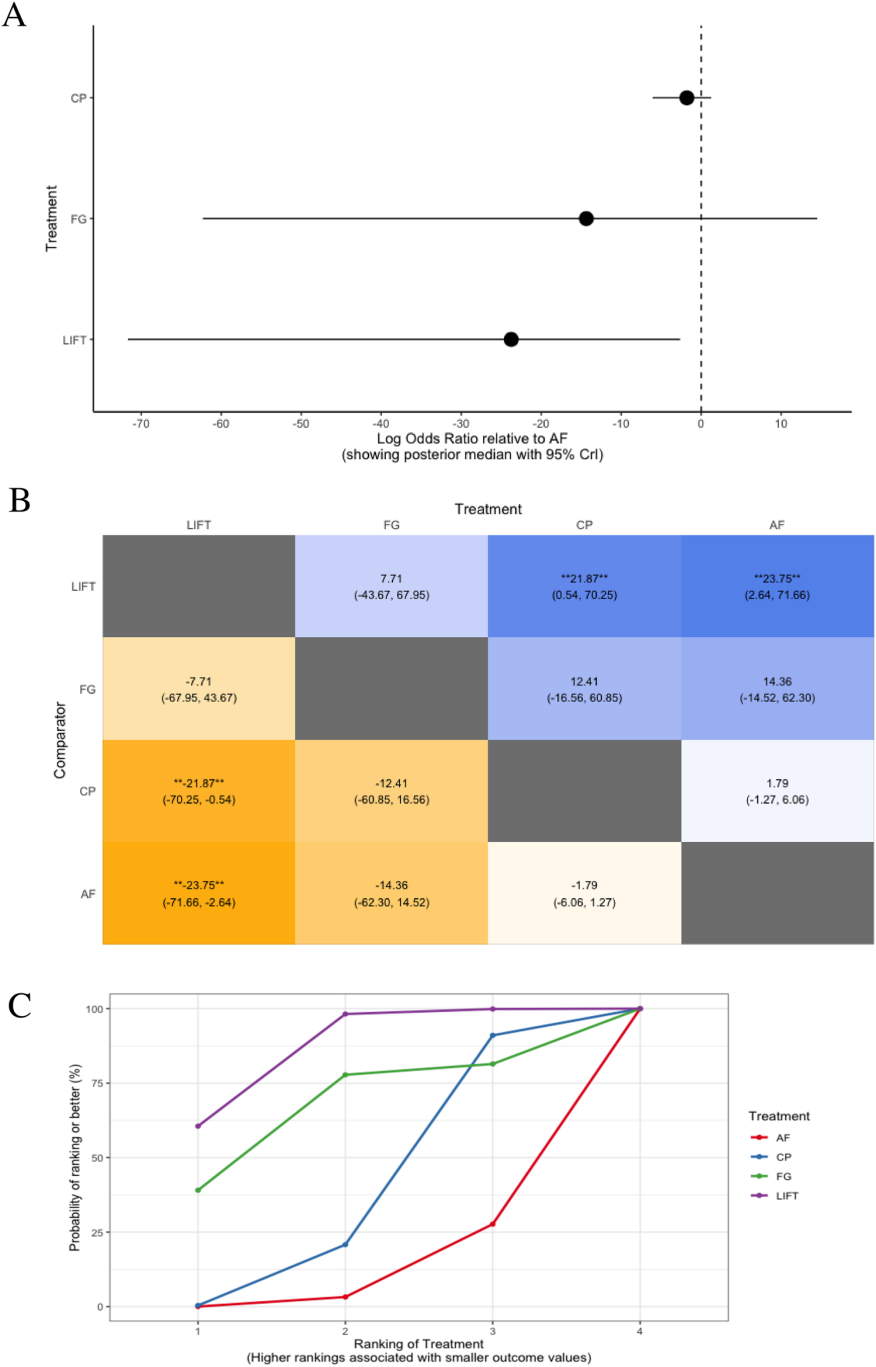
Treatment comparisons for minimising bowel incontinence in patients with complex anal fistula using **a** forest plot (relative to advancement flap), **b** heat plot, **c** SUCRA curve, and **d** rankogram plot (AF, advancement flap; CP, collagen plug; FG, fibrin glue; LIFT, ligation of the inter-sphincteric fistula tract)

In the setting of complex anal fistula, bowel incontinence was analysed in six trials, comparing four treatments (LIFT, fibrin glue, collagen plug, and advancement flap) among 340 participants (Fig. 2e) [83, 86–88, 90, 91]. LIFT was associated with significantly lower rates of bowel incontinence compared with collagen plug (log OR − 21.9, 95% CrI − 70.3 to − 0.5) and advancement flap (log OR − 23.8, 95% CrI − 71.7 to − 2.6), but not relative to fibrin glue (log OR − 7.7, 95% CrI − 68.0 to 43.7) (Fig. 7a, b). Among the treatments assessed, LIFT ranked the best (in 86.2% of comparisons; *n* = 3 trials with 102 participants), while advancement flap was the worst performing treatment, ranking best in only 10.3% of comparisons (*n* = 5 trials with 150 participants) (Table 3, Fig. 7c, d).

**Table 3:** SUCRA ranking probabilities among treatment comparisons for short and long-term success rates and bowel incontinence in patients with complex anal fistula

Treatment	No. of trials	Total participants	SUCRA value (rank)
Short-term success (≤ 6 months after surgery)^a^			
Fibrin glue	4	74	62.7 (1)
LIFT	2	67	51.3 (2)
Advancement flap	5	148	50.8 (3)
Collagen plug	3	91	46.2 (4)
Adipose-derived stem cells with a collagen plug	2	44	39.0 (5)
Long-term success (> 6 months after surgery)^a^			
LIFT	2	77	92.3 (1)
Advancement flap	4	126	47.9 (2)
Collagen plug	2	48	9.8 (3)
Bowel incontinence^b^			
LIFT	3	102	86.2 (1)
Fibrin glue	2	30	66.1 (2)
Collagen plug	2	58	37.4 (3)
Advancement flap	5	150	10.3 (4)

*LIFT* ligation of the inter-sphincteric fistula tract, *SUCRA* surface under the cumulative ranking

^a^Complete healing of the anal fistula without recurrence or persistence of symptoms on follow-up. Healing was defined on the basis of clinical examination, and/or endoanal ultrasound scan (USS) or pelvic magnetic resonance imaging (MRI) findings, or was self-reported by patients based on the resolution of symptoms at follow-up

^b^Defined as incontinence to either gas, liquid, and/or solid stool

### Secondary outcomes

#### Hospital length of stay

Two studies assessed hospital length of stay, comparing two treatments (fistulotomy with marsupialisation and fistulotomy) among 263 participants with simple anal fistula (Supplementary Appendix S6) [94, 95]. The duration of hospitalisation did not differ between fistulotomy with marsupialisation versus fistulotomy (MD − 0.4, 95% CrI − 1.6 to 0.9) (Supplementary Appendix S6 and Table S7).

Hospital LOS could not be evaluated in participants with complex anal fistula as no single treatment was evaluated in more than one trial (Table S8).

#### Overall postoperative complications

Two studies evaluated overall postoperative complication rates, comparing two different treatments (fistulotomy with marsupialisation versus fistulotomy) in 163 participants with simple anal fistula (Supplementary Appendix S6) [93, 95]. Postoperative complications rates were similar between fistulotomy with marsupialisation and fistulotomy (log OR − 0.7, 95% CrI − 2.2 to 0.7) (Supplementary Appendix S6 and Table S9).

Overall postoperative complications were also assessed in two studies, comparing two treatments (LIFT versus advancement flap) in 108 participants with complex anal fistula (Supplementary Appendix S6) [88, 91]. Complication rates were not significantly different between LIFT versus advancement flap (log OR − 0.5, 95% CrI − 1.9 to 0.9) (Supplementary Appendix S6 and Table S10).

### Sensitivity analysis

Results of the sensitivity analyses, when all treatments were assessed, are reported in Supplementary Appendix S6, with narrative summaries provided in Supplementary Appendix S7. Findings were concordant with the overall analysis. LIFT consistently ranked first in terms of minimising bowel incontinence for both simple and complex anal fistula. For patients with simple anal fistula, LIFT resulted in significantly lower rates of bowel incontinence compared with fistulotomy, fistulectomy, and seton. With respect to complex anal fistula, LIFT was associated with less bowel incontinence compared to fistulotomy with marsupialisation, fistulectomy, collagen plug, fistulotomy, advancement flap, fistulotomy with primary sphincter reconstruction, and suture dragging with pad compression. Success rates in the short and long term, hospital LOS, and overall postoperative complication rates were not significantly different between any of the treatments for both simple and complex anal fistula.

### Inconsistency and heterogeneity analysis

On visual inspection of the consistency models, systematic inconsistencies between indirect and direct treatment effects within the network were observed for short-term success in patients with complex anal fistulae, and for overall complication rates in patients with both simple and complex anal fistulae (Supplementary Appendix S8). Results of the heterogeneity analysis are reported in Supplementary Appendix S9. Variability in effect sizes between trials was largely attributable to true heterogeneity rather than chance with respect to both short-term and long-term success. For short-term success, such variability was observed between studies comparing LIFT versus fistulectomy (*I*^2^ = 75%) [82, 83] in patients with simple anal fistula, and LIFT versus advancement flap (*I*^2^ = 82%) [87, 88] in patients with complex anal fistula. For long-term success in patients with complex anal fistulae, studies comparing advancement flap with collagen plug also demonstrated variability in their effect sizes that was mainly due to true heterogeneity rather than chance (*I*^2^ = 86%) [80, 89].

## Discussion

This study compared the relative efficacy of various surgical treatments for optimising healing and minimising bowel incontinence among 52 RCTs including patients with simple or complex anal fistula. A large number of treatments were identified, although many of the novel treatments were excluded from the primary analyses as they were studied in only a single trial, including a small number of patients. Of the commonly studied treatments, LIFT was associated with the least impact on bowel continence, irrespective of anal fistula classification and despite the sensitivity analysis in which all treatments were included. There were no differences between treatments for short-term success rates (6 months or less from surgery), hospital LOS and overall postoperative complications in patients with simple and complex anal fistulae. Treatment efficacy for achieving long-term success (more than 6 months after surgery) were also similar for complex anal fistula, whilst long-term success could not be evaluated because of the lack of trials evaluating this outcome in patients with simple anal fistula.

Two previous NMAs have also assessed the optimum treatment for preserving anal sphincter function, specifically for patients with complex anal fistula [102, 103]. The more recent of these reviews concluded that the “TROPIS” procedure achieves the best rate of healing, whilst an improved LIFT (“imLIFT”) technique was associated with the lowest incidence of complications [102]. However, the reliability of these conclusions may be limited by the inclusion of evidence derived from non-randomised (cohort) studies, which introduces selection and confounding bias. Furthermore, novel treatments such as the imLIFT technique, which were studied in only a single RCT with a small number of patients, were also included in their NMA. Effect sizes for these treatments were obtained mostly from statistically derived indirect comparisons because of the scarcity of direct evidence from head-to-head trials, and were associated with high levels of imprecision resulting from underpowered analyses [25]. The latter study was conducted in 2017, and pooled evidence presented in 20 RCTs which included patients with only complex anal fistula [103]. However, 21 trials evaluating the efficacy of different treatments in patients with anal fistula have been published since then. The current NMA utilised these latest data presented in all 52 relevant RCTs, in addition to analysing the efficacy of different treatments among patients with both simple and complex anal fistula, highlighting its strength. Moreover, to minimise bias, the primary analyses included only treatments that were connected to at least two other treatments in the network (i.e. treatments that were evaluated in more than one trial), thus facilitating a more robust NMA.

LIFT is primarily indicated for trans-sphincteric fistulae, where the fistula tract passes through the inter-sphincteric space [19, 104–106]. Horseshoe fistula and those secondary to Crohn’s disease have been identified as significant predictors for failure after LIFT [19]. The anatomical curvature of horseshoe fistula tracts mean it is more challenging to achieve complete eradication, as the curved extent of the track in the deep post-anal space may not be adequately drained, providing a nidus for cryptoglandular sepsis to recur [19]. Perianal fistulae secondary to Crohn’s disease are associated with an increased incidence of irregular fistula tracks, including those that are curved and/or contain multiple external openings [107, 108]. For this reason, studies which included patients with anal fistula deemed secondary to IBD were excluded from the present review. Appropriate patient selection for LIFT is therefore paramount, although current data lacks granularity to accurately differentiate outcomes based on fistula location. Nonetheless, the NMA has shown that LIFT appears to have the least impact on bowel continence amongst patients with all types of cryptoglandular anal fistula.

In this review, treatment efficacy was evaluated for simple and complex fistula separately. However, there was significant heterogeneity in terms of how anal fistulae were defined by individual authors across the included trials. Fistulae were commonly classified on the basis of clinical impression following a digital rectal examination, although proctoscopy, endoanal USS and pelvic MRI were also utilised in many cases. With respect to this observed variability, there remains a need to reach a consensus on an appropriate system for classifying anal fistula. This issue was not addressed in the most recent Association of Coloproctology of Great Britain and Ireland (ACPGBI) position statement on anal fistula management [8], although the European Society of Coloproctology (ESCP) are currently working to develop a definition consensus [109]. This will facilitate greater comparability of treatment efficacy between studies, and aid surgeons in deciding on the most suitable treatment in view of the anatomical course and complexity of a patient’s anal fistula.

There are some limitations to this study. Our primary analysis included only treatments which were assessed in multiple (i.e. more than one) RCTs. However, this was determined a priori, to improve statistical rigor relative to previous NMA estimates in this field [102, 103]. The inherent consequence of this was the inability to compare more sparsely studied interventions for managing simple and complex anal fistula. However, the results of our sensitivity analyses, when all treatments were included, were concordant with our overall results. In the future, these novel procedures need further study in larger trials, to better elucidate their comparative efficacy in managing simple and complex anal fistula. While the focus of this review was primarily on ascertaining the anal fistula treatment which best balances success in achieving anal fistula healing with preservation of anal sphincter continence, this proved difficult owing to variability in how these outcomes were measured (Supplementary Appendix S10), and in the timing of follow-up (Supplementary Appendix S11). Healing was either subjectively recorded (in 63.5% of studies, 33/52), or was based on objective clinical criteria (i.e. physical examination or pelvic MRI findings) but were recorded by investigators who were not blinded to the treatment received. Distinguishing between the different severities of bowel incontinence (i.e. those who developed incontinence to gas versus liquid versus solid stool) was also not possible because of substantial ambiguity in how these data were reported. Future trials should therefore consider consistently reporting on a core set of outcomes using standardised definitions [110, 111], so that surgeons may accurately educate and counsel patients on the relative risks and efficacy of each treatment option for anal fistula. Additionally, in contrast to the two previous NMAs on this topic [102, 103], to attempt to mitigate the inconsistencies in follow-up duration between trials, the efficacy of each treatment for achieving healing was measured at two time points (6 months or less and more than 6 months after surgery). Despite this, the results for long-term efficacy of different treatments should be interpreted with caution given the limited long-term follow-up data that are available in existing RCTs, highlighting a need for future studies to collect higher quality data over a longer period of time following anal fistula surgery.

## Conclusions

On the basis of existing RCT data, there is insufficient evidence to recommend one treatment over another regarding their short and long-term efficacy in successfully facilitating healing of both simple and complex anal fistula. However, LIFT appears to be associated with the least impairment of bowel continence irrespective of anal fistula classification. The generalisability of these findings may be limited by the lack of standardised preoperative investigations and classification systems for anal fistula, variability in the reporting of healing and incontinence outcomes and their definitions, together with the fact that some novel techniques were studied in only a small number of patients with short durations of follow-up, and in select geographical areas.

## Supplementary Information

Below is the link to the electronic supplementary material.

Supplementary file1 (DOCX 6035 KB)

## Data Availability

The data that support the findings of this study are available from the corresponding author upon reasonable request.

## References

[1] Seow-Choen F, Nicholls RJ (1992). Anal fistula. Br J Surg.

[2] Steele SR, Kumar R, Feingold DL, Rafferty JL, Buie WD (2011). Standards practice task force of the american society of colon and rectal surgeons. Practice parameters for the management of perianal abscess and fistula-in-ano. Dis Colon Rectum.

[3] Felt-Bersma RJF, Bartelsman JF (2009). Haemorrhoids, rectal prolapse, anal fissure, peri-anal fistulae and sexually transmitted diseases. Best Pract Res Clin Gastroenterol.

[4] Emile SH, Elfeki H, El-Said M, Khafagy W, Shalaby M (2021). Modification of parks classification of cryptoglandular anal fistula. Dis Colon Rectum.

[5] Parks AG, Gordon PH, Hardcastle JD (1976). A classification of fistula-in-ano. Br J Surg.

[6] Vogel JD, Johnson EK, Morris AM (2016). Clinical practice guideline for the management of anorectal abscess, fistula-in-ano, and rectovaginal fistula. Dis Colon Rectum.

[7] Fazio VW (1987). Complex anal fistulae. Gastroenterol Clin North Am.

[8] Williams G, Williams A, Tozer P (2018). The treatment of anal fistula: second ACPGBI position statement-2018. Colorectal Dis.

[9] Cavanaugh M, Hyman N, Osler T (2002). Fecal incontinence severity index after fistulotomy: a predictor of quality of life. Dis Colon Rectum.

[10] Göttgens KWA, Janssen PTJ, Heemskerk J (2015). Long-term outcome of low perianal fistulas treated by fistulotomy: a multicenter study. Int J Colorectal Dis.

[11] Williams JG, MacLeod CA, Rothenberger DA, Goldberg SM (1991). Seton treatment of high anal fistulae. Br J Surg.

[12] Sentovich SM (2003). Fibrin glue for anal fistulas. Dis Colon Rectum.

[13] Garg P, Song J, Bhatia A, Kalia H, Menon GR (2010). The efficacy of anal fistula plug in fistula-in-ano: a systematic review. Colorectal Dis.

[14] Johnson EK, Gaw JU, Armstrong DN (2006). Efficacy of anal fistula plug vs. fibrin glue in closure of anorectal fistulas. Dis Colon Rectum.

[15] Ortíz H, Marzo J (2000). Endorectal flap advancement repair and fistulectomy for high trans-sphincteric and suprasphincteric fistulas. Br J Surg.

[16] Kodner IJ, Mazor A, Shemesh EI, Fry RD, Fleshman JW, Birnbaum EH (1993). Endorectal advancement flap repair of rectovaginal and other complicated anorectal fistulas. Surgery.

[17] Mizrahi N, Wexner SD, Zmora O (2002). Endorectal advancement flap. Dis Colon Rectum.

[18] Bleier JIS, Moloo H, Goldberg SM (2010). Ligation of the intersphincteric fistula tract: an effective new technique for complex fistulas. Dis Colon Rectum.

[19] Emile SH, Khan SM, Adejumo A, Koroye O (2020). Ligation of intersphincteric fistula tract (LIFT) in treatment of anal fistula: an updated systematic review, meta-analysis, and meta-regression of the predictors of failure. Surgery.

[20] Hong KD, Kang S, Kalaskar S, Wexner SD (2014). Ligation of intersphincteric fistula tract (LIFT) to treat anal fistula: systematic review and meta-analysis. Tech Coloproctol.

[21] Elfeki H, Shalaby M, Emile SH, Sakr A, Mikael M, Lundby L (2020). A systematic review and meta-analysis of the safety and efficacy of fistula laser closure. Tech Coloproctol.

[22] Cheng F, Huang Z, Li Z (2020). Efficacy and safety of mesenchymal stem cells in treatment of complex perianal fistulas: a meta-analysis. Stem Cells Int.

[23] Lightner AL, Wang Z, Zubair AC, Dozois EJ (2018). A systematic review and meta-analysis of mesenchymal stem cell injections for the treatment of perianal Crohn’s disease: progress made and future directions. Dis Colon Rectum.

[24] Gutiérrez VM, Guillen SG, Flores MW et al (2021) Safety of allogeneic adipose tissue-derived mesenchymal stem cells for the treatment of complex perianal fistulas not associated with Crohn’s disease: a phase clinical trial. Dis Colon Rectum 64(3):328–334 10.1097/DCR.0000000000001863 33538521

[25] Jansen JP, Fleurence R, Devine B (2011). Interpreting indirect treatment comparisons and network meta-analysis for health-care decision making: report of the ISPOR task force on indirect treatment comparisons good research practices: part 1. Value Health.

[26] Dias S, Caldwell DM (2019). Network meta-analysis explained. Arch Dis Child Fetal Neonatal Ed.

[27] Booth A, Clarke M, Dooley G (2012). The nuts and bolts of PROSPERO: an international prospective register of systematic reviews. Syst Rev.

[28] Hutton B, Salanti G, Caldwell DM (2015). The PRISMA extension statement for reporting of systematic reviews incorporating network meta-analyses of health care interventions: checklist and explanations. Ann Intern Med.

[29] Chen XL, Huang ZH, Zhan YQ (2005). A minimally invasive approach in the treatment of complicated anal fistula through spatium intermuscular of anal sphincter. Zhonghua Wei Chang Wai Ke Za Zhi.

[30] Wang C, Lu JG, Cao YQ, Yao YB, Guo XT, Yin HQ (2012). Traditional Chinese surgical treatment for anal fistulae with secondary tracks and abscess. World J Gastroenterol.

[31] Bramer WM, Giustini D, de Jonge GB, Holland L, Bekhuis T (2016). De-duplication of database search results for systematic reviews in EndNote. J Med Libr Assoc.

[32] Ouzzani M, Hammady H, Fedorowicz Z, Elmagarmid A (2016). Rayyan—a web and mobile app for systematic reviews. Syst Rev.

[33] Wu YF, Zheng BC, Chen Q (2021). Video-assisted modified ligation of the intersphincteric fistula tract, an integration of 2 minimally invasive techniques for the treatment of parks type II anal fistulas. Surg Innov.

[34] Hozo SP, Djulbegovic B, Hozo I (2005). Estimating the mean and variance from the median, range, and the size of a sample. BMC Med Res Methodol.

[35] Wan X, Wang W, Liu J, Tong T (2014). Estimating the sample mean and standard deviation from the sample size, median, range and/or interquartile range. BMC Med Res Methodol.

[36] Higgins JPT, Li T, Deeks JJ (2019). Choosing effect measures and computing estimates of effect. Cochrane handbook for systematic reviews of interventions.

[37] Drevon D, Fursa SR, Malcolm AL (2017). Intercoder reliability and validity of WebPlotDigitizer in extracting graphed data. Behav Modif.

[38] Sterne JAC, Savović J, Page MJ (2019). RoB 2: a revised tool for assessing risk of bias in randomised trials. BMJ.

[39] Sweeting MJ, Sutton AJ, Lambert PC (2004). What to add to nothing? Use and avoidance of continuity corrections in meta-analysis of sparse data. Stat Med.

[40] Chang BH, Hoaglin DC (2017). Meta-analysis of odds ratios: current good practices. Med Care.

[41] Dias S, Sutton AJ, Ades AE, Welton NJ (2013). Evidence synthesis for decision making 2: a generalized linear modeling framework for pairwise and network meta-analysis of randomized controlled trials. Med Decis Making.

[42] Chaimani A, Higgins JPT, Mavridis D, Spyridonos P, Salanti G (2013). Graphical tools for network meta-analysis in STATA. PLoS One.

[43] Mbuagbaw L, Rochwerg B, Jaeschke R (2017). Approaches to interpreting and choosing the best treatments in network meta-analyses. Syst Rev.

[44] Higgins JPT (2003). Measuring inconsistency in meta-analyses. BMJ.

[45] Salanti G (2012). Indirect and mixed-treatment comparison, network, or multiple-treatments meta-analysis: many names, many benefits, many concerns for the next generation evidence synthesis tool. Res Syn Meth.

[46] Dias S (2014) Inconsistency in networks of evidence based on randomised controlled trials. National Institute for Health and Care Excellence (NICE) 27466656

[47] Grimaud JC, Munoz-Bongrand N, Siproudhis L (2010). Fibrin glue is effective healing perianal fistulas in patients with Crohn’s disease. Gastroenterology.

[48] Molendijk I, Bonsing BA, Roelofs H (2015). Allogeneic bone marrow-derived mesenchymal stromal cells promote healing of refractory perianal fistulas in patients with Crohn’s disease. Gastroenterology.

[49] Panés J, García-Olmo D, Van Assche G (2018). Long-term efficacy and safety of stem cell therapy (Cx601) for complex perianal fistulas in patients with Crohn’s disease. Gastroenterology.

[50] Senéjoux A, Siproudhis L, Abramowitz L (2016). Fistula plug in fistulising ano-perineal crohn’s disease: a randomised controlled trial. J Crohns Colitis.

[51] Zhou C, Li M, Zhang Y (2020). Autologous adipose-derived stem cells for the treatment of Crohn’s fistula-in-ano: an open-label, controlled trial. Stem Cell Res Ther.

[52] Ho KS, Ho YH (2005). Controlled, randomized trial of island flap anoplasty for treatment of trans-sphincteric fistula-in-ano: early results. Tech Coloproctol.

[53] A ba-bai-ke-re MMTJ, Wen H, Huang HG, Liang Z, Chu H, Er-ha-ti-hu-sai-yin A (2012). Modified acellular dermal matrix repair combined with opening suture and drainage in the treatment of Uygur high complex anal fistula in Xinjiang. Chi J Tissue Eng.

[54] Altomare DF, Greco VJ, Tricomi N, Arcanà F, Mancini S, Rinaldi M (2011). Seton or glue for trans-sphincteric anal fistulae: a prospective randomized crossover clinical trial. Colorectal Dis..

[55] Bondi J, Avdagic J, Karlbom U, Hallböök O, Kalman D, Šaltytė Benth J (2017). Randomized clinical trial comparing collagen plug and advancement flap for trans-sphincteric anal fistula. Br J Surg.

[56] Cwaliński J, Hermann J, Paszkowski J, Banasiewicz T (2021). Assessment of recurrent anal fistulas treatment with platelet-rich plasma. Arq Gastroenterol.

[57] de la Portilla F, Muñoz-Cruzado MVD, Maestre MV, García-Cabrera AM, Reyes ML, Vázquez-Monchul JM (2019). Plateletrich plasma (PRP) versus fibrin glue in cryptogenic fistula-in-ano: a phase III single-center, randomized, double-blind trial. Int J Colorectal Dis.

[58] Ellis CN, Clark S (2006). Fibrin glue as an adjunct to flap repair of anal fistulas: a randomized, controlled study. Dis Colon Rectum.

[59] Filingeri V, Gravante G, Baldessari E, Casciani CU (2004) Radiofrequency fistulectomy vs. diathermic fistulotomy for submucosal fistulas: a randomized trial. Eur Rev Med Pharmacol Sci 8(3):111–116. https://www.ncbi.nlm.nih.gov/pubmed/15368794 15368794

[60] Goudar BV, Dakhani NM (2020). A comparative study of Ligation of Intesphincteric Fistula Tract versus conventional fistulectomy in management of low fistula in ano: a randomized control trial. Int Surg J.

[61] Gupta PJ (2003). Radiosurgical fistulotomy; an alternative to conventional procedure in fistula in ano. Curr Surg.

[62] Han JG, Wang ZJ, Zheng Y, Chen CW, Wang XQ, Che XM (2016). Ligation of intersphincteric fistula tract vs ligation of the intersphincteric fistula tract plus a bioprosthetic anal fistula plug procedure in patients with transsphincteric anal fistula: early results of a multicenter prospective randomized trial. Ann Surg.

[63] Hermann J, Cwaliński J, Banasiewicz T, Kołodziejczak B (2022). Comparison between application of platelet rich plasma and mucosal advancement flap in patients with high transsphincteric anal fistulas: a randomized control trial. ANZ J Surg.

[64] Herreros MD, Garcia-Arranz M, Guadalajara H, De-La-Quintana P, Garcia-Olmo D, FATT Collaborative Group (2012). Autologous expanded adipose-derived stem cells for the treatment of complex cryptoglandular perianal fistulas: a phase III randomized clinical trial (FATT 1: fistula Advanced Therapy Trial 1) and long-term evaluation. Dis Colon Rectum.

[65] Ho KS, Tsang C, Seow-Choen F, Ho YH, Tang CL, Heah SM (2001). Prospective randomised trial comparing ayurvedic cutting seton and fistulotomy for low fistula-in-ano. Tech Coloproctol.

[66] Kalim M, Umerzai FK (2017). Comparison of mean healing time and mean scores between fistulectomy and fistulotomy for the treatment of low fistula in ano. J Postgrad Med Inst.

[67] Madbouly KM, Emile SH, Issa YA, Omar W (2021). Ligation of intersphincteric fistula tract (LIFT) with or without injection of platelet-rich plasma (PRP) in management of high trans-sphincteric fistula-in-ano: short-term outcomes of a prospective, randomized trial. Surgery.

[68] Mascagni D, Pironi D, Grimaldi G, Romani AM, La Torre G, Eberspacher C (2019). OTSC® Proctology vs. fistulectomy and primary sphincter reconstruction as a treatment for low trans-sphincteric anal fistula in a randomized controlled pilot trial. Minerva Chir.

[69] Perez F, Arroyo A, Serrano P, Sánchez A, Candela F, Perez MT (2006). Randomized clinical and manometric study of advancement flap versus fistulotomy with sphincter reconstruction in the management of complex fistula-in-ano. Am J Surg.

[70] Pescatori M, Ayabaca SM, Cafaro D, Iannello A, Magrini S (2006). Marsupialization of fistulotomy and fistulectomy wounds improves healing and decreases bleeding: a randomized controlled trial. Colorectal Dis.

[71] Rezk M, Emile SH, Fouda EY, Khaled N, Hamed M, Omar W (2022). Ligation of intersphincteric fistula tract (LIFT) with or without injection of bone marrow mononuclear cells in the treatment of trans-sphincteric anal fistula: a randomized controlled trial. J Gastrointest Surg.

[72] Sahakitrungruang C, Pattana-Arun J, Khomvilai S, Tantiphlachiva K, Atittharnsakul P, Rojanasakul A (2011) Marsupialization for simple fistula in ano: a randomized controlled trial. J Med Assoc Thai 94(6):699–703. https://www.ncbi.nlm.nih.gov/pubmed/21696078 21696078

[73] Singer M, Cintron J, Nelson R, Orsay C, Bastawrous A, Pearl R (2005). Treatment of fistulas-in-ano with fibrin sealant in combination with intra-adhesive antibiotics and/or surgical closure of the internal fistula opening. Dis Colon Rectum.

[74] Sørensen KM, Möller S, Qvist N (2021) Video-assisted anal fistula treatment versus fistulectomy and sphincter repair in the treatment of high cryptoglandular anal fistula: a randomized clinical study. BJS Open 5(5). 10.1093/bjsopen/zrab097 10.1093/bjsopen/zrab097 PMC8493008 34611700

[75] van Koperen PJ, Bemelman WA, Gerhards MF, Janssen LWM, van Tets WF, van Dalsen AD (2011). The anal fistula plug treatment compared with the mucosal advancement flap for cryptoglandular high transsphincteric perianal fistula: a double-blinded multicenter randomized trial. Dis Colon Rectum.

[76] Wang X, Wang C, Qi R (2021). Effectiveness and prognosis: drainage skin-bridge sparing surgery combined with fistulotomy versus fistulotomy only in the treatment of anal fistula. J Healthc Eng.

[77] Yan J, Ma L (2020). Clinical effect of tunnel-like fistulectomy plus draining Seton combined with incision of internal opening of anal fistula (TFSIA) in the treatment of high trans-sphincteric anal fistula. Med Sci Monit.

[78] Zhang Y, Li F, Zhao T, Cao F, Zheng Y, Li A (2020). Efficacy of video-assisted anal fistula treatment combined with closure of the internal opening using a stapler for Parks II anal fistula. Ann Transl Med.

[79] Khoshnevis J, Cuomo R, Karami F (2022). Jump technique versus seton method for anal fistula repair: a randomized controlled trial. J Invest Surg.

[80] Ortiz H, Marzo J, Ciga MA, Oteiza F, Armendáriz P, de Miguel M (2009). Randomized clinical trial of anal fistula plug versus endorectal advancement flap for the treatment of high cryptoglandular fistula in ano. Br J Surg.

[81] Dong X, Jia Z, Yu B, Zhang X, Xu F, Tan L (2020). Effect of intersphincteric fistula tract ligation versus anal fistulectomy on pain scores and serum levels of vascular endothelial growth factor and interleukin-2 in patients with simple anal fistulas. J Int Med Res.

[82] Goudar BV, Dakhani NM (2021). A comparative study of ligation of intesphincteric fistula tract versus conventional fistulectomy in management of low fistula in ano: a randomized control trial. Int Surg J.

[83] A ba-bai-ke-re MMTJ, Wen H, Huang HG (2010). Randomized controlled trial of minimally invasive surgery using acellular dermal matrix for complex anorectal fistula. World J Gastroenterol.

[84] Garcia-Arranz M, Garcia-Olmo D, Herreros MD (2020). Autologous adipose-derived stem cells for the treatment of complex cryptoglandular perianal fistula: a randomized clinical trial with long-term follow-up. Stem Cells Transl Med.

[85] Garcia-Olmo D, Herreros D, Pascual I (2009). Expanded adipose-derived stem cells for the treatment of complex perianal fistula: a phase II clinical trial. Dis Colon Rectum.

[86] Hammond TM, Porrett TR, Scott SM, Williams NS, Lunniss PJ (2011). Management of idiopathic anal fistula using cross-linked collagen: a prospective phase 1 study. Colorectal Dis.

[87] Kumar P, Sarthak S, Kumar Singh P, Mishra TS, Kumar SP (2022). Ligation of intersphincteric fistula tract vs endorectal advancement flap for high type fistula in ano: a randomized controlled trial (Frail Trial). J Am Coll Surg.

[88] Mushaya C, Bartlett L, Schulze B, Ho YH (2012). Ligation of intersphincteric fistula tract compared with advancement flap for complex anorectal fistulas requiring initial seton drainage. Am J Surg.

[89] Schwandner T, Thieme A, Scherer R (2018). Randomized clinical trial comparing a small intestinal submucosa anal fistula plug to advancement flap for the repair of complex anal fistulas. Int J Surg Open.

[90] van der Hagen SJ, Baeten CG, Soeters PB, van Gemert WG (2011). Staged mucosal advancement flap versus staged fibrin sealant in the treatment of complex perianal fistulas. Gastroenterol Res Pract.

[91] Madbouly KM, El Shazly W, Abbas KS, Hussein AM (2014). Ligation of intersphincteric fistula tract versus mucosal advancement flap in patients with high transsphincteric fistula-in-ano: a prospective randomized trial. Dis Colon Rectum.

[92] Al Sebai OI, Ammar MS, Mohamed SH, El Balshy MA (2021). Comparative study between intersphinecteric ligation of perianal fistula versus conventional fistulotomy with or without seton in the treatment of perianal fistula: a prospective randomized controlled trial. Ann Med Surg (Lond).

[93] Anan M, Emile SH, Elgendy H (2019). Fistulotomy with or without marsupialisation of wound edges in treatment of simple anal fistula: a randomised controlled trial. Ann R Coll Surg Engl.

[94] Chalya PL, Mabula JB (2013). Fistulectomy versus fistulotomy with marsupialisation in the treatment of low fistula-in- ano: a prospective randomized controlled trial. Tanzan J Health Res.

[95] Ho YH, Tan M, Leong AF, Seow-Choen F (1998). Marsupialization of fistulotomy wounds improves healing: a randomized controlled trial. Br J Surg.

[96] Jain BK, Vaibhaw K, Garg PK, Gupta S, Mohanty D (2012). Comparison of a fistulectomy and a fistulotomy with marsupialization in the management of a simple anal fistula: a randomized, controlled pilot trial. J Korean Soc Coloproctol.

[97] Kronborg O (1985). To lay open or excise a fistula-in-ano: a randomized trial. Br J Surg.

[98] Nazeer MA, Saleem R, Ali M, Ahmed ZN (2012) Better option for the patients of low fistula in ano: fistulectomy or fistulotomy. https://pjmhsonline.com/2012/oct_dec/pdf/888%20%20%20Better%20Option%20for%20the%20Patients%20of%20Low%20Fistula%20in%20Ano%20%20Fistulectomy%20or%20Fistulotomy.pdf. Accessed 22 Feb 2023

[99] Vinay G, Balasubrahmanya KS (2017). Comparative study on efficacy of fistulotomy and ligation of intersphincteric fistula tract (LIFT) procedure in management of fistula-in-ano. Int Surg J.

[100] Nour H, Abdelhamid MI, Abdel BA (2020). Fistulotomy wound edges; to marsupialize or not? In simple perianal fistula, a comparative clinical trial. Surg Chron.

[101] Elshamy MT, Emile SH, Abdelnaby M, Khafagy W, Elbaz SA (2022). A pilot randomized controlled trial on ligation of intersphincteric fistula tract (LIFT) versus modified parks technique and two-stage seton in treatment of complex anal fistula. Updates Surg.

[102] Huang H, Ji L, Gu Y, Li Y, Xu S (2022). Efficacy and safety of sphincter-preserving surgery in the treatment of complex anal fistula: a network meta-analysis. Front Surg.

[103] Wang Q, He Y, Shen J (2017). The best surgical strategy for anal fistula based on a network meta-analysis. Oncotarget.

[104] Rojanasakul A, Pattanaarun J, Sahakitrungruang C, Tantiphlachiva K (2007). Total anal sphincter saving technique for fistula-in-ano; the ligation of intersphincteric fistula tract. J Med Assoc Thai.

[105] Ji L, Zhang Y, Xu L, Wei J, Weng L, Jiang J (2020). Advances in the treatment of anal fistula: a mini-review of recent five-year clinical studies. Front Surg.

[106] Alasari S, Kim NK (2014). Overview of anal fistula and systematic review of ligation of the intersphincteric fistula tract (LIFT). Tech Coloproctol.

[107] Coremans G, Dockx S, Wyndaele J, Hendrickx A (2003). Do anal fistulas in Crohn’s disease behave differently and defy Goodsall's rule more frequently than fistulas that are cryptoglandular in origin?. Am J Gastroenterol.

[108] Halme L, Sainio AP (1995). Factors related to frequency, type, and outcome of anal fistulas in Crohn’s disease. Dis Colon Rectum.

[109] de Groof EJ, Cabral VN, Buskens CJ (2016). Systematic review of evidence and consensus on perianal fistula: an analysis of national and international guidelines. Colorectal Dis.

[110] Iqbal N, Machielsen AJHM, Kimman ML (2022). AFCOS: the development of a cryptoglandular anal fistula core outcome set. Ann Surg.

[111] Machielsen AJHM, Iqbal N, Kimman ML (2020). The development of a cryptoglandular anal fistula core outcome set (AFCOS): an international Delphi study protocol. United Eur Gastroenterol J.

