# Supplementary material for: Efficacy of different surgical treatments for management of anal fistula: a network meta-analysis
Source: Tech Coloproctol. 2023 Jul 17;27(10):827–45. doi: 10.1007/s10151-023-02845-8 (PMC10485107; doi:10.1007/s10151-023-02845-8)
Supplement: Supplementary file 1 — Supplementary file1 (DOCX 6035 KB) [file 10151_2023_2845_MOESM1_ESM.docx]

**Efficacy of different surgical treatments for management of anal fistula: a network meta-analysis**

Sameer Bhat, William Xu, Chris Varghese, Nandini Dubey, Cameron I. Wells, Chris Harmston, Greg O’Grady, Ian P. Bissett, Anthony Y. Lin

**TABLE OF CONTENTS**

[Supplementary Appendix S2: Search string for each database 2](#_Toc65438256)

[Supplementary Appendix S3: References of trials included in the network meta-analysis.](#_Toc65438257) 4

[Supplementary Appendix S4: Anal fistula definitions across the included trials](#_Toc65438257) 10

[Supplementary Figure S1: Summary](#_Toc65438257) quality assessment results derived using the Cochrane Collaboraiton's Risk of Bias 2.0 (ROB2) tool 13

[Supplementary Figure S2: Quality assessment of each included randomised controlled trial based on the five risk of bias domains using the Cochrane Collaboration’s Risk of Bias (ROB2) tool](#_Toc65438257) 14

[Supplementary Appendix S5: Analysis of transitivity for different surgical treatments in patients with simple and complex anal fistula](#_Toc65438257) 16

[Supplementary Appendix S6: Bayesian network meta-analysis results](#_Toc65438257) 20

[Supplementary Appendix S7: Narrative summary of sensitivity analyses results for each outcome when all treatments were included](#_Toc65438257) 37

[Supplementary Appendix S8: Inconsistency analysis across treatment comparisons for each outcome](#_Toc65438258) 39

[Supplementary Appendix S9: Heterogeneity analysis among each direct comparison of treatments for each outcome](#_Toc65438256) 42

[Supplementary Appendix S10: Individual author definitions for anal fistula healing among included trials](#_Toc65438256) 44

[Supplementary Appendix S11: Duration of follow-up after anal fistula surgery in each study](#_Toc65438256) 46

# **Supplementary Appendix S2.** Search string for each database

The search was conducted in October 2022.

**Ovid MEDLINE(R) Epub Ahead of Print, In Process & Other Non-Indexed Citations, Ovid MEDLINE (R) Daily, and Ovid MEDLINE (R) 1946-Present:**

1 Anal fistul*.mp

2 Anus fistul*.mp

3 Perianal fistul*.mp

4 Fistula-in-anal.mp

5 Fistula-in-ano.mp

6 FIA.mp

7 Anorectal fistul*.mp

8 1 OR 2 OR 3 OR 4 OR 5 OR 6 OR 7

9 Ligation.mp

10 Intersphincteric.mp

11 Fistula tract.mp

12 LIFT.mp

13 seton.mp

14 glue.mp

15 permacol paste.mp

16 advancement flap.mp

17 AF.mp

18 plug.mp

19 AFP.mp

20 fistula tract laser closure.mp

21 video assisted anal fistula treatment.mp

22 video endoscopic anal fistula treatment.mp

23 VAAFT.mp

24 laser.mp

25 FiLAC.mp

26 stem cell*.mp

27 mesenchymal.mp

28 adipose.mp

29 operati*.mp

30 closure.mp

31 fistulotomy.mp

32 laying open.mp

33 marsupialisation.mp

34 reconstruct*.mp

35 9 OR 10 OR 11 OR 12 OR 13 OR 14 OR 15 OR 16 OR 17 OR 18 OR 19 OR 20 OR 21 OR 22 OR 23 OR 24 OR 25 OR 26 OR 27 OR 28 OR 29 OR 30 OR 31 OR 32 OR 33 OR 34

36 8 AND 35

37 limit 36 to (humans AND all adult (19 plus years) AND randomized controlled trial)

#mp=title, abstract, original title, name of substance word, subject heading word, floating sub-heading word, keyword heading word, organism supplementary concept word, protocol supplementary concept word, rare disease supplementary concept word, unique identifier, synonyms.

**An identical search string was adapted for EMBASE (1980-October 2022) via the OVID platform, using the ‘explode’ function and Medical Subject Heading (MeSH) terms where available.**

**Cochrane Controlled Register of Trials (CENTRAL):**

#1 (Anal fistul*):ti,ab,kw OR (Anus fistul*):ti,ab,kw OR (Perianal fistul*):ti,ab,kw OR (Fistula-in-anal):ti,ab,kw OR (Fistula-in-ano):ti,ab,kw OR (FIA):ti,ab,kw OR (Anorectal fistul*):ti,ab,kw

#2 (Ligation):ti,ab,kw OR (intersphincteric):ti,ab,kw OR (fistula tract):ti,ab,kw OR (LIFT):ti,ab,kw OR (seton):ti,ab,kw OR (plug):ti,ab,kw OR (AFP):ti,ab,kw OR (fistula tract laser closure):ti,ab,kw OR (video assisted anal fistula treatment):ti,ab,kw OR (video endoscopic anal fistula treatment):ti,ab,kw OR (VAAFT):ti,ab,kw OR (laser):ti,ab,kw OR (FiLAC):ti,ab,kw OR (stem cell*):ti,ab,kw OR (mesenchymal):ti,ab,kw OR (adipose):ti,ab,kw OR (operati*):ti,ab,kw OR (closure):ti,ab,kw OR (fistulotomy):ti,ab,kw OR (laying open):ti,ab,kw OR (marsupialisation):ti,ab,kw OR (reconstruct*):ti,ab,kw

#3 #1 AND #2

ti=title; ab=abstract; kw=keywords.

# **Supplementary Appendix S3.** References of trials included in the network meta-analysis

1. A ba-bai-ke-re MMTJ, Wen H, Huang HG, Chu H, Lu M, Chang ZS, et al. Randomized controlled trial of minimally invasive surgery using acellular dermal matrix for complex anorectal fistula. World J Gastroenterol [Internet]. 2010 Jul 14;16(26):3279–86. Available from: http://dx.doi.org/10.3748/wjg.v16.i26.3279

2. A ba-bai-ke-re MMTJ, Wen H, Huang HG, Liang Z, Chu H, Er-ha-ti-hu-sai-yin A, et al. Modified acellular dermal matrix repair combined with opening suture and drainage in the treatment of Uygur high complex anal fistula in Xinjiang. Chi J Tissue Eng. 2012 August 19;16(34):6439-6444. Available from: https://doi.org/10.3969/j.issn.2095-4344.2012.34.033

3. Al Sebai OI, Ammar MS, Mohamed SH, El Balshy MA. Comparative study between intersphinecteric ligation of perianal fistula versus conventional fistulotomy with or without seton in the treatment of perianal fistula: A prospective randomized controlled trial. Ann Med Surg (Lond) [Internet]. 2021 Jan;61:180–4. Available from: http://dx.doi.org/10.1016/j.amsu.2020.12.014

4. Altomare DF, Greco VJ, Tricomi N, Arcanà F, Mancini S, Rinaldi M, et al. Seton or glue for trans-sphincteric anal fistulae: a prospective randomized crossover clinical trial. Colorectal Dis [Internet]. 2011 Jan;13(1):82–6. Available from: http://dx.doi.org/10.1111/j.1463-1318.2009.02056.x

5. Anan M, Emile SH, Elgendy H, Shalaby M, Elshobaky A, Abdel-Razik MA, et al. Fistulotomy with or without marsupialisation of wound edges in treatment of simple anal fistula: a randomised controlled trial. Ann R Coll Surg Engl [Internet]. 2019 Sep;101(7):472–8. Available from: http://dx.doi.org/10.1308/rcsann.2019.0057

6. Bondi J, Avdagic J, Karlbom U, Hallböök O, Kalman D, Šaltytė Benth J, et al. Randomized clinical trial comparing collagen plug and advancement flap for trans-sphincteric anal fistula. Br J Surg [Internet]. 2017 Jul 13;104(9):1160–6. Available from: http://dx.doi.org/10.1002/bjs.10549

7. Chalya PL, Mabula JB. Fistulectomy versus fistulotomy with marsupialisation in the treatment of low fistula-in- ano: a prospective randomized controlled trial. Tanzan J Health Res [Internet]. 2013 Jul;15(3):193–8. Available from: http://dx.doi.org/10.4314/thrb.v15i3.7

8. Chen XL, Huang ZH, Zhan YQ, Liu MS, Sheng G, Xu M, et al. A minimally invasive approach in the treatment of complicated anal fistula through spatium intermuscular of anal sphincter. Ch J Gastrointest Surg. 2005 Jul;8(4):322-324.

9. Cwaliński J, Hermann J, Paszkowski J, Banasiewicz T. Assessment of recurrent anal fistulas treatment with platelet-rich plasma. Arq Gastroenterol [Internet]. 2021 Apr;58(2):185–9. Available from: http://dx.doi.org/10.1590/S0004-2803.202100000-32

10. de la Portilla F, Muñoz-Cruzado MVD, Maestre MV, García-Cabrera AM, Reyes ML, Vázquez-Monchul JM, et al. Platelet-rich plasma (PRP) versus fibrin glue in cryptogenic fistula-in-ano: a phase III single-center, randomized, double-blind trial. Int J Colorectal Dis [Internet]. 2019 Jun;34(6):1113–9. Available from: http://dx.doi.org/10.1007/s00384-019-03290-6

11. Dong X, Jia Z, Yu B, Zhang X, Xu F, Tan L. Effect of intersphincteric fistula tract ligation versus anal fistulectomy on pain scores and serum levels of vascular endothelial growth factor and interleukin-2 in patients with simple anal fistulas. J Int Med Res [Internet]. 2020 Sep;48(9):300060520949072. Available from: http://dx.doi.org/10.1177/0300060520949072

12. Ellis CN, Clark S. Fibrin glue as an adjunct to flap repair of anal fistulas: a randomized, controlled study. Dis Colon Rectum [Internet]. 2006 Nov;49(11):1736–40. Available from: http://dx.doi.org/10.1007/s10350-006-0718-8

13. Elshamy MT, Emile SH, Abdelnaby M, Khafagy W, Elbaz SA. A pilot randomized controlled trial on ligation of intersphincteric fistula tract (LIFT) versus modified parks technique and two-stage seton in treatment of complex anal fistula. Updates Surg [Internet]. 2022 Apr;74(2):657–66. Available from: http://dx.doi.org/10.1007/s13304-022-01240-6

14. Filingeri V, Gravante G, Baldessari E, Casciani CU. Radiofrequency fistulectomy vs. diathermic fistulotomy for submucosal fistulas: a randomized trial. Eur Rev Med Pharmacol Sci [Internet]. 2004 May;8(3):111–6. Available from: https://www.ncbi.nlm.nih.gov/pubmed/15368794

15. Garcia-Arranz M, Garcia-Olmo D, Herreros MD, Gracia-Solana J, Guadalajara H, de la Portilla F, et al. Autologous adipose-derived stem cells for the treatment of complex cryptoglandular perianal fistula: A randomized clinical trial with long-term follow-up. Stem Cells Transl Med [Internet]. 2020 Mar;9(3):295–301. Available from: http://dx.doi.org/10.1002/sctm.19-0271

16. Garcia-Olmo D, Herreros D, Pascual I, Pascual JA, Del-Valle E, Zorrilla J, et al. Expanded adipose-derived stem cells for the treatment of complex perianal fistula: a phase II clinical trial. Dis Colon Rectum [Internet]. 2009 Jan;52(1):79–86. Available from: http://dx.doi.org/10.1007/DCR.0b013e3181973487

17. Goudar BV, Dakhani NM. A comparative study of Ligation of Intesphincteric Fistula Tract versus conventional fistulectomy in management of low fistula in ano: a randomized control trial. Int Surg J [Internet]. 2020 Dec 28;8(1):261. Available from: http://dx.doi.org/10.18203/2349-2902.isj20205890

18. Gupta PJ. Radiosurgical fistulotomy; an alternative to conventional procedure in fistula in ano. Curr Surg [Internet]. 2003 Sep;60(5):524–8. Available from: http://dx.doi.org/10.1016/S0149-7944(03)00082-5

19. Hammond TM, Porrett TR, Scott SM, Williams NS, Lunniss PJ. Management of idiopathic anal fistula using cross-linked collagen: a prospective phase 1 study. Colorectal Dis [Internet]. 2011 Jan;13(1):94–104. Available from: http://dx.doi.org/10.1111/j.1463-1318.2009.02087.x

20. Han JG, Wang ZJ, Zheng Y, Chen CW, Wang XQ, Che XM, et al. Ligation of intersphincteric fistula tract vs ligation of the intersphincteric fistula tract plus a bioprosthetic anal fistula plug procedure in patients with transsphincteric anal fistula: Early results of a multicenter prospective randomized trial. Ann Surg [Internet]. 2016 Dec;264(6):917–22. Available from: http://dx.doi.org/10.1097/SLA.0000000000001562

21. Hermann J, Cwaliński J, Banasiewicz T, Kołodziejczak B. Comparison between application of platelet rich plasma and mucosal advancement flap in patients with high transsphincteric anal fistulas: a randomized control trial. ANZ J Surg [Internet]. 2022 May;92(5):1137–41. Available from: http://dx.doi.org/10.1111/ans.17656

22. Herreros MD, Garcia-Arranz M, Guadalajara H, De-La-Quintana P, Garcia-Olmo D, FATT Collaborative Group. Autologous expanded adipose-derived stem cells for the treatment of complex cryptoglandular perianal fistulas: a phase III randomized clinical trial (FATT 1: fistula Advanced Therapy Trial 1) and long-term evaluation. Dis Colon Rectum [Internet]. 2012 Jul;55(7):762–72. Available from: http://dx.doi.org/10.1097/DCR.0b013e318255364a

23. Ho YH, Tan M, Leong AF, Seow-Choen F. Marsupialization of fistulotomy wounds improves healing: a randomized controlled trial. Br J Surg [Internet]. 1998 Jan;85(1):105–7. Available from: http://dx.doi.org/10.1046/j.1365-2168.1998.00529.x

24. Ho KS, Tsang C, Seow-Choen F, Ho YH, Tang CL, Heah SM, et al. Prospective randomised trial comparing ayurvedic cutting seton and fistulotomy for low fistula-in-ano. Tech Coloproctol [Internet]. 2001 Dec;5(3):137–41. Available from: http://dx.doi.org/10.1007/s101510100015

25. Ho KS, Ho YH. Controlled, randomized trial of island flap anoplasty for treatment of trans-sphincteric fistula-in-ano: early results. Tech Coloproctol [Internet]. 2005 Jul;9(2):166–8. Available from: http://dx.doi.org/10.1007/s10151-005-0220-7

26. Jain BK, Vaibhaw K, Garg PK, Gupta S, Mohanty D. Comparison of a fistulectomy and a fistulotomy with marsupialization in the management of a simple anal fistula: a randomized, controlled pilot trial. J Korean Soc Coloproctol [Internet]. 2012 Apr;28(2):78–82. Available from: http://dx.doi.org/10.3393/jksc.2012.28.2.78

27. Kalim M, Umerzai FK. Comparison of mean healing time and mean scores between fistulectomy and fistulotomy for the treatment of low fistula in ano. J Postgrad Med Inst 2017; 31(2):118-21.

28. Khoshnevis J, Cuomo R, Karami F, Dashti T, Kalantar Motamedi A, Kalantar Motamedi M, et al. Jump technique versus Seton Method for anal fistula repair: A randomized controlled trial. J Invest Surg [Internet]. 2022 Jun;35(6):1217–23. Available from: http://dx.doi.org/10.1080/08941939.2021.2022252

29. Kronborg O. To lay open or excise a fistula-in-ano: a randomized trial. Br J Surg [Internet]. 1985 Dec;72(12):970. Available from: http://dx.doi.org/10.1002/bjs.1800721211

30. Kumar P, Sarthak S, Kumar Singh P, Mishra TS, Kumar Sasmal P. Ligation of intersphincteric fistula tract vs endorectal advancement flap for high type fistula in Ano: A randomized controlled trial (frail trial). J Am Coll Surg [Internet]. 2022 Nov;235(5):S15–S15. Available from: http://dx.doi.org/10.1097/01.xcs.0000895736.81221.f2

31. Madbouly KM, El Shazly W, Abbas KS, Hussein AM. Ligation of intersphincteric fistula tract versus mucosal advancement flap in patients with high transsphincteric fistula-in-ano: a prospective randomized trial. Dis Colon Rectum [Internet]. 2014 Oct;57(10):1202–8. Available from: http://dx.doi.org/10.1097/DCR.0000000000000194

32. Madbouly KM, Emile SH, Issa YA, Omar W. Ligation of intersphincteric fistula tract (LIFT) with or without injection of platelet-rich plasma (PRP) in management of high trans-sphincteric fistula-in-ano: Short-term outcomes of a prospective, randomized trial. Surgery [Internet]. 2021 Jul;170(1):61–6. Available from: http://dx.doi.org/10.1016/j.surg.2020.12.025

33. Mascagni D, Pironi D, Grimaldi G, Romani AM, La Torre G, Eberspacher C, et al. OTSC® Proctology vs. fistulectomy and primary sphincter reconstruction as a treatment for low trans-sphincteric anal fistula in a randomized controlled pilot trial. Minerva Chir [Internet]. 2019 Feb;74(1):1–6. Available from: http://dx.doi.org/10.23736/S0026-4733.18.07617-4

34. Mushaya C, Bartlett L, Schulze B, Ho YH. Ligation of intersphincteric fistula tract compared with advancement flap for complex anorectal fistulas requiring initial seton drainage. Am J Surg [Internet]. 2012 Sep;204(3):283–9. Available from: http://dx.doi.org/10.1016/j.amjsurg.2011.10.025

35. Nazeer MA, Saleem R, Ali M, Ahmed ZN. Better Option for the Patients of Low Fistula In Ano: Fistulectomy or Fistulotomy. Pak J Med Health Sci. 2012 Dec;6(4):888-90.

36. Nour H, Abdelhamid MI, Bari AA. Fistulotomy wound edges; to marsupialize or not? in simple perianal fistula, a comparative clinical trial. Surg Chron. 2020;25(2):126-29.

37. Ortiz H, Marzo J, Ciga MA, Oteiza F, Armendáriz P, de Miguel M. Randomized clinical trial of anal fistula plug versus endorectal advancement flap for the treatment of high cryptoglandular fistula in ano. Br J Surg [Internet]. 2009 Jun;96(6):608–12. Available from: http://dx.doi.org/10.1002/bjs.6613

38. Perez F, Arroyo A, Serrano P, Sánchez A, Candela F, Perez MT, et al. Randomized clinical and manometric study of advancement flap versus fistulotomy with sphincter reconstruction in the management of complex fistula-in-ano. Am J Surg [Internet]. 2006 Jul;192(1):34–40. Available from: http://dx.doi.org/10.1016/j.amjsurg.2006.01.028

39. Pescatori M, Ayabaca SM, Cafaro D, Iannello A, Magrini S. Marsupialization of fistulotomy and fistulectomy wounds improves healing and decreases bleeding: a randomized controlled trial. Colorectal Dis [Internet]. 2006 Jan;8(1):11–4. Available from: http://dx.doi.org/10.1111/j.1463-1318.2005.00835.x

40. Rezk M, Emile SH, Fouda EY, Khaled N, Hamed M, Omar W, et al. Ligation of intersphincteric fistula tract (LIFT) with or without injection of bone marrow mononuclear cells in the treatment of trans-sphincteric anal fistula: A randomized controlled trial. J Gastrointest Surg [Internet]. 2022 Jun;26(6):1298–306. Available from: http://dx.doi.org/10.1007/s11605-022-05316-x

41. Sahakitrungruang C, Pattana-Arun J, Khomvilai S, Tantiphlachiva K, Atittharnsakul P, Rojanasakul A. Marsupialization for simple fistula in ano: a randomized controlled trial. J Med Assoc Thai [Internet]. 2011 Jun;94(6):699–703. Available from: https://www.ncbi.nlm.nih.gov/pubmed/21696078

42. Schwandner T, Thieme A, Scherer R, Hodde JP, Sötje U, Roblick M, et al. Randomized clinical trial comparing a small intestinal submucosa anal fistula plug to advancement flap for the repair of complex anal fistulas. Int J Surg Open [Internet]. 2018;15:25–31. Available from: http://dx.doi.org/10.1016/j.ijso.2018.10.007

43. Singer M, Cintron J, Nelson R, Orsay C, Bastawrous A, Pearl R, et al. Treatment of fistulas-in-ano with fibrin sealant in combination with intra-adhesive antibiotics and/or surgical closure of the internal fistula opening. Dis Colon Rectum [Internet]. 2005 Apr;48(4):799–808. Available from: http://dx.doi.org/10.1007/s10350-004-0898-z

44. Sørensen KM, Möller S, Qvist N. Video-assisted anal fistula treatment versus fistulectomy and sphincter repair in the treatment of high cryptoglandular anal fistula: a randomized clinical study. BJS Open [Internet]. 2021 Sep 6;5(5). Available from: http://dx.doi.org/10.1093/bjsopen/zrab097

45. van der Hagen SJ, Baeten CG, Soeters PB, van Gemert WG. Staged mucosal advancement flap versus staged fibrin sealant in the treatment of complex perianal fistulas. Gastroenterol Res Pract [Internet]. 2011 Jul 26;2011:186350. Available from: http://dx.doi.org/10.1155/2011/186350

46. van Koperen PJ, Bemelman WA, Gerhards MF, Janssen LWM, van Tets WF, van Dalsen AD, et al. The anal fistula plug treatment compared with the mucosal advancement flap for cryptoglandular high transsphincteric perianal fistula: a double-blinded multicenter randomized trial. Dis Colon Rectum [Internet]. 2011 Apr;54(4):387–93. Available from: http://dx.doi.org/10.1007/DCR.0b013e318206043e

47. Vinay G, Balasubrahmanya KS. Comparative study on efficacy of fistulotomy and Ligation of intersphincteric fistula tract (LIFT) procedure in management of fistula-in-ano. Int Surg J [Internet]. 2017 Sep 27;4(10):3406. Available from: http://dx.doi.org/10.18203/2349-2902.isj20174505

48. Wang C, Lu JG, Cao YQ, Yao YB, Guo XT, Yin HQ. Traditional Chinese surgical treatment for anal fistulae with secondary tracks and abscess. World J Gastroenterol [Internet]. 2012 Oct 28;18(40):5702–8. Available from: http://dx.doi.org/10.3748/wjg.v18.i40.5702

49. Wang X, Wang C, Qi R. Effectiveness and prognosis: Drainage skin-bridge sparing surgery combined with fistulotomy versus fistulotomy only in the treatment of anal fistula. J Healthc Eng [Internet]. 2021 Nov 28;2021:6940072. Available from: http://dx.doi.org/10.1155/2021/6940072

50. Wu YF, Zheng BC, Chen Q, Chen XD, Ye SS, Lin QY, et al. Video-assisted modified ligation of the intersphincteric fistula tract, an integration of 2 minimally invasive techniques for the treatment of Parks type II anal fistulas. Surg Innov [Internet]. 2021 Aug;28(4):419–26. Available from: http://dx.doi.org/10.1177/1553350620978026

51. Yan J, Ma L. Clinical effect of tunnel-like fistulectomy plus draining Seton combined with incision of internal opening of anal fistula (TFSIA) in the treatment of high trans-sphincteric anal fistula. Med Sci Monit [Internet]. 2020 Jan 13;26:e918228. Available from: http://dx.doi.org/10.12659/MSM.918228

52. Zhang Y, Li F, Zhao T, Cao F, Zheng Y, Li A. Efficacy of video-assisted anal fistula treatment combined with closure of the internal opening using a stapler for Parks II anal fistula. Ann Transl Med [Internet]. 2020 Nov;8(22):1517. Available from: http://dx.doi.org/10.21037/atm-20-7154

# **Supplementary Appendix S4.** Anal fistula definitions across the included trials

| **First author (year)** | **Anal fistula classification** | **Definition** |
| --- | --- | --- |
| A ba-bai-ke-re (2010) | Complex | Intra-sphincteric and trans-sphincteric anorectal fistulae which are 2-6cm in length (identified with a fistula probe during surgery) |
| A ba-bai-ke-re (2012) | Complex | Fistulas with either: *i)* more than one tract, *ii)* the main tract passing through the deep layer of the external anal sphincter or rectal ring, *iii)* more than one inner opening, or *iv)* the inner mouth is above the rectal ring |
| Al Sebai (2021) | Simple | Low trans-sphincteric perianal fistulae diagnosed on MRI |
| Altomare (2011) | Complex | Medium or high trans-sphincteric anal fistulae evaluated using endoanal ultrasound |
| Anan (2019) | Simple | Primary inter-sphincteric and low trans-sphincteric fistula involving <1/3 of the external anal sphincter |
| Bondi (2017) | Complex | Single, continuous trans-sphincteric cryptoglandular fistula tract >2cm in length and involving >1/3 of the external anal sphincter |
| Chalya (2013) | Simple | Low fistula-in-ano with a single internal and single external opening, with the absence of a secondary tract |
| Chen (2005) | Complex | “Complicated anal fistula” (characteristics not further specified) |
| Cwalinski (2021) | Complex | Recurrent cryptoglandular anal fistula after at least one cutting procedure, with one or at most two active channels |
| de la Portilla (2019) | Complex | High cryptoglandular anal fistula with a single internal opening and traversing >1/3 of the coronal length of the anal sphincter (as measured endosonographically), or with trans- or supra-levator extension detected on imaging |
| Dong (2020) | Simple | Endoanal ultrasonographic diagnosis (anal fistula characteristics not further specified) |
| Ellis (2006) | Complex | Fistula tract involving >30 to 50% of the sphincter mechanism, or was located anteriorly (in females), and with pre-existing faecal incontinence |
| Elshamy (2022) | Complex | High trans-sphincteric (involving more than 30% of the external anal sphincter), extra-sphincteric, supra-sphincteric, horse-shoe, and anterior fistulas (in females) which were not secondary to: IBD, STD, TB, malignancy, or radiation |
| Filingeri (2004) | Simple | Submucosal fistula with posterior internal and external orifices |
| Garcia-Arranz (2020) | Complex | Perianal fistula of cryptoglandular origin with at least one of the following: *i)* some degree of faecal incontinence, *ii)* extra-sphincteric, *iii)* supra-sphincteric, and *iv)* high trans-sphincteric fistula (diagnosed by physical examination and MRI), without any indications of IBD |
| Garcia-Olmo (2009) | Complex | Perianal fistula with a visible external opening and at least one of the following: *i)* fistula tract under the perianal skin unidentifiable in the physical examination; *ii)* fistula tract parallel to the rectum when examined with a probe; *iii)* associated faecal incontinence; *iv)* at least one previous operation performed because of fistulous disease (e.g., fistulectomy or advancement flap); *v)* supra-sphincteric tracts and *vi)* rectovaginal fistula |
| Goudar (2020) | Simple | Low fistula-in-ano (characteristics not further specified) |
| Gupta (2003) | Simple | Low anal fistulas, with tracks that do not extend above the level of the anal crypts and usually open at this level in the anal canal |
| Hammond (2009) | Complex | Idiopathic fistulae in whom fistulotomy was deemed unsuitable based on fistula type and level and threat to continence status |
| Han (2016) | Complex | High trans-sphincteric fistula involving >30% of the external anal sphincter, with more than two fistula tracts and without active sepsis or abscess |
| Hermann (2022) | Complex | High trans-sphincteric anal fistulas (affecting the middle or upper third part of the anal sphincter) identified DRE, probing and/or dyeing of the fistulous tracts, or with trans-rectal ultrasound |
| Herreros (2012) | Complex | Single solitary draining cryptoglandular fistula-in-ano and with no: *i)* no identifiable fistula tract under the perianal skin and/or parallel to the rectum; *ii)* faecal incontinence in the trans-sphincteric fistula; *iii)* risk factors for anal incontinence; *iv)* previous operation due to fistulous disease (e.g., fistulectomy or advancement flap), and *v)* supra-sphincteric tracts shown by MRI |
| Ho (1998) | Simple | Uncomplicated inter-sphincteric or trans-sphincteric fistula-in-ano examined by palpation and proctoscopy, and without pre-existing incontinence to gas, liquid, and solid stools |
| Ho (2001) | Simple | Trans-sphincteric or inter-sphincteric fistula-in-ano diagnosed on transanal ultrasound scan |
| Ho (2005) | Simple | Trans-sphincteric fistula-in-ano |
| Jain (2012) | Simple | Clinical diagnosis of anal fistula with: *i)* one internal and one external opening, *ii)* a completely palpable tract, and no palpable abnormality in the upper anal canal or the lower rectum, *iii)* low trans-sphincteric fistula (involving less than the lower third of the anal sphincter), *iv)* inter-sphincteric fistula or subcutaneous fistula and *v)* absence of a secondary tract |
| Kalim (2017) | Simple | Low fistula-in-ano diagnosed by the presence of all of: *i)* a track connecting the anal canal to the skin around the anus; *ii)* the external opening and tract examined by DRE while the internal opening is identified by proctoscopy; *iii)* visualisation of the fistula tract by injecting contrast through anteroposterior, lateral, and oblique x-ray images |
| Khoshnevis (2022) | Complex | Multiple fistula tracts and openings, women with anterior fistula, and with tracts traversing approximately 30% of the external sphincter (during palpation and probing under anaesthesia) |
| Kronborg (1985) | Simple | Single track below the anorectal ring |
| Kumar (2022) | Complex | High fistula-in-ano showing >30% sphincter involvement, and with a single external opening (on MRI) |
| Madbouly (2014) | Complex | High trans-sphincteric fistulas involving the upper half of the sphincter complex |
| Madbouly (2021) | Complex | High trans-sphincteric fistulas involving >50% of the anal sphincter (on MRI) |
| Mascagni (2018) | Simple | Low trans-sphincteric fistula diagnosed on clinical and rectal examination |
| Mushaya (2012) | Complex | Trans-sphincteric fistulas requiring previous seton drainage, and with: *i)* tracts crossing >30% of the external anal sphincter; *ii)* anterior fistula (in women); *iii)* multiple tracts; *iv)* recurrent fistula; *v)* pre-existing incontinence |
| Nazeer (2012) | Simple | Low fistula-in-ano |
| Nour (2020) | Simple | Fistula characteristics not further specified |
| Ortiz (2009) | Complex | High trans-sphincteric fistula-in-ano with a single tract involving the upper 2/3 of the external sphincter complex (confined by the puborectalis sling and the end of the anal canal) [diagnosed based on DRE, proctosigmoidoscopy and hydrogen peroxide-enhanced ultrasonography] |
| Perez (2006) | Complex | Primary fistulas with high trans-sphincteric and supra-sphincteric tracks |
| Pescatori (2006) | Complex | High, recurrent and horseshoe fistula |
| Rezk (2022) | Simple | Trans-sphincteric anal fistula (characteristics not further specified) |
| Sahakitrungruang (2011) | Simple | Simple, uncomplicated fistula-in-ano, with a tract depth not beyond the subcutaneous external sphincter (involvement <5mm in thickness) |
| Schwandner (2018) | Complex | Primary, persistent anal fistulas (confirmed by physical examination and endoanal ultrasound) [characteristics not further specified) |
| Singer (2005) | Complex | Fistula-in-ano lasting at least 3 months (characteristics not further specified) |
| Sorenson (2021) | Complex | High, trans-sphincteric anal fistula involving >1/3 of the external anal sphincter |
| van der Hagen (2011) | Complex | High trans-sphincteric, supra-sphincteric, and extra-sphincteric fistula tracts originating from the middle third or upper part of the anal sphincter |
| van Koperen (2011) | Simple | Trans-sphincteric involving the upper two-thirds of the sphincter complex (and was confined by the puborectal sling at the end of the anal canal), recurrent, high, with multiple tracts, or anterior fistula (in females) |
| Vinay (2017) | Simple | Fistula characteristics not specified |
| Wang (2012) | Complex | Inter-sphincteric or trans-sphincteric fistulas with secondary tracks and abscess |
| Wang (2021) | Simple | Fistula length <2cm |
| Wu (2021) | Simple | Anal fistulas associated with glandular infection, and without evidence of: anal stenosis, infection, and/or deformity |
| Yan (2020) | Complex | High trans-sphincteric anal fistula with infection of cryptoglandular origin |
| Zhang (2020) | Complex | Anal fistula with normal anal function and without evidence of laxity, stenosis, infection, or malformations (diagnosed via ultrasound or MRI) |

DRE, digital rectal examination; IBD, inflammatory bowel disease; MRI, magnetic resonance imaging; STD, sexually transmitted disease; TB, tuberculosis.

# **Supplementary Figure S1.** Summary quality assessment results derived using the Cochrane Collaboration’s Risk of Bias 2.0 (ROB2) tool.

# **Supplementary Figure S2.** Quality assessment of each included randomised controlled trial based on the five risk of bias domains using the Cochrane Collaboration’s Risk of Bias (ROB2) tool


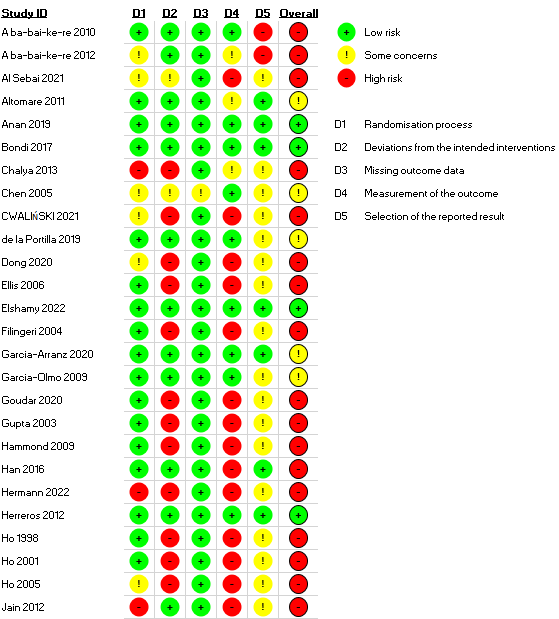


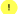

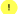

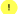

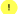

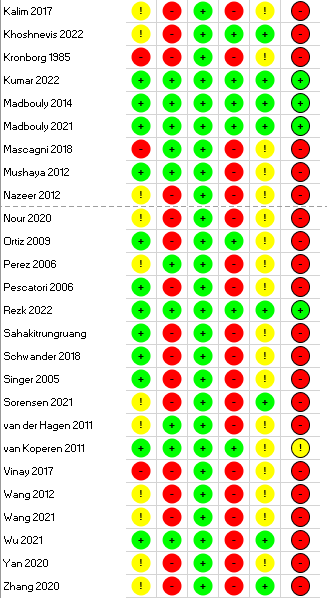


# **Supplementary Appendix S5.** Analysis of transitivity for different surgical treatments in patients with simple and complex anal fistula

**A) Mean age**

*Simple anal fistula*


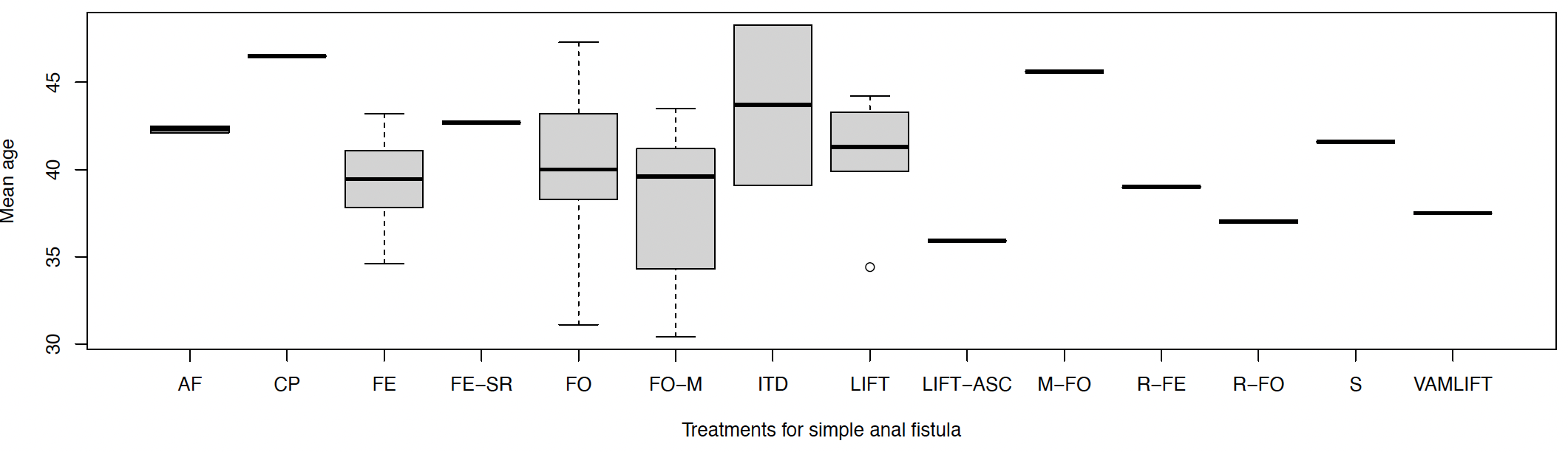


*Complex anal fistula*

**
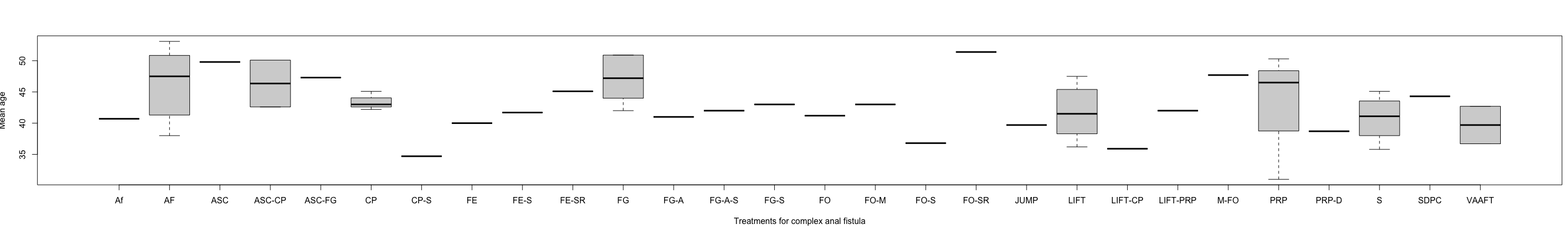
**

**B) Sex (% female)**

*
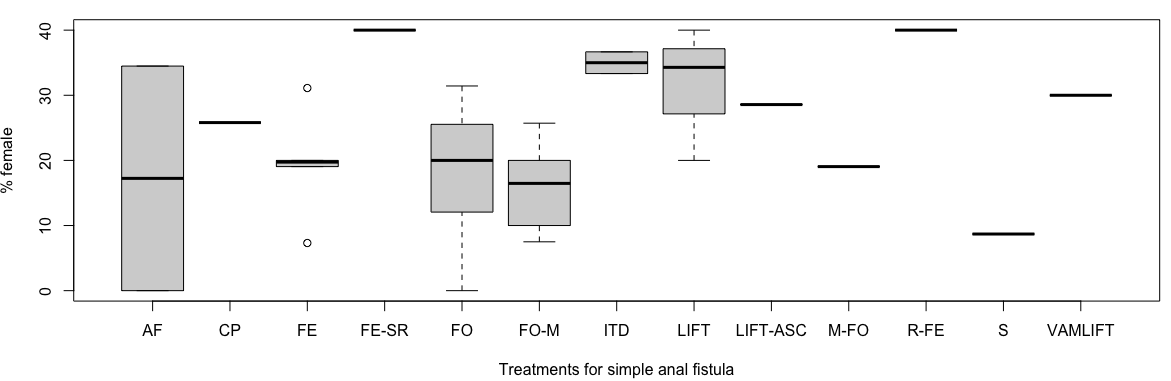
Simple anal fistula*

*Complex anal fistula*


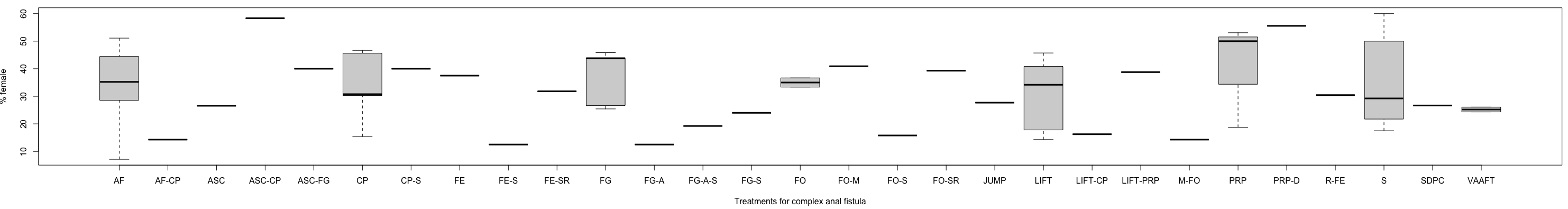


**C) Geographic trial locations**

*Simple anal fistula*


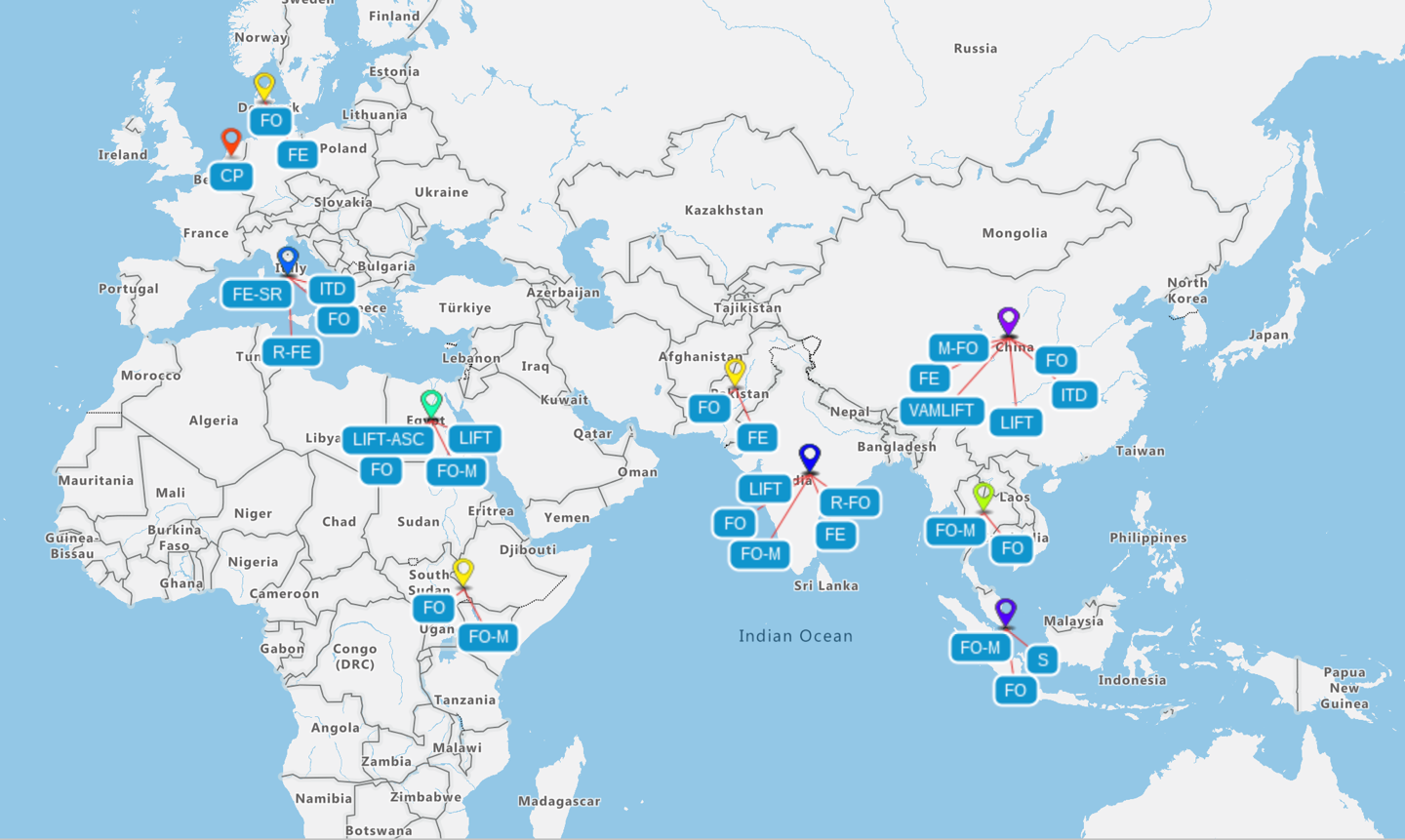


*Complex anal fistula*


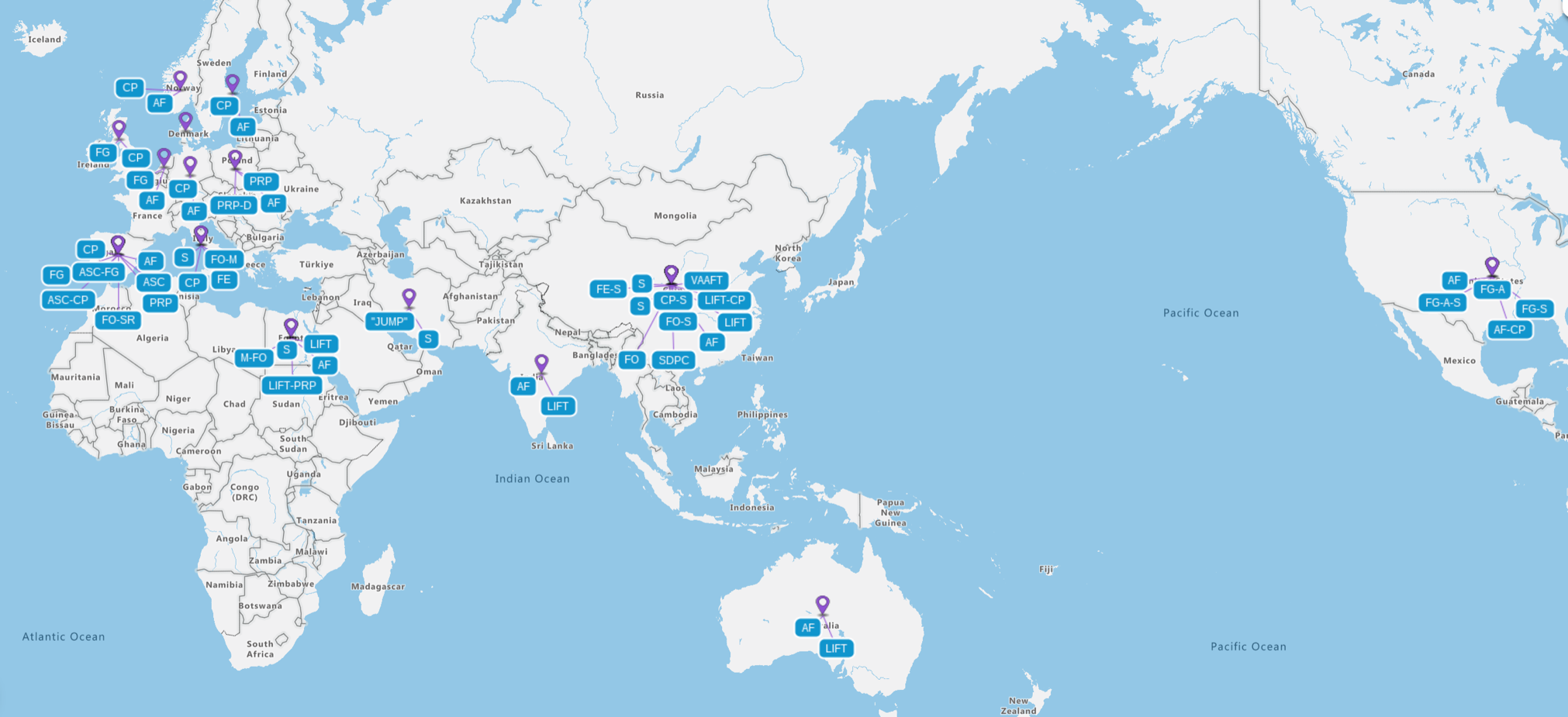


# **Supplementary Appendix S6.** Bayesian network meta-analysis results

**Primary outcomes**

Short-term success (≤6 months after surgery) for simple anal fistula

***Table S1.*** *SUCRA ranking probabilities among treatment comparisons for short-term success in patients with simple anal fistula*

| **Treatment** | **No. of trials** | **Total participants** | **SUCRA value (rank)** |
| --- | --- | --- | --- |
| **Sensitivity analysis*** | | | |
| Fistulectomy | 2 | 75 | 64.9 (1) |
| LIFT combined with adipose-derived stem cells | 1 | 35 | 51.3 (2) |
| Fistulotomy | 1 | 10 | 47.4 (3) |
| Advancement flap | 1 | 10 | 47.0 (4) |
| LIFT | 3 | 110 | 41.2 (5) |

LIFT, ligation of the inter-sphincteric fistula tract; SUCRA, surface under the cumulative ranking.

*Includes all treatments.

*
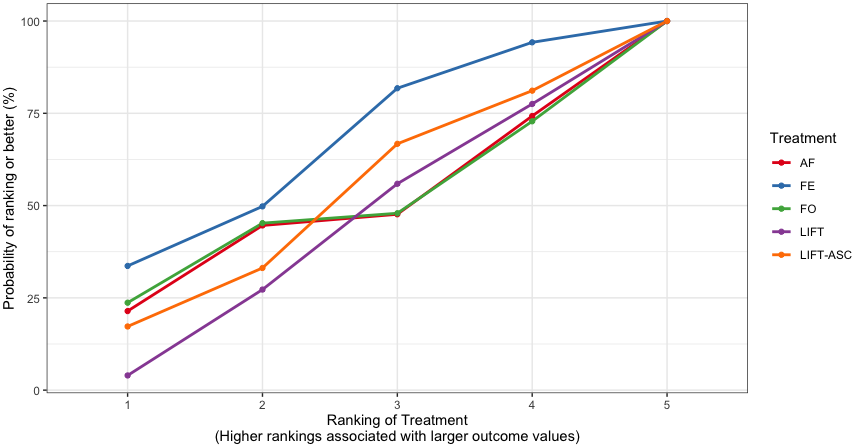
*
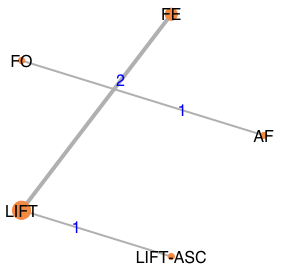
 *Network plot SUCRA curve*


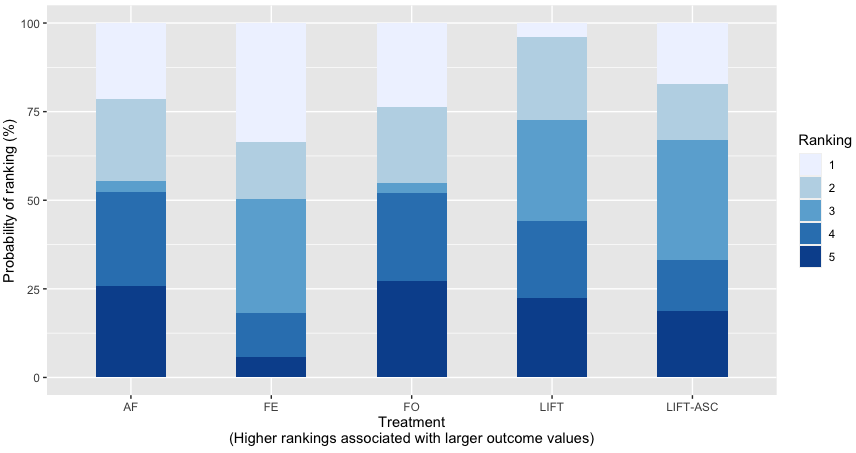
*Rankogram plot Forest plot*


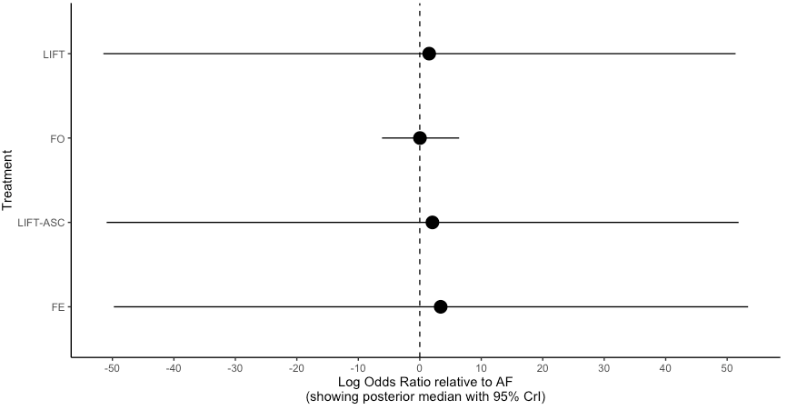


*Heat plot*


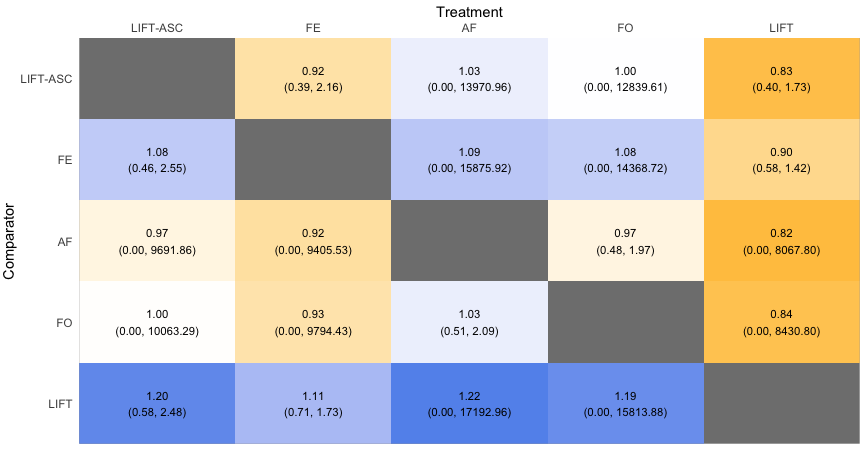


Long-term success (>6 months after surgery) for simple anal fistula

***Table S2.*** *SUCRA ranking probabilities among treatment comparisons for long-term success in patients with simple anal fistula*

| **Treatment** | **No. of trials** | **Total participants** | **SUCRA value (rank)** |
| --- | --- | --- | --- |
| **Sensitivity analysis*** | | | |
| Fistulectomy with primary sphincter reconstruction | 1 | 15 | 96.2 (1) |
| Incision-thread drawing | 2 | 52 | 38.5 (2) |
| Video-assisted modified LIFT | 1 | 37 | 15.3 (3) |

LIFT, ligation of the inter-sphincteric fistula tract; SUCRA, surface under the cumulative ranking.

*Includes all treatments.

*
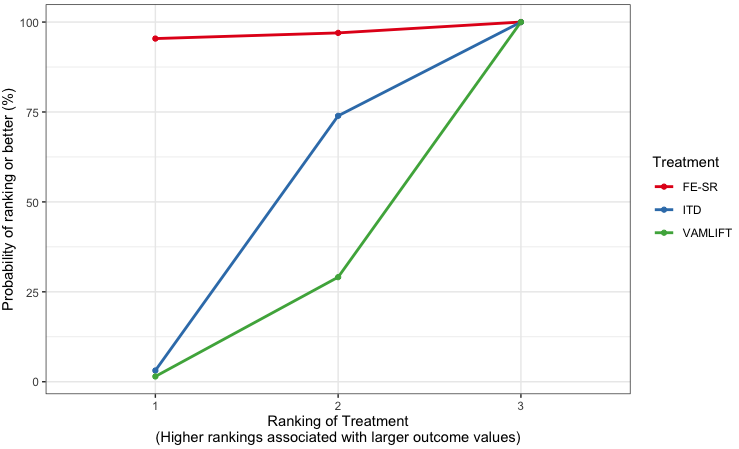
*
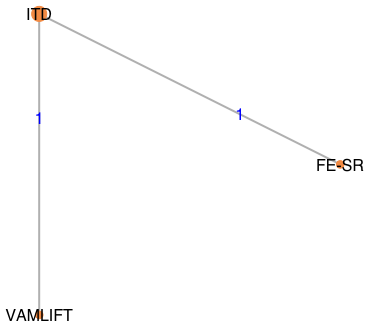
*Network plot* *SUCRA curve*


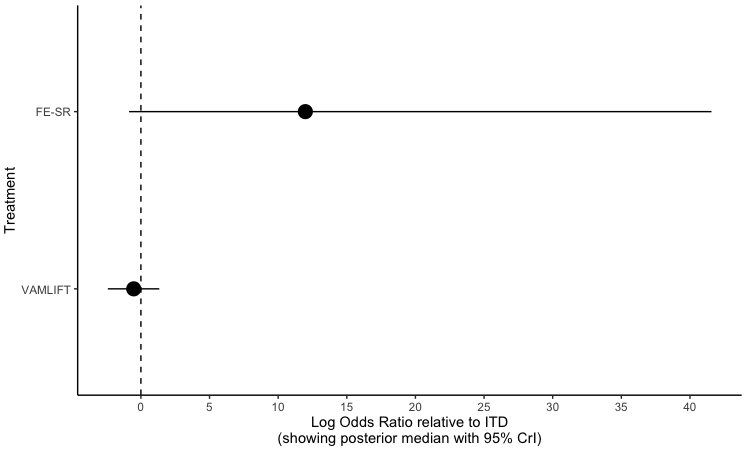

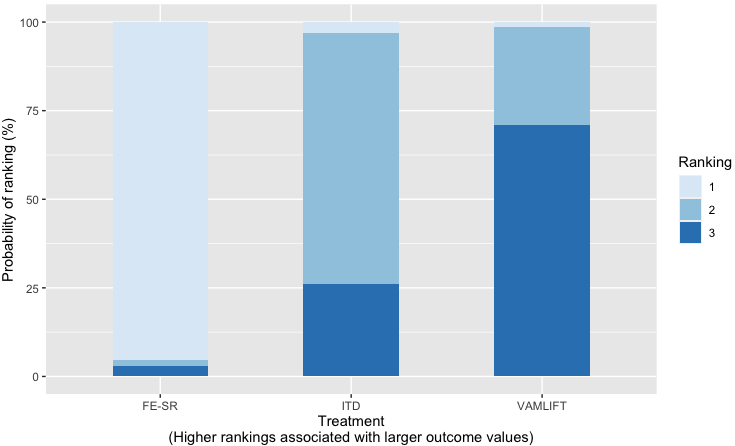
*Rankogram plot* *Forest plot*

*Heat plot*


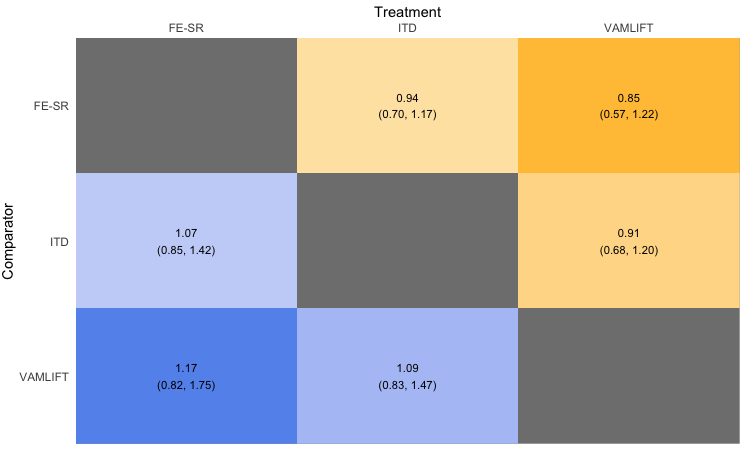


Bowel incontinence (simple anal fistula)

***Table S3.*** *SUCRA ranking probabilities among treatment comparisons for minimising bowel incontinence in patients with simple anal fistula*

| **Treatment** | **No. of trials** | **Total participants** | **SUCRA value (rank)** |
| --- | --- | --- | --- |
| **Sensitivity analysis*** | | | |
| LIFT | 4 | 101 | 82.4 (1) |
| LIFT combined with adipose-derived stem cells | 1 | 30 | 69.9 (2) |
| Incision-thread drawing | 1 | 15 | 64.9 (3) |
| Fistulotomy with marsupialisation | 5 | 216 | 59.6 (4) |
| Radiofrequency fistulectomy | 1 | 10 | 50.2 (5) |
| Fistulotomy | 9 | 322 | 44.1 (6) |
| Collagen plug | 1 | 31 | 41.6 (7) |
| Fistulectomy with primary sphincter reconstruction | 1 | 15 | 40.8 (8) |
| Seton | 1 | 46 | 34.6 (9) |
| Advancement flap | 1 | 29 | 33.7 (10) |
| Fistulectomy | 5 | 228 | 28.2 (11) |

LIFT, ligation of the inter-sphincteric fistula tract; SUCRA, surface under the cumulative ranking.

*Includes all treatments.


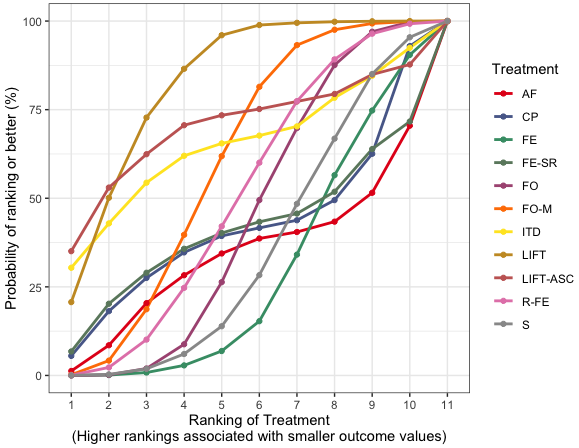

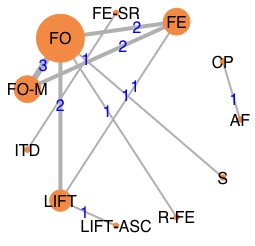
*Network plot* *SUCRA curve*

*
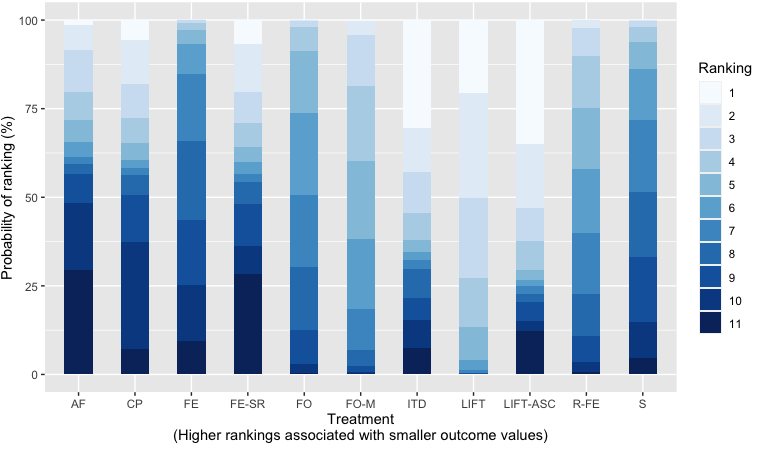
Rankogram plot*

*Forest plot*


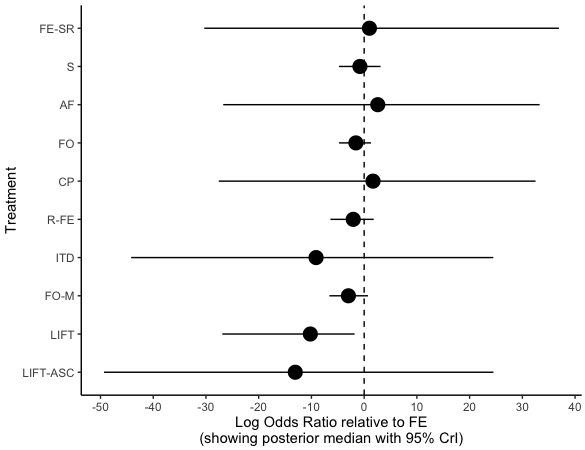


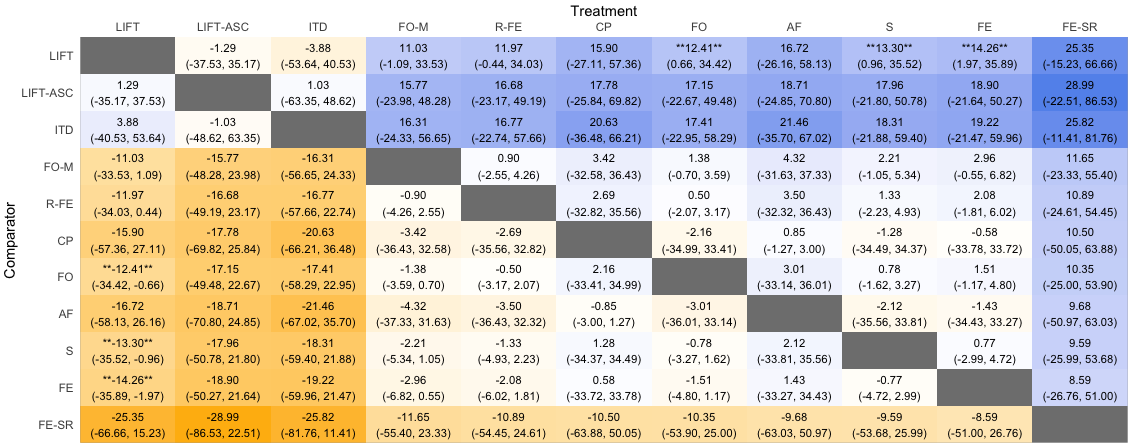
*Heat plot*

Short-term success (≤6 months after surgery) for complex anal fistula

***Table S4.*** *SUCRA ranking probabilities among treatment comparisons for short-term success in patients with complex anal fistula*

| **Treatment** | **No. of trials** | **Total participants** | **SUCRA value (rank)** |
| --- | --- | --- | --- |
| **Sensitivity analysis*** | | | |
| Modified fistulotomy | 1 | 21 | 61.9 (1) |
| Collagen plug combined with a seton | 1 | 20 | 58.7 (2) |
| Seton | 2 | 43 | 57.8 (3) |
| LIFT combined with topical platelet-rich plasma | 1 | 49 | 52.6 (4) |
| LIFT combined with a collagen plug | 1 | 117 | 52.4 (5) |
| Fistulectomy with primary sphincter reconstruction | 1 | 22 | 51.6 (6) |
| Fibrin glue | 5 | 133 | 50.9 (7) |
| Fistulotomy combined with a seton | 1 | 38 | 49.9 (8) |
| Adipose-derived stem cells | 1 | 64 | 49.7 (9) |
| Adipose-derived stem cells combined with fibrin glue | 1 | 60 | 48.4 (10) |
| Video-assisted anal fistula treatment | 2 | 59 | 47.1 (11) |
| Advancement flap | 5 | 148 | 44.3 (12) |
| LIFT | 5 | 256 | 43.8 (13) |
| Collagen plug | 3 | 91 | 42.6 (14) |
| Adipose-derived stem cells combined with a collagen plug | 2 | 44 | 38.2 (15) |

LIFT, ligation of the inter-sphincteric fistula tract; SUCRA, surface under the cumulative ranking.

*Includes all treatments.

*Network plot SUCRA curve*

*
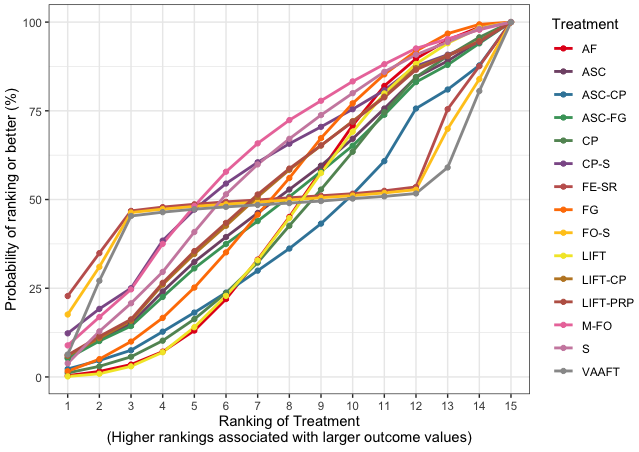

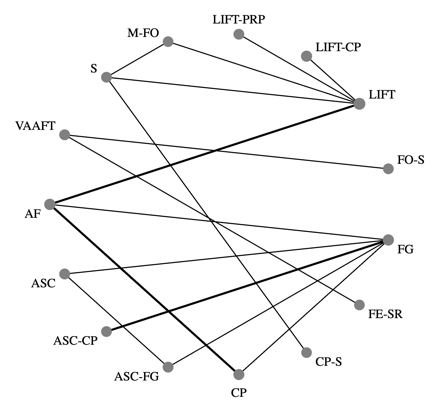
*

*
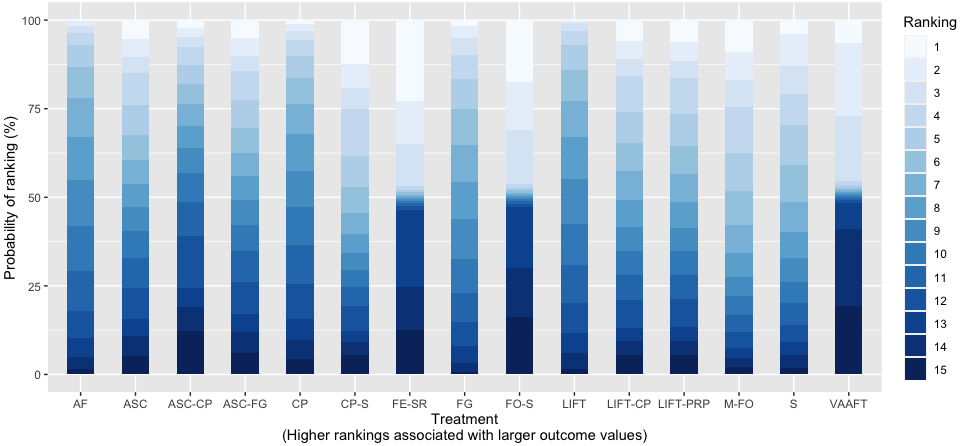
Rankogram plot*


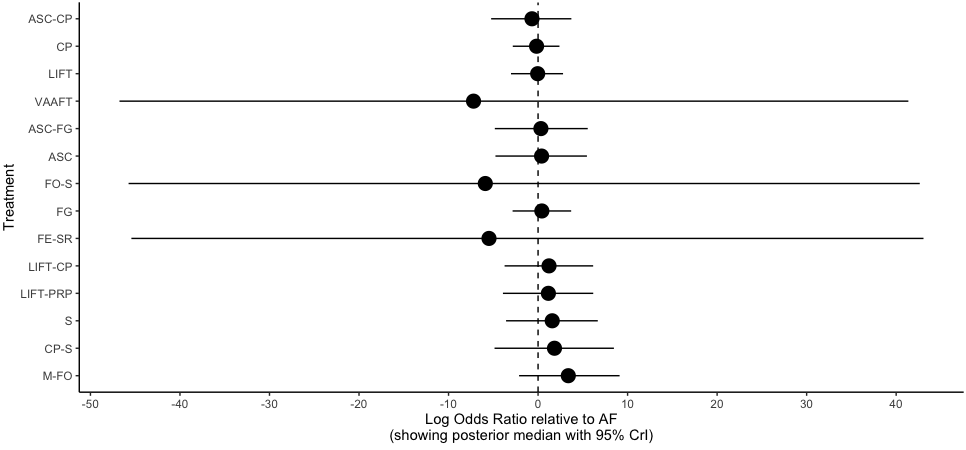
*Forest plot*


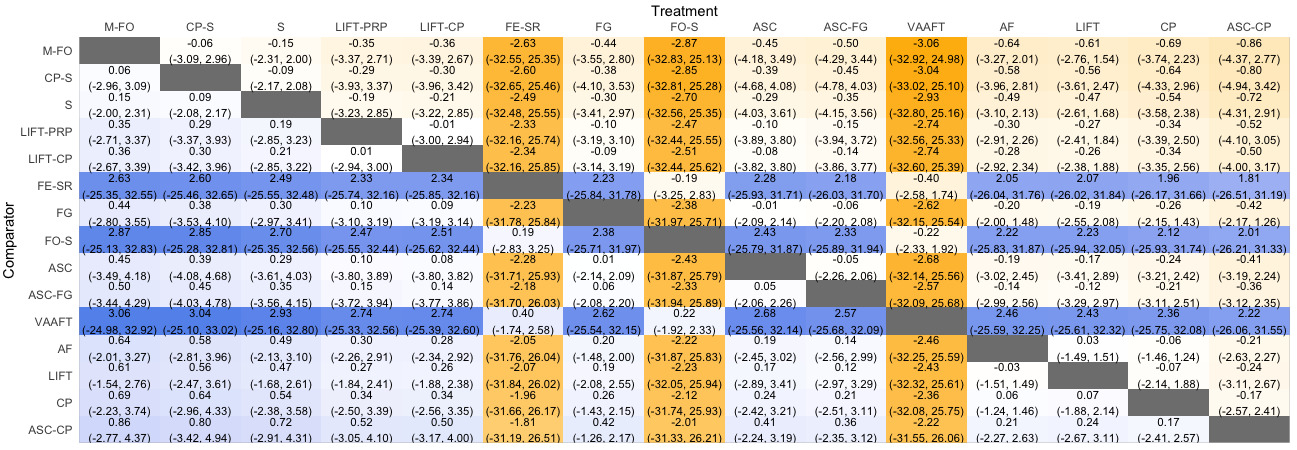
*Heat plot*

Long-term success (>6 months after surgery) for complex anal fistula

***Table S5.*** *SUCRA ranking probabilities among treatment comparisons for long-term success in patients with complex anal fistula*

| **Treatment** | **No. of trials** | **Total participants** | **SUCRA value (rank)** |
| --- | --- | --- | --- |
| **Sensitivity analysis*** | | | |
| Modified fistulotomy | 1 | 21 | 67.4 (1) |
| Adipose-derived stem cells combined with a collagen plug | 1 | 20 | 60.9 (2) |
| Topical platelet-rich plasma following fistula drainage | 1 | 10 | 60.2 (3) |
| Seton | 2 | 47 | 59.3 (4) |
| LIFT | 3 | 99 | 57.1 (5) |
| Fibrin glue followed by fistula closure surgery | 1 | 25 | 53.4 (6) |
| Adipose-derived stem cells | 1 | 64 | 51.2 (7) |
| Fibrin glue with antibiotics followed by fistula closure surgery | 1 | 26 | 50.5 (8) |
| Fibrin glue with antibiotics | 1 | 24 | 48.1 (9) |
| Topical platelet-rich plasma | 3 | 89 | 46.5 (10) |
| Fibrin glue | 3 | 99 | 46.2 (11) |
| Adipose-derived stem cells combined with fibrin glue | 1 | 60 | 45.5 (12) |
| Fistulectomy with primary sphincter reconstruction | 1 | 22 | 41.9 (13) |
| Advancement flap | 5 | 173 | 41.6 (14) |
| Video-assisted anal fistula treatment | 1 | 22 | 38.7 (15) |
| Collagen plug | 3 | 86 | 31.5 (16) |

LIFT, ligation of the inter-sphincteric fistula tract; SUCRA, surface under the cumulative ranking.

*Includes all treatments.


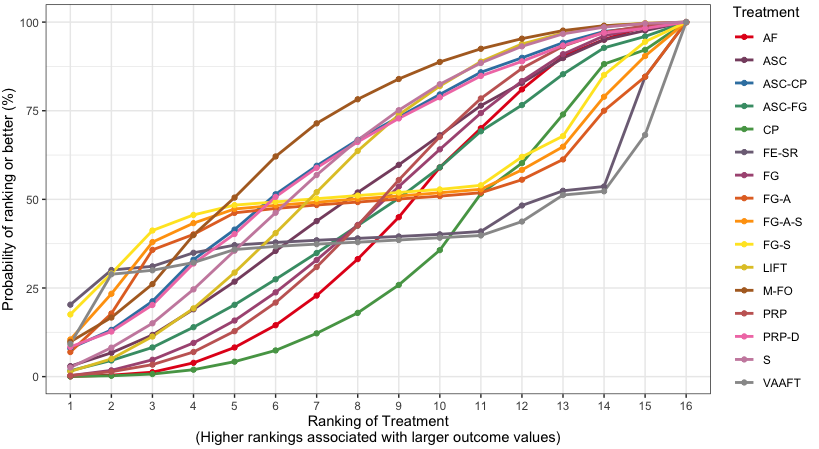

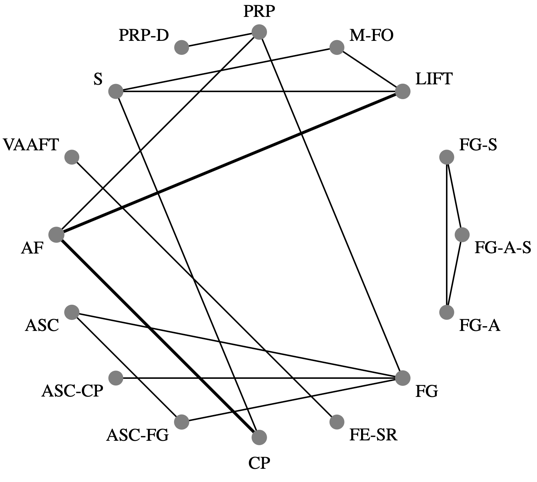
*Network plot* *SUCRA curve*

*
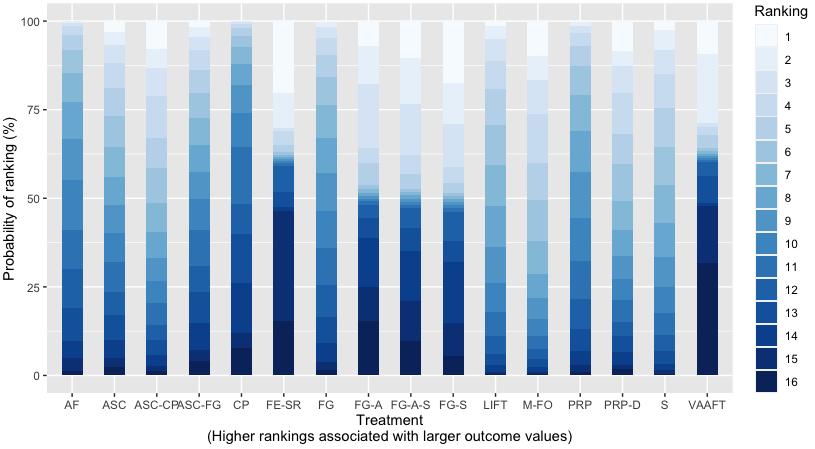
Rankogram plot*


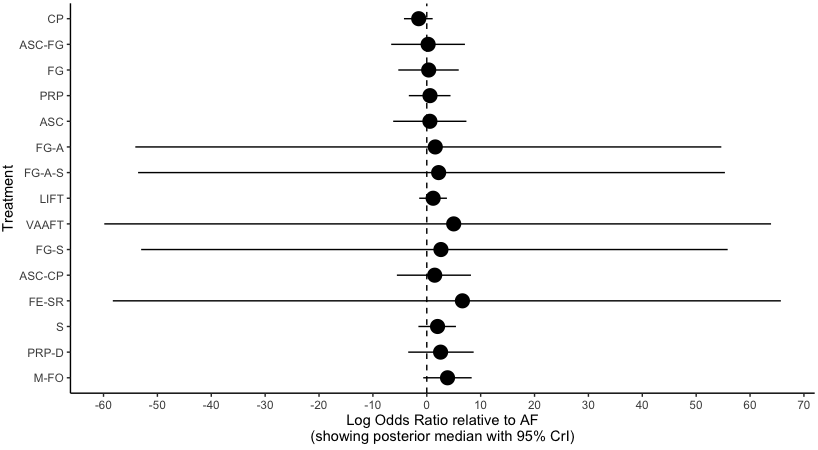
*Forest plot*

*Heat plot*


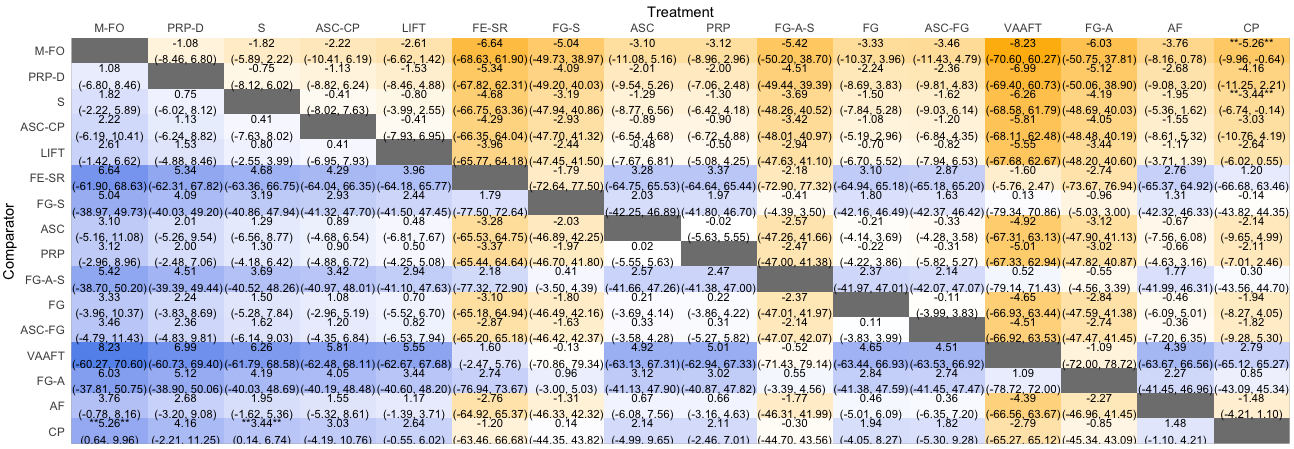


Bowel incontinence (complex anal fistula)

***Table S6.*** *SUCRA ranking probabilities among treatment comparisons for minimising bowel incontinence in patients with complex anal fistula*

| **Treatment** | **No. of trials** | **Total participants** | **SUCRA value (rank)** |
| --- | --- | --- | --- |
| **Sensitivity analysis*** | | | |
| LIFT | 6 | 191 | 87.8 (1) |
| LIFT combined with topical platelet-rich plasma | 1 | 49 | 67.7 (2) |
| Fibrin glue | 2 | 30 | 67.3 (3) |
| “JUMP”** | 1 | 65 | 65.5 (4) |
| Video-assisted anal fistula treatment | 1 | 23 | 62.4 (5) |
| Modified fistulotomy | 1 | 25 | 57.9 (6) |
| Seton | 3 | 186 | 57.7 (7) |
| Fibrin glue with antibiotics followed by fistula closure surgery | 1 | 26 | 54.2 (8) |
| Fibrin glue followed by fistula closure surgery | 1 | 25 | 49.3 (9) |
| Collagen plug | 2 | 58 | 44.8 (10) |
| Fistulotomy with marsupialisation | 1 | 22 | 44.5 (11) |
| Fistulectomy | 2 | 120 | 44.3 (12) |
| Fistulotomy | 1 | 45 | 42.9 (13) |
| Fistulectomy with primary sphincter reconstruction | 1 | 22 | 39.3 (14) |
| Advancement flap | 6 | 177 | 38.3 (15) |
| Fistulotomy with primary sphincter reconstruction | 1 | 28 | 35.3 (16) |
| Fibrin glue with antibiotics | 1 | 24 | 24.0 (17) |
| Suture dragging with pad compression | 1 | 30 | 17.1 (18) |

LIFT, ligation of the inter-sphincteric fistula tract; SUCRA, surface under the cumulative ranking.

*Includes all treatments.

** Inversion of the fistula tract.


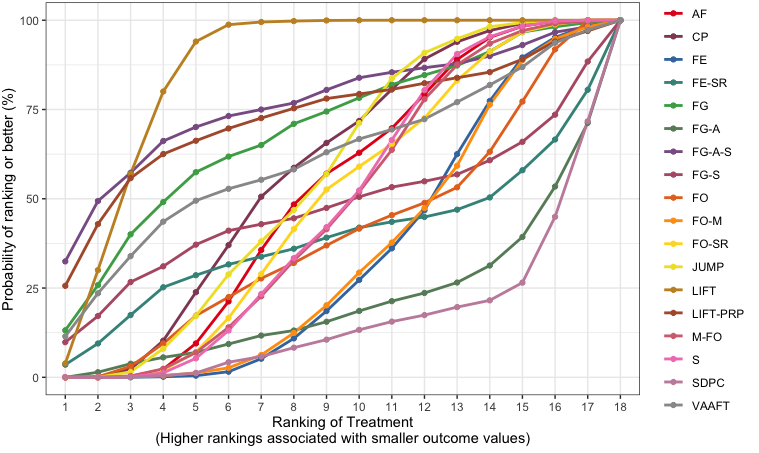

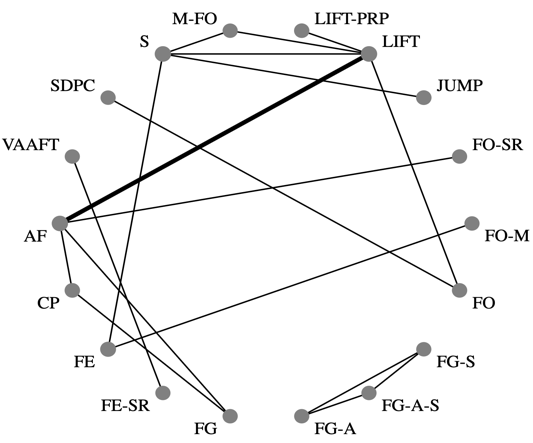
*Network plot SUCRA curve*


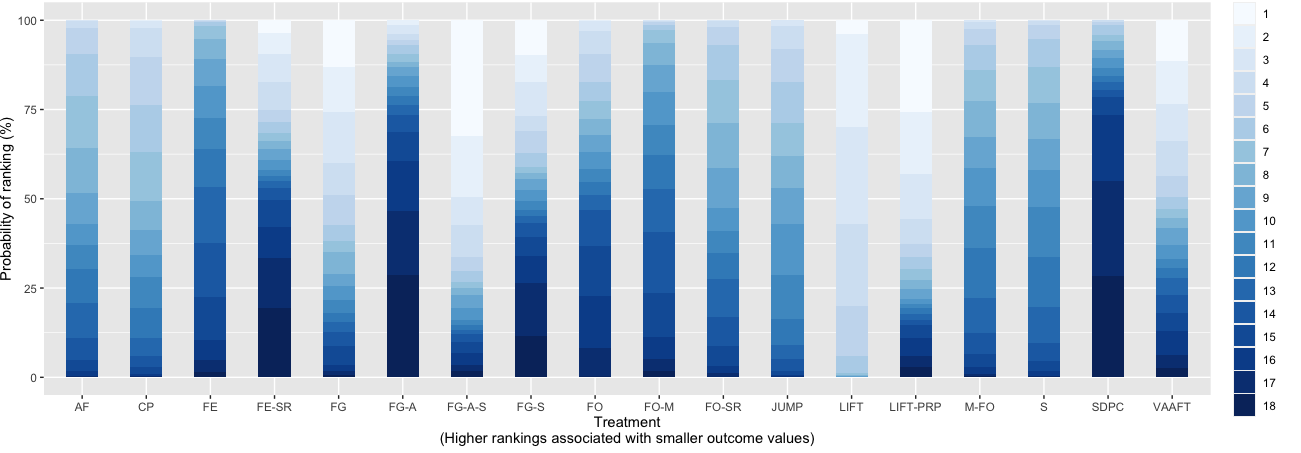
*Rankogram plot*

**
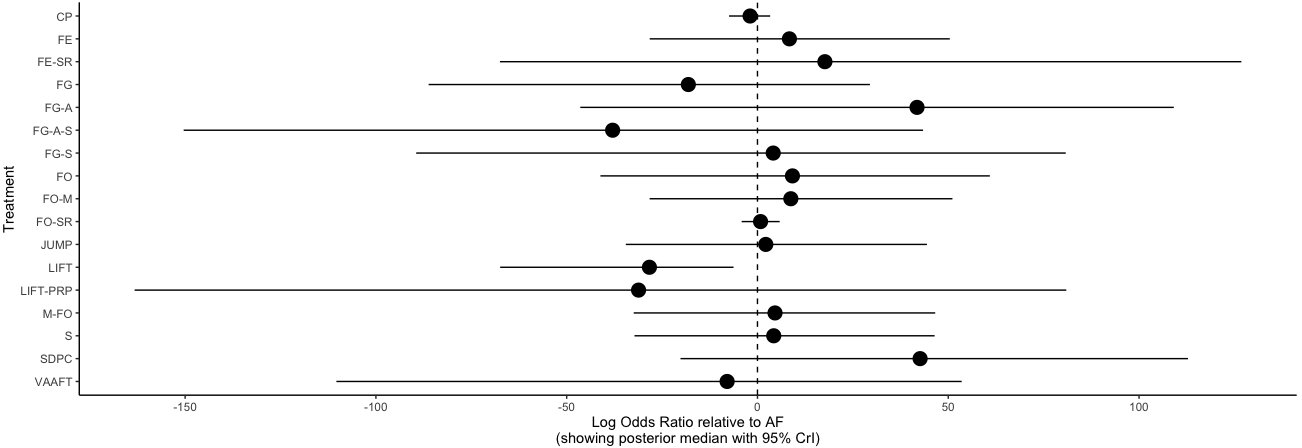
***Forest plot*

*Heat plot*


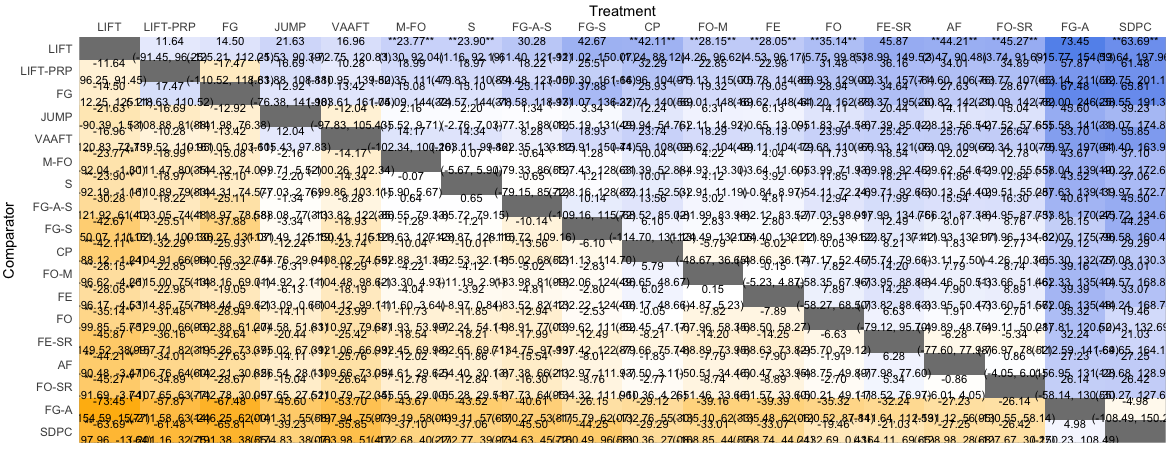


**Secondary outcomes**

Hospital length of stay

***Table S7.*** *SUCRA ranking probabilities among treatment comparisons for minimising hospital length of stay in patients with simple anal fistula in the overall and sensitivity analyses*

| **Treatment** | **No. of trials** | **Total participants** | **SUCRA value (rank)** |
| --- | --- | --- | --- |
| **Overall analysis*** | | | |
| Fistulotomy with marsupialisation | 2 | 131 | 76.0 (1) |
| Fistulotomy | 2 | 132 | 24.0 (2) |
| **Sensitivity analysis**** | | | |
| Fistulotomy with marsupialisation | 2 | 131 | 60.0 (1) |
| Video-assisted modified LIFT | 1 | 37 | 59.0 (2) |
| Seton | 1 | 46 | 54.6 (3) |
| LIFT | 1 | 45 | 54.3 (4) |
| Fistulotomy | 4 | 196 | 54.2 (5) |
| Advancement flap | 1 | 10 | 50.6 (6) |
| Incision-thread drawing | 2 | 45 | 43.9 (7) |
| Fistulectomy | 1 | 45 | 42.2 (8) |
| Fistulectomy with primary sphincter reconstruction | 1 | 15 | 31.3 (9) |

LIFT, ligation of the inter-sphincteric fistula tract; SUCRA, surface under the cumulative ranking.

*Including only treatments assessed in more than one trial.

**Includes all treatments.

^
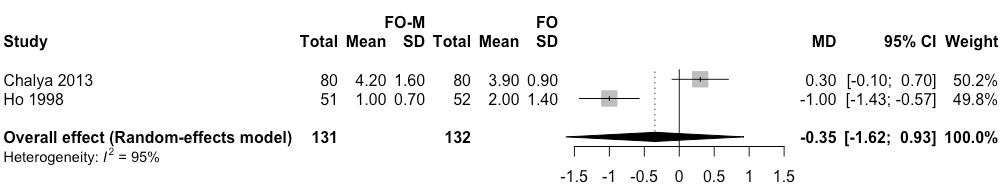
^*Overall analysis**

*Sensitivity analysis***


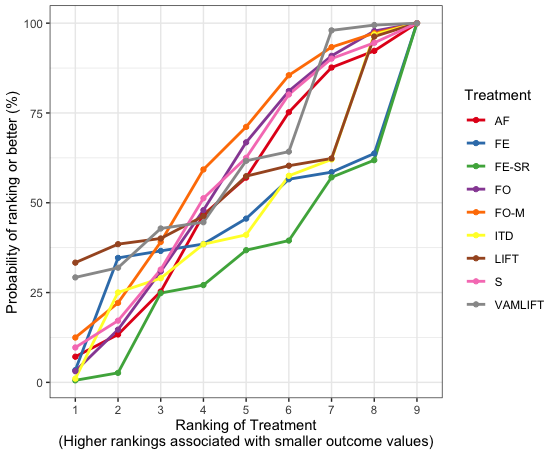
*Network plot SUCRA curve*


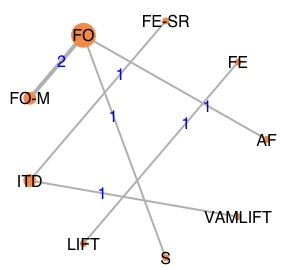


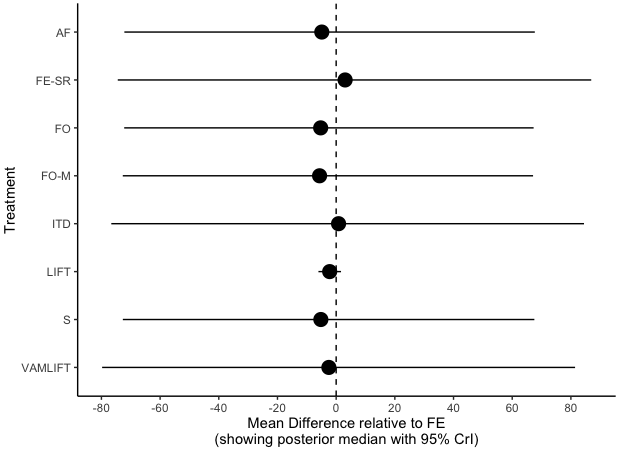
*Rankogram plot Forest plot*


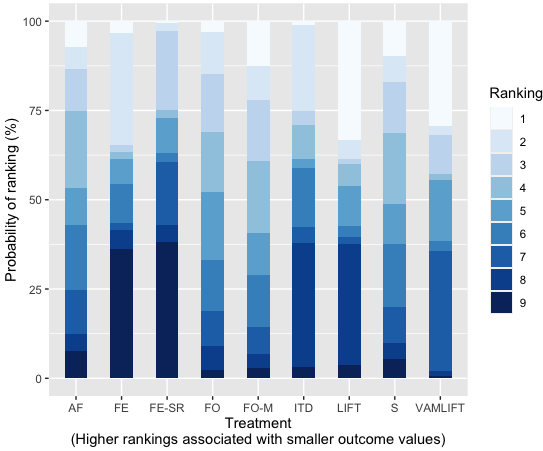


***Table S8.*** *SUCRA ranking probabilities among treatment comparisons for minimising hospital length of stay in patients with complex anal fistula*

| **Treatment** | **No. of trials** | **Total participants** | **SUCRA value (rank)** |
| --- | --- | --- | --- |
| **Sensitivity analysis*** | | | |
| Fistulotomy combined with a seton | 1 | 38 | 59.7 (1) |
| Fistulotomy with marsupialisation | 1 | 22 | 54.1 (2) |
| Fistulectomy | 1 | 24 | 53.4 (3) |
| Video-assisted anal fistula treatment | 1 | 37 | 32.8 (4) |

LIFT, ligation of the inter-sphincteric fistula tract; SUCRA, surface under the cumulative ranking.

*Includes all treatments.


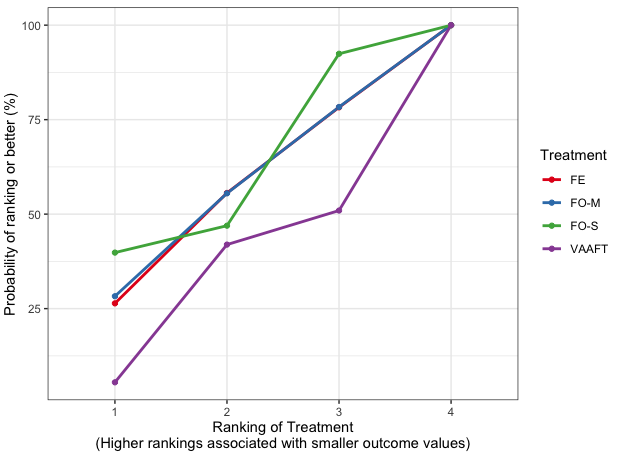

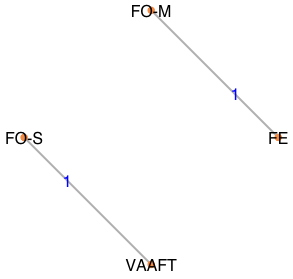
*Network plot SUCRA curve*


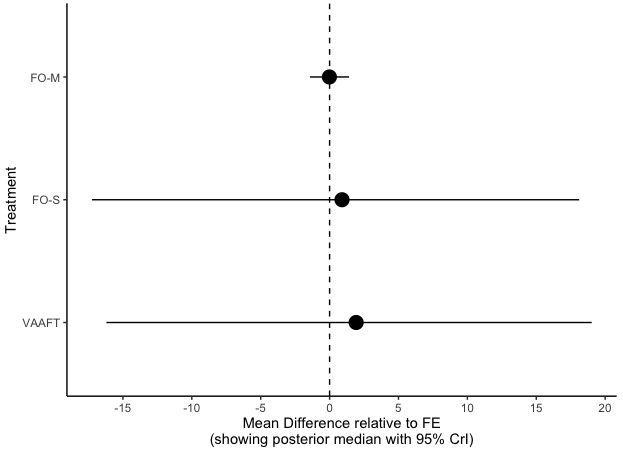

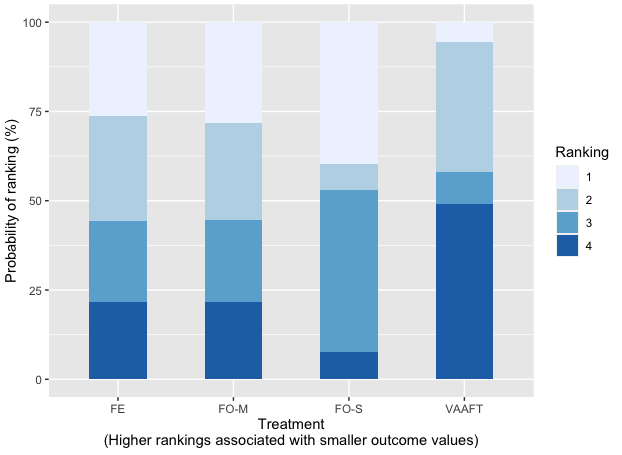
*Rankogram plot Forest plot*

*Overall post-operative complications*

***Table S9.*** *SUCRA ranking probabilities among treatment comparisons for minimising rates of overall post-operative complications in patients with simple anal fistula in the overall and sensitivity analyses*

| **Treatment** | **No. of trials** | **Total participants** | **SUCRA value (rank)** |
| --- | --- | --- | --- |
| **Overall analysis*** | | | |
| Fistulotomy with marsupialisation | 2 | 81 | 83.9 (1) |
| Fistulotomy | 2 | 82 | 16.1 (2) |
| **Sensitivity analysis**** | | | |
| LIFT | 1 | 35 | 76.1 (1) |
| Collagen plug | 1 | 31 | 63.6 (2) |
| Modified fistulotomy | 1 | 63 | 60.4 (3) |
| Fistulotomy with marsupialisation | 2 | 81 | 59.0 (4) |
| Advancement flap | 1 | 29 | 55.5 (5) |
| Fistulotomy | 2 | 82 | 44.5 (6) |
| LIFT combined with adipose-derived stem cells | 1 | 35 | 24.5 (7) |
| Seton | 1 | 46 | 17.9 (8) |

LIFT, ligation of the inter-sphincteric fistula tract; SUCRA, surface under the cumulative ranking.

*Including only treatments assessed in more than one trial.

**Includes all treatments.

^
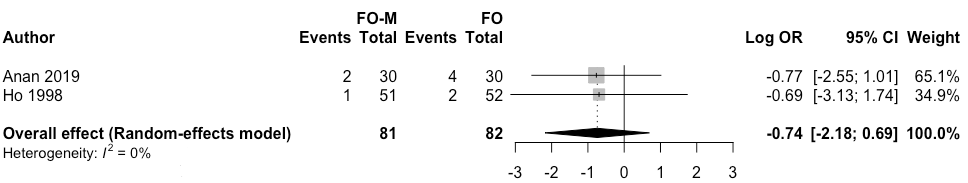
^*Overall analysis^#^*

*Sensitivity analysis*

*
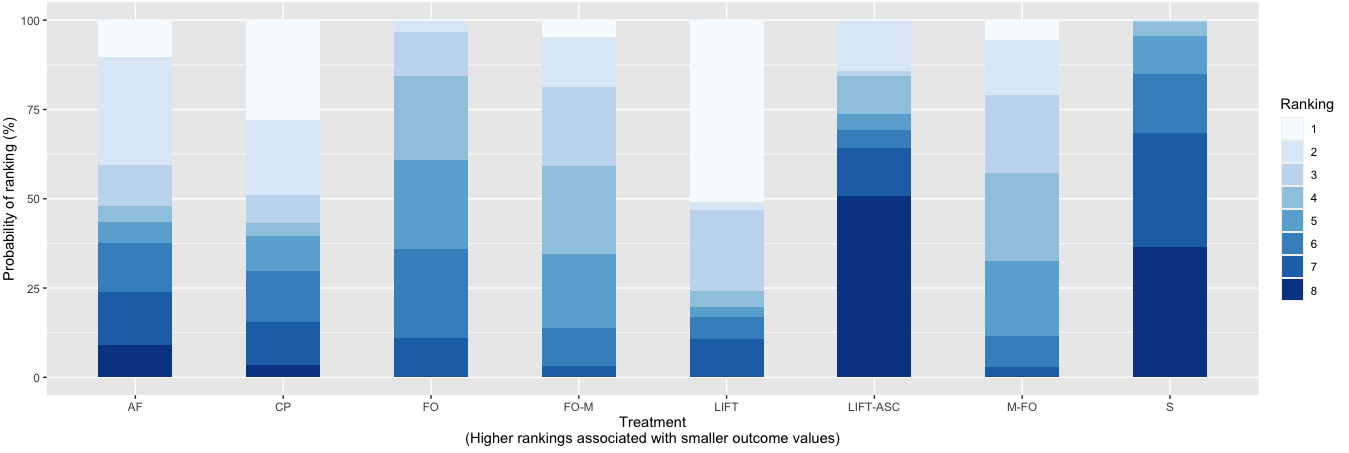
*
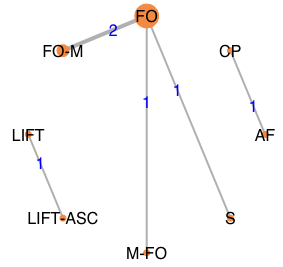
*Network plot Rankogram plot*

*
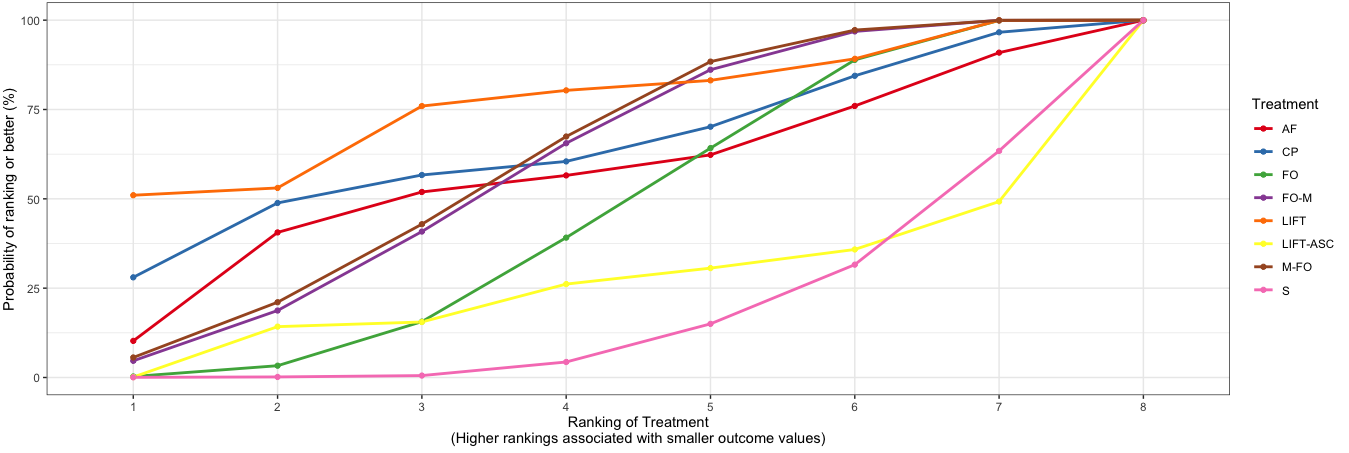
SUCRA curve*


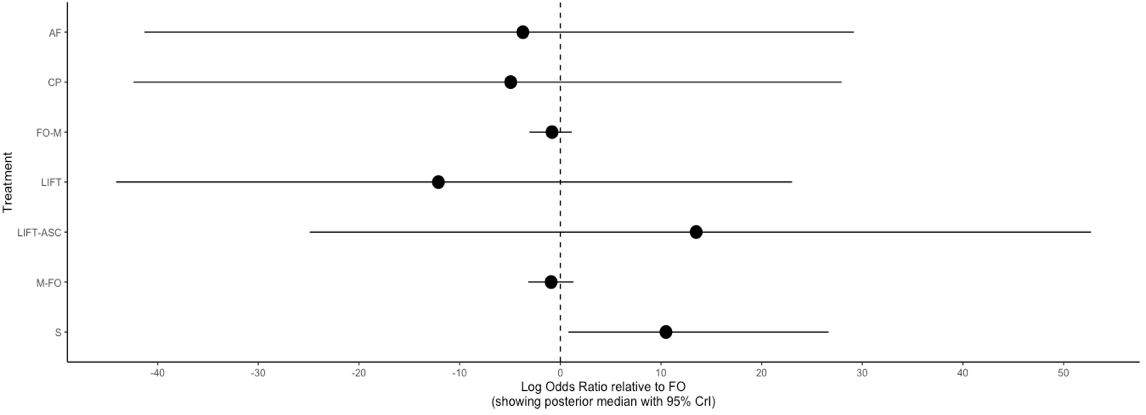
*Forest plot*

***Table S10.*** *SUCRA ranking probabilities among treatment comparisons for minimising rates of overall post-operative complications in patients with complex anal fistula in the overall and sensitivity analyses*

| **Treatment** | **No. of trials** | **Total participants** | **SUCRA value (rank)** |
| --- | --- | --- | --- |
| **Overall analysis*** | | | |
| LIFT | 2 | 60 | 78.0 (1) |
| Advancement flap | 2 | 48 | 22.0 (2) |
| **Sensitivity analysis**** | | | |
| Topical platelet-rich plasma | 1 | 49 | 90.0 (1) |
| Adipose-derived stem cells | 1 | 64 | 80.6 (2) |
| Seton | 1 | 23 | 62.3 (3) |
| Fistulotomy | 1 | 30 | 60.4 (4) |
| LIFT | 5 | 249 | 59.1 (5) |
| Modified fistulotomy | 1 | 21 | 57.6 (6) |
| Advancement flap | 5 | 155 | 53.1 (7) |
| Collagen plug | 1 | 33 | 52.7 (8) |
| Fibrin glue | 1 | 84 | 48.2 (9) |
| Adipose-derived stem cells combined with fibrin glue | 1 | 60 | 47.7 (10) |
| Adipose-derived stem cells combined with a collagen plug | 1 | 24 | 42.4 (11) |
| Suture dragging and pad compression | 1 | 30 | 31.5 (12) |
| LIFT combined with topical platelet-rich plasma | 1 | 49 | 24.6 (13) |
| Fistulotomy with primary sphincter reconstruction | 1 | 28 | 21.0 (14) |
| LIFT combined with a collagen plug | 1 | 117 | 18.8 (15) |

LIFT, ligation of the inter-sphincteric fistula tract; SUCRA, surface under the cumulative ranking.

*Including only treatments assessed in more than one trial.

**Includes all treatments.


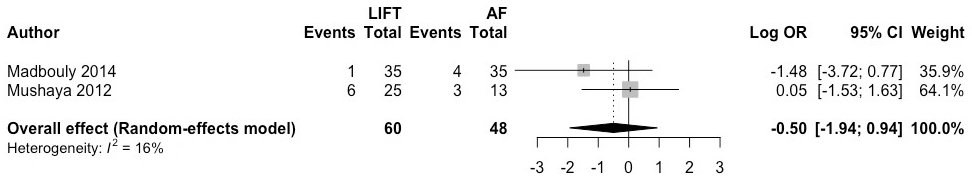
*Overall analysis^#^*

*Sensitivity analysis*


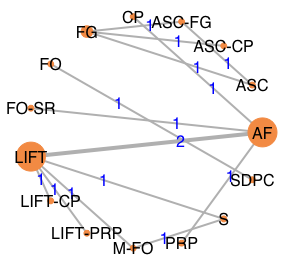
*Network plot Forest plot*


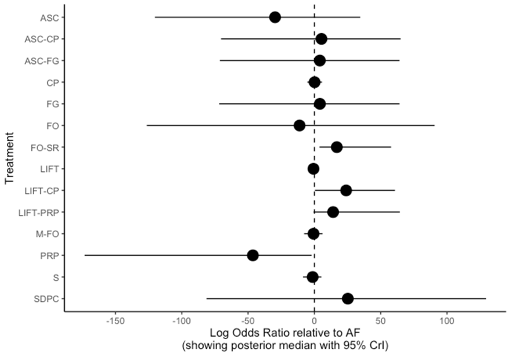


*SUCRA curve*


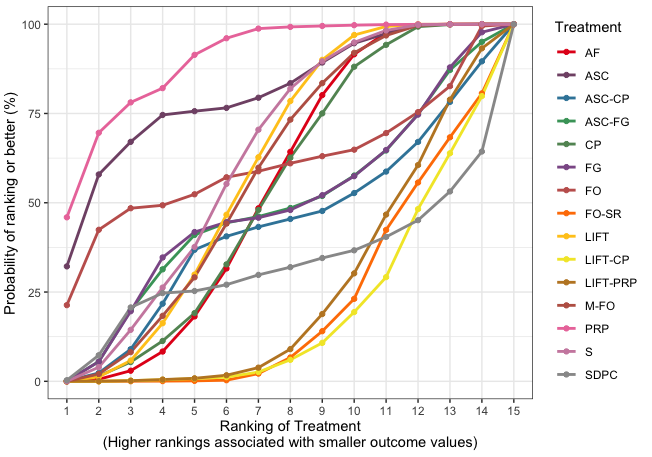


*Rankogram plot*


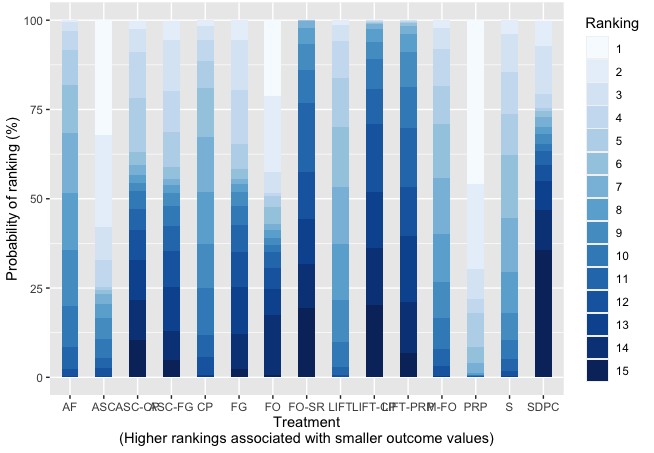


# **Supplementary Appendix S7.** Narrative summary of sensitivity analyses results for each outcome when all treatments were included

**Short and long-term success**

In agreement with the overall analysis, no significant differences were observed between the five treatments in which rates of short-term success were described. Similar results were also observed when long-term success were considered, with the three treatments assessed showing similar efficacy profiles.

In contrast to the overall results, a modified fistulotomy procedure, devised by Elshamy and colleagues[^70^](https://paperpile.com/c/x9kTWR/SVO5), instead ranked as the best treatment for success of anal fistula healing in both the short-term (95.6% of comparisons; n=1 trial with 21 participants; **Table S4**) and long-term (73.7% of comparisons; n=1 trial with 21 participants; **Table S5**) for patients with complex anal fistulae. However, there were no differences between any of the treatments in terms of short and long-term success rates.

**Bowel incontinence**

Not dissimilar from the overall analysis, LIFT consistently ranked as the best procedure for minimising bowel incontinence in both patients with simple (84.5% of comparisons; n=4 trials with 101 participants; **Table S3**) and complex anal fistulae (86.1% of comparisons; n=6 trials with 191 participants; **Table S6**) when all treatments were considered. In the setting of simple anal fistula, LIFT resulted in significantly lower rates of bowel incontinence compared with fistulotomy (log OR -12.4, 95% CrI: -34.4 to -0.7), fistulectomy (-14.3, 95% CrI: -35.9 to -2.0), and seton (log OR -13.3, 95% CrI: -35.5 to -1.0). Regarding complex anal fistula, LIFT was associated with significantly lower bowel incontinence rates relative to fistulotomy with marsupialisation (log OR -25.7, 95% CrI: -78.5 to -2.8), fistulectomy (log OR -25.3, 95% CrI: -78.1 to -3.3), collagen plug (log OR -40.4, 95% CrI: -120.6 to 2-.9), fistulotomy (log OR -38.2, 95% CrI: -134.6 to -6.1), advancement flap (log OR -42.1, 95% CrI: -122.5 to -5.1), fistulotomy with primary sphincter reconstruction (log OR -43.0, 95% CrI: -123.0 to -6.1), and suture dragging with pad compression (log OR -78.3, 95% CrI: -170.0 to -21.3).

**Hospital length of stay**

Congruent with the overall analysis, there were no significant differences between the nine treatments in which hospital LOS were evaluated for patients with simple anal fistula. However, of these treatments, fistulotomy with marsupialisation again ranked the best for minimising hospital LOS (60.0% of comparisons; n=2 trials with 131 participants; **Table S7**). This was also the case for complex anal fistula patients, whereby none of the four treatments differed from one another. Although, fistulotomy combined with a seton performed best for minimising duration of hospitalisation (59.7% of comparisons; n=1 trial with 38 participants; **Table S8**).

**Overall post-operative complications**

Rates of overall post-operative complications did not differ between any of the analysed treatments for both patients with simple and complex anal fistula. However, contrary to the overall results, LIFT was the best performing procedure for minimising post-operative complication rates in patients with simple anal fistulae (76.1% of comparisons; n=1 trial with 35 participants; **Table S9**). Of interest, relative to other treatments, the use of topical platelet-rich plasma ranked best for minimising rates of post-operative complications in patients with complex anal fistulae (90.0% of comparisons), although its utility was assessed in only one trial consisting of 49 participants (**Table S10)**.

# **Supplementary Appendix S8.** Inconsistency analysis across treatment comparisons for each outcome

**Primary outcomes**

Simple anal fistula

*Short-term success (≤6 months after surgery) Bowel incontinence*


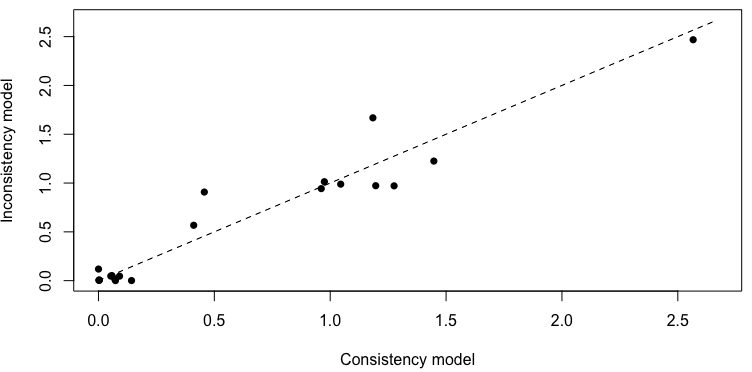

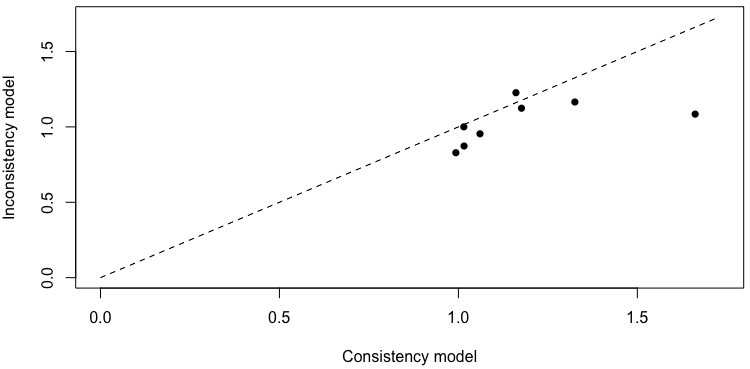


Complex anal fistula

*Short-term success (≤6 months after surgery) Long-term success (>6 months after surgery)*


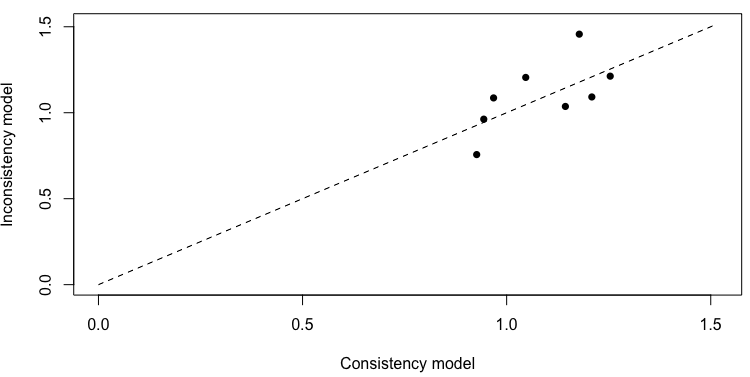

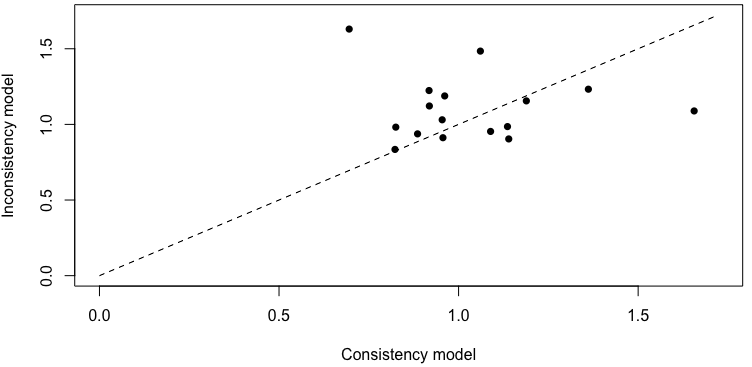


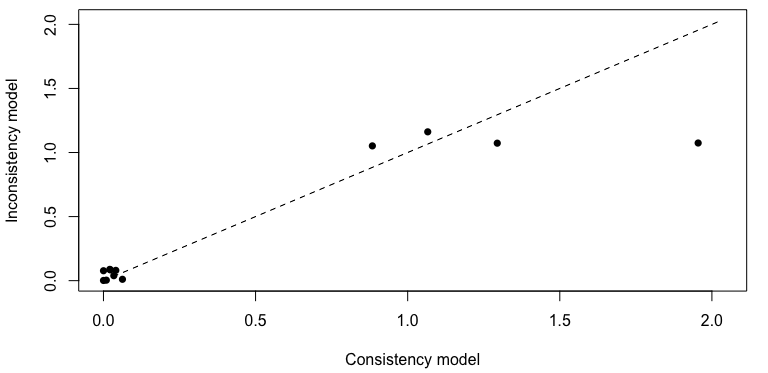
Bowel incontinence

**Secondary outcomes**

*
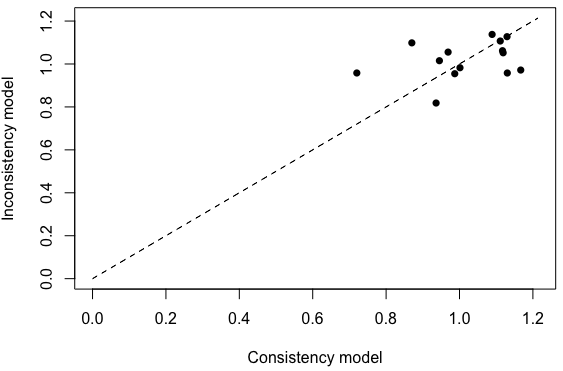
*Simple anal fistula


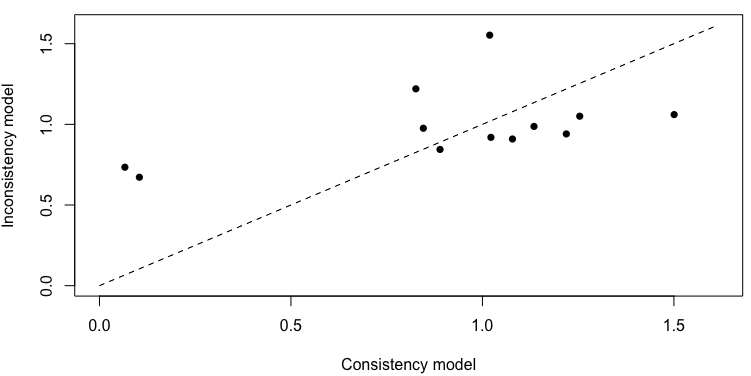
*Hospital length of stay Overall post-operative complications*

Complex anal fistula


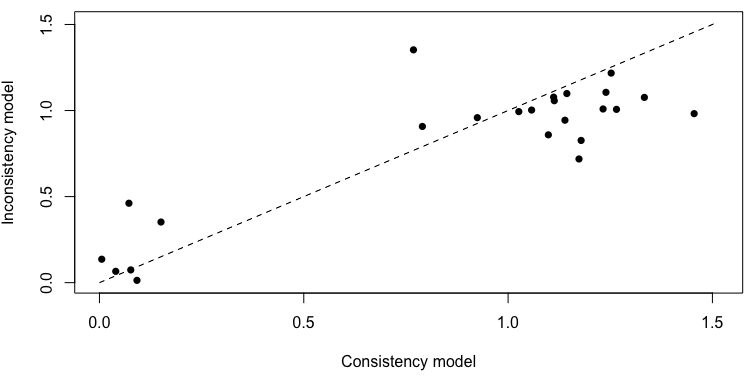

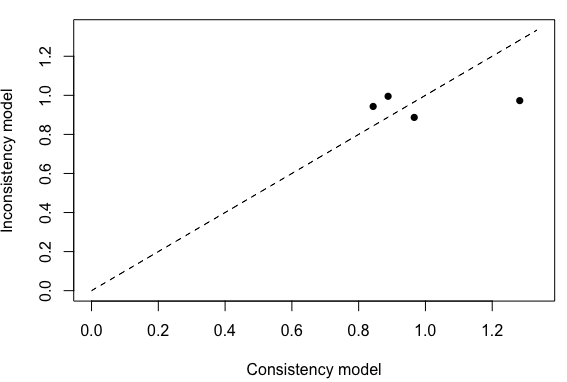
*Hospital length of stay* *Overall post-operative complications*

# **Supplementary Appendix S9.** Heterogeneity analysis among each direct comparison of treatments for each outcome

**Primary outcomes**

Simple anal fistula

*Short-term success (≤6 months after surgery)*

| **Direct comparison of treatments** | **Number of trials** | **I^2^ (%)** |
| --- | --- | --- |
| LIFT *vs.* FE | 2 | 75 |

LIFT, ligation of the inter-sphincteric fistula tract; FE, fistulectomy
*Bowel incontinence*

| **Direct comparison of treatments** | **Number of trials** | **I^2^ (%)** |
| --- | --- | --- |
| FO-M *vs.* FO | 3 | 19 |
| FO-M *vs.* FE | 2 | 0 |
| FE *vs.* FO | 2 | 0 |
| LIFT *vs.* FO | 2 | 0 |

FE, fistulectomy; FO, fistulotomy; FO-M, fistulotomy with marsupialisation; LIFT, ligation of the inter-sphincteric fistula tract.

Complex anal fistula

*Short-term success (≤6 months after surgery)*

| **Direct comparison of treatments** | **Number of trials** | **I^2^ (%)** |
| --- | --- | --- |
| ASC-CP *vs.* FG | 2 | 0 |
| LIFT *vs.* AF | 2 | 82 |
| AF *vs.* CP | 2 | 0 |

AF, advancement flap; ASC-CP, adipose-derived stem cells combined with a collagen plug; CP, collagen plug; FG, fibrin glue; LIFT, ligation of the inter-sphincteric fistula tract.

*Long-term success (>6 months after surgery)*

| **Direct comparison of treatments** | **Number of trials** | **I^2^ (%)** |
| --- | --- | --- |
| LIFT *vs.* AF | 2 | 0 |
| AF *vs.* CP | 2 | 86 |

AF, advancement flap; CP, collagen plug; LIFT, ligation of the inter-sphincteric fistula tract.

*Bowel incontinence*

| **Direct comparison of treatments** | **Number of trials** | **I^2^ (%)** |
| --- | --- | --- |
| LIFT *vs.* AF | 3 | 0 |

AF, advancement flap; LIFT, ligation of the inter-sphincteric fistula tract.

**Secondary outcomes**

Simple anal fistula

*Hospital length of stay*

| **Direct comparison of treatments** | **Number of trials** | **I^2^ (%)** |
| --- | --- | --- |
| FO-M *vs.* FO | 2 | 95 |

FO, fistulotomy; FO-M, fistulotomy with marsupialisation.

*Overall post-operative complications*

| **Direct comparison of treatments** | **Number of trials** | **I^2^ (%)** |
| --- | --- | --- |
| FO-M *vs.* FO | 2 | 0 |

FO, fistulotomy; FO-M, fistulotomy with marsupialisation.

Complex anal fistula

*Hospital length of stay*

Heterogeneity could not be determined as no two treatments evaluating hospital length of stay in patients with complex anal fistula were compared in more than one study.

*Overall post-operative complications*

| **Direct comparison of treatments** | **Number of trials** | **I^2^ (%)** |
| --- | --- | --- |
| LIFT *vs.* AF | 2 | 0 |

AF, advancement flap; LIFT, ligation of the inter-sphincteric fistula tract.

**Supplementary Appendix S10.** Individual author definitions for anal fistula healing and bowel incontinence among included trials

| **First author (year)** | **Primary outcome definitions** | |
| --- | --- | --- |
|  | **Anal fistula healing** | **Bowel incontinence (solid/liquid/gas)** |
| A ba-bai-ke-re (2010) | Closure of all external openings, absence of drainage without further intervention, and no abscess formation | Solid stool |
| A ba-bai-ke-re (2012) | NS |  |
| Al Sebai (2021) | No discharge from the external wound | Gas |
| Altomare (2011) | Complete cicatrisation of the fistula and the absence of any clinical signs of perianal sepsis | Solid stool |
| Anan (2019) | Complete epithelialisation of the wound, with no evidence of external fistula opening or perianal discharge | Solid stool |
| Bondi (2017) | NS | NS |
| Chalya (2013) | Absence of an unepithelialised surface | Gas and/or solid stool |
| Chen (2005) | NS | NS |
| Cwalinski (2021) | NS |  |
| de la Portilla (2019) | Closure and epithelialisation of the fistula without any reported or evident discharge | NS |
| Dong (2020) | Complete disappearance of all clinical signs and symptoms, with good wound healing | NS |
| Ellis (2006) | NS |  |
| Elshamy (2022) | Complete epithelialisation of the surgical wound, the external fistula opening was closed, and no discharge was experienced | Gas and/or solid stool |
| Filingeri (2004) | NS | Gas and/or solid stool |
| Garcia-Arranz (2020) | Complete re-epithelialisation of external openings with the absence of discharge |  |
| Garcia-Olmo (2009) | Absence of drainage through the external openings (whether occurring spontaneously or under externally applied pressure), and complete re-epithelialisation of the external openings |  |
| Goudar (2020) | NS | Gas or liquid stool |
| Gupta (2003) | NS | Gas |
| Hammond (2009) | NS | NS |
| Han (2016) | Complete closure of all external openings in combination with the absence of symptoms at any time during follow-up |  |
| Hermann (2022) | NS |  |
| Herreros (2012) | Absence of drainage through the external openings, complete re-epithelialisation of the external opening, and absence of collections >2cm on MRI | NS |
| Ho (1998) | NS | Gas or liquid stool |
| Ho (2001) | NS | Gas or liquid stool |
| Ho (2005) | NS | NS |
| Jain (2012) | Absence of any unepithelialised surface | Gas and/or solid stool |
| Kalim (2017) | Complete epithelialisation of the operative area at the anal canal confirmed on physical examination |  |
| Khoshnevis (2022) | NS | Gas, liquid, and/or solid stool |
| Kronborg (1985) | NS | Gas |
| Kumar (2022) | Complete epithelialisation of the wound or healing with granulation | Gas and/or liquid stool |
| Madbouly (2014) | Closed external and internal openings without any discharge | Gas and/or solid stool |
| Madbouly (2021) | Closure of the external opening without any discharge from either the external opening site or the anal canal (self-reported by patients and using direct anoscopy) | Solid stool |
| Mascagni (2018) | Absence of any discharge or abscess | Gas and/or solid stool |
| Mushaya (2012) | NS | NS |
| Nazeer (2012) | NS | NS |
| Nour (2020) | NS | Gas |
| Ortiz (2009) | NS |  |
| Perez (2006) | NS | Gas and/or solid stool |
| Pescatori (2006) | NS | Gas, liquid, and/or solid stool |
| Rezk (2022) | Closure of the internal and external openings, without any discharge | NS |
| Sahakitrungruang (2011) | NS | Solid stool |
| Schwandner (2018) | Closure of the external opening with no evidence of abscess, drainage, or pain |  |
| Singer (2005) | Absence of symptoms | NS |
| Sorenson (2021) | Visual evaluation of an epithelialised wound (with scar or hypergranulation formation) | NS |
| van der Hagen (2011) | No drainage of the previous external opening (with and without finger compression), external orifices appeared healed, and patients were asymptomatic | NS |
| van Koperen (2011) | Closed external and internal openings without discharge and pain | NS |
| Vinay (2017) | NS | NS |
| Wang (2012) | Complete epithelialisation of the wound, with the absence of bleeding, pain, discharge, and subjective symptoms | Liquid and/or solid stool |
| Wang (2021) | Complete epithelialisation of the wound | Gas, liquid, and/or solid stool |
| Wu (2021) | NS | NS |
| Yan (2020) | NS | NS |
| Zhang (2020) | NS | Gas, liquid, and/or solid stool |

MRI, magnetic resonance imaging; NS, not specified.

Cell left blank relate to outcomes that were not measured in the respective study.

**Supplementary Appendix S11.** Duration of follow-up after anal fistula surgery in each study

| **First author (year)** | **Follow-up (months), mean [median] ± SD (range)** | | | |
| --- | --- | --- | --- | --- |
|  | **Experiment treatment** | | **Control treatment** | |
| A ba-bai-ke-re (2010) | CP: 5.7 ± 0.3 | | AF: 6.1 ± 0.1 | |
| A ba-bai-ke-re (2012) | CP-S: 5.3 (4.0-6.5) | | S: 5.7 (4.5-7.0) | |
| Al Sebai (2021) | 6 | | | |
| Altomare (2011) | >12 | | | |
| Anan (2019) | FO-M: 11.5 ± 1.7 | | FO: 11.3 ± 1.1 | |
| Bondi (2017) | 12.6 ± 3.1 | | | |
| Chalya (2013) | 12 | | | |
| Chen (2005) | 6 | | | |
| Cwalinski (2021) | 12 | | | |
| de la Portilla (2019) | 12 | | | |
| Dong (2020) | <0.25 | | | |
| Ellis (2006) | [22] (18-34) | | | |
| Elshamy (2022) | 12 | | | |
| Filingeri (2004) | NS | | | |
| Garcia-Arranz (2020) | 24 | | | |
| Garcia-Olmo (2009) | 2 | | | |
| Goudar (2020) | 2 | | | |
| Gupta (2003) | 3 | | | |
| Hammond (2009) | 27.3 ± 10.6 | | | |
| Han (2016) | LIFT-CP: 6.0 ± 0.1 | | LIFT: 6.1 ± 0.3 | |
| Hermann (2022) | [12] | | | |
| Herreros (2012) | 12 | | | |
| Ho (1998) | FO-M: 10.2 ± 3.6 | | FO: 9.0 ± 2.9 | |
| Ho (2001) | FO: 2.7 ± 2.7 | | S: 2.8 ± 2.0 | |
| Ho (2005) | 15.8 ± 7.2 | | | |
| Jain (2012) | 3 | | | |
| Kalim (2017) | NS | | | |
| Khoshnevis (2022) | NS | | | |
| Kronborg (1985) | 12 | | | |
| Kumar (2022) | 24 | | | |
| Madbouly (2014) | 12 | | | |
| Madbouly (2021) | 12 | | | |
| Mascagni (2018) | 36 | | | |
| Mushaya (2012) | LIFT: 18.2 ± 11.8 | | AF: 23.5 ± 14.5 | |
| Nazeer (2012) | 10 | | | |
| Nour (2020) | 6 | | | |
| Ortiz (2009) | 12 | | | |
| Perez (2006) | 36 (24-52) | | | |
| Pescatori (2006) | FO-M: 10.5 | | FE: 13.8 | |
| Rezk (2022) | LIFT-ASC: 7.3 ± 2.8 | | LIFT: 8.5 ± 2.8 | |
| Sahakitrungruang (2011) | NS | | | |
| Schwandner (2018) | 12 | | | |
| Singer (2005) | FG-A: 25.0 ± 6.2 | FG-S: 28.0 ± 7.7 | | FG-A-S: 27.0 ± 7.8 |
| Sorenson (2021) | 6 | | | |
| van der Hagen (2011) | FG: 50.7 ± 2.9 | | AF: 52.3 ± 4.0 | |
| van Koperen (2011) | 12.2 ± 5.4 | | | |
| Vinay (2017) | 10 | | | |
| Wang (2012) | NS | | | |
| Wang (2021) | 0.25 | | | |
| Wu (2021) | VAMLIFT: 68.7 ± 11.6 | | ITD: 66.7 ± 12.1 | |
| Yan (2020) | FE-S: 39.5 ± 8.1 | | S: 37.8 ± 12.6 | |
| Zhang (2020) | VAAFT: 49.1 ± 11.5 | | FO-S: 34.6 ± 12.0 | |

AF, advancement flap; CP, collagen plug; CP-S, collagen plug combined with a seton; FE, fistulectomy; FE-S, fistulectomy combined with a seton; FG, fibrin glue; FG-A, fibrin glue with antibiotics; FG-S, fibrin glue with fistula closure surgery; FG-A-S, fibrin glue with antibiotics followed by fistula closure surgery; FO, fistulotomy; FO-M, fistulotomy with marsupialisation; FO-S, fistulotomy combined with a seton; ITD, incision-thread drawing; LIFT, ligation of the inter-sphincteric fistula tract; LIFT-ASC, LIFT combined with adipose-derived stem cells; LIFT-CP, LIFT combined with a collagen plug; NS, not specified; SD, standard deviation; S, seton; VAAFT, video-assisted anal fistula treatment; VAMLIFT, video-assisted modified LIFT.
